# Supplementary material for: Enhancing Nutrition and Antenatal Infection Treatment (ENAT) study: protocol of a pragmatic clinical effectiveness study to improve birth outcomes in Ethiopia
Source: BMJ Paediatr Open. 2022 Jan 13;6(1):e001327. doi: 10.1136/bmjpo-2021-001327 (PMC8762145; doi:10.1136/bmjpo-2021-001327)
Supplement: Supplementary data [file bmjpo-2021-001327supp001.pdf]

WEBAPPENDIX

Web Table 1. WHO Recommendations for Antenatal Care

**Nutritional Interventions**	
A.1.1: Counselling about healthy eating and keeping physically active during pregnancy is recommended for pregnant women to stay healthy and to prevent excessive weight gain during pregnancy.	Recommended
A.1.2: In undernourished populations, nutrition education on increasing daily energy and protein intake is recommended for pregnant women to reduce the risk of low-birth-weight neonates.	Context-specific recommendation
A.1.3: In undernourished populations, balanced energy and protein dietary supplementation is recommended for pregnant women to reduce the risk of stillbirths and small-for-gestational-age neonates.	Context-specific recommendation
A.2.1: Daily oral iron and folic acid supplementation with 30 mg to 60 mg of elemental iron and 400 µg (0.4 mg) of folic acid is recommended for pregnant women to prevent maternal anaemia, puerperal sepsis, low birth weight, and preterm birth.	Recommended
A.2.2: Intermittent oral iron and folic acid supplementation with 120 mg of elemental iron and 2800 µg (2.8 mg) of folic acid once weekly is recommended for pregnant women to improve maternal and neonatal outcomes if daily iron is not acceptable due to side-effects, and in populations with an anaemia prevalence among pregnant women of less than 20%.	Context-specific recommendation
A.3: In populations with low dietary calcium intake, daily calcium supplementation (1.5–2.0 g oral elemental calcium) is recommended for pregnant women to reduce the risk of pre-eclampsia.	Context-specific recommendation
A.4: Vitamin A supplementation is only recommended for pregnant women in areas where vitamin A deficiency is a severe public health problem, to prevent night blindness.	Context-specific recommendation
A.5: Zinc supplementation for pregnant women is only recommended in the context of rigorous research.	Context-specific recommendation (research)
A.10: For pregnant women with high daily caffeine intake (more than 300 mg per day), lowering daily caffeine intake during pregnancy is recommended to reduce the risk of pregnancy loss and low-birth-weight neonates.	Context-specific recommendation
**Maternal Assessment**	
B.1.1: Full blood count testing is the recommended method for diagnosing anaemia in pregnancy. In settings where full blood count testing is not available, on-site haemoglobin testing with a haemoglobinometer is recommended over the use of the haemoglobin colour scale as the method for diagnosing anaemia in pregnancy.	Context-specific recommendation
B.1.2: Midstream urine culture is the recommended method for diagnosing asymptomatic bacteriuria (ASB) in pregnancy. In settings where urine culture is not available, on-site midstream urine Gram-staining is recommended over the use of dipstick tests as the method for diagnosing ASB in pregnancy.	Context-specific recommendation
B.2.4: One ultrasound scan before 24 weeks of gestation (early ultrasound) is recommended for pregnant women to estimate gestational age, improve detection of fetal anomalies and multiple pregnancies, reduce induction of labour for post-term pregnancy, and improve a woman's pregnancy experience.	Recommended
**Preventive Measures**	
C.1: A seven-day antibiotic regimen is recommended for all pregnant women with asymptomatic bacteriuria (ASB) to prevent persistent bacteriuria, preterm birth and low birth weight.	Recommended
C.2: Antibiotic prophylaxis is only recommended to prevent recurrent urinary tract infections in pregnant women in the context of rigorous research.	Context-specific recommendation (research)
C.4: In endemic areas, preventive anthelminthic treatment is recommended for pregnant women after the first trimester as part of worm infection reduction programmes.	Context-specific recommendation

Web Table 2. Super Cereal Corn Soya Blend Composition (per daily 200 gm serving)

NUTRIENT	Units	ENAT CSB CONTENT (200 gm)	Recommended Target*
Energy	Kcal	760	250-500kcal per daily serving**
Protein	gm	28	14-18 g**
Fat	%	12%	10-60% of energy
Vitamin A	mcg RE	2076	550-770***
Vitamin D	mcg	22.1	10-15
Vitamin E	mg	16.6	16-19
Vitamin K	mcg	60	72-90
Vitamin B1	mg	0.4	1.2-1.4
Vitamin B2	mg	2.8	1.3-1.6
Vitamin B3	mg	16	14-18
Folic Acid	mcg	220	400-600
Vitamin B6	mg	2.0	1.7-2.0
Vitamin C	mg	180	100-120
Calcium	mg	724	500-1500
Iron	mg	8	22-27
Iodine	mcg	80	209-290
Phosphorous	mg	560	300-700
Zinc	mg	10	15-20

* Targets are results of an Expert Consultation held at Bill and Melinda Gates Foundation.[50]
** Energy balance from macronutrient: portion size can be doubled in settings of high energy gaps, such as maternal malnutrition and where prevalence of low birthweight is high.
*** Micronutrient recommendations were primarily based upon Institute of Medicine (IOM) estimated average requirement (EAR) and recommended dietary allowances (RDA) values.

Web Table 3. Classification of UTI in ENAT study

UTI Terminology	Definition
High-burden growth	bacteriuria of $>10^5$ colony forming units (CFU) per 1mL of urine of a single uropathogen [49],
Intermediate growth	bacteriuria with $>10^3$ -10^5 CFU/mL of a single uropathogen,
Contamination	bacterial growth of $\geq$3 micro-organism OR growth of a non-urinary tract pathogen.
UTI symptoms	dysuria, urinary frequency, urinary urgency, hematuria, abdominal pain, fever, OR flank pain
Symptomatic intermediate growth	women with intermediate burden growth and UTI symptoms (as above)
Asymptomatic bacteriuria	women with high burden bacterial growth without UTI symptoms
Cystitis	women with positive urine culture (high burden or intermediate growth) and symptoms of dysuria, urinary frequency, hematuria, urinary urgency or suprapubic tenderness, without upper urinary tract symptoms (fever, chills, flank or back pain) [49]
Pyelonephritis	women with positive urine culture and systemic symptoms (fever, chills, flank pain or back pain) [49].

Web Table 4. Recommended Treatments for Parasitic Stool Infections
FMHACA Standard Treatment Guideline,3rd Edition 2014

Intestinal parasite	Recommended treatment, and alternative
Entameoba Histolytica	Metronidazole 500 mg P.O. TID x 5-7 days
Giardia lamblia	Tinidazole 2 gm po single dose<br>Or Alternative:<br>Metronidazole 500 mg po TID x 5 days
**Ascariasis**<br><br>***Ascaris lumbricoids***	Mebendazole, 500mg P.O. once or<br>Albendazole, 400mg P.O. as a single dose<br>Or Alternative:<br>Pyrantel pamoate, 700mg P.O. as a single dose
**Hookworm infestation**<br>***Necator americanus or Ancylostoma duodenale***	Mebendazole, 500mg stat or 100mg P.O. BID for 3 days<br>Albendazole, 400mg P.O. as a single dose<br>Or Alternative:<br>Pyrantel pamoate, 700mg P.O. as a single dose
**Enterobiasis**<br><br>***Enterobius Vermicularis***	Mebendazole, 100mg P.O. BID for 3 days or<br>Or Alternative:<br>Albendazole, 400mg P.O. as a single dose
**Trichuriasis**<br>***T.tricura [Whipworm]***	Mebendazole, 500mg P.O. single dose or,<br>Or Alternative:<br>Albendazole, 400mg, P.O. for three days
**Taeniasis**<br><br>***T.saginata or T.solium***	Praziquantel P.O. 600mg or 10mg/Kg, single dose<br>Or Alternative:<br>Niclosamide, 2g in a single dose P.O.
Hymenolepis nana	Praziquantel, 25mg/kg or 1800mg P.O. single dose<br>Alternative:<br>Niclosamide, 2g P.O. on the first day followed by 1g QD for 6 days
Schistosoma Mansoni	Praziquantel P.O. 1200 mg single dose (or 600 mg po in 2 doses)
**Strongyloidiasis**<br><br>***Strongloidexs stercolaries***	Albendazole 400mg P.O. BID for three consecutive days.

Web Table 5. Biological Specimens Collected in ENAT Study

A table of biospecimens and planned lab testing is presented below. Biospecimens collected for immediate analyses at the APHI laboratory are stored at room temperature using special collection containers and swabs designed to meet this storage requirement. Samples collected for future analyses are aliquoted to appropriate containers and stored at APHI for long term storage in a specially procured freezer for this purpose. All samples are stored for up to 5 years.

ENAT Bio-specimens

			Antenatal		Post-natal	
			≤24 wks	~30-34 wks	0 -<72 hrs	
Biospecimen	N (Total)	Planned lab testing	Enroll	ANCFU2	Birth	1-2 mo.
Maternal urine	800	Culture, subset of 600 for iodine concentration, selenium, environmental toxins	■	■		
Maternal vaginal swab	150	STI/RTI	■			
Maternal blood serum	700	Nutrient levels, TORCH infections	■	■		
blood spot	1000	Inflammation, thyroid, nutrients, miRNAs	■	■		
VAMS + DBS	120	metabolomics, proteomics	■	■	■	■
Maternal vaginal swab	150	Microbiome, metabolome	■	■		■
Maternal stool	120	Microbiome	■	■		■
Maternal oral sample	120	Microbiome	■	■		■
Umbilical cord blood/spot	650	Inflammation, thyroid, nutrients, miRNAs			■	
Infant blood (VAMS + DBS)	120	Metabolomics, Proteomics			■	■
Infant stool	160	Microbiome			■	■
Infant oral sample	100	Microbiome, CMV			■	■
Breast milk	100	Nutrient composition, microbiome				■

Web Form 1. Model Informed Consent Form

Information sheet and Consent: ENP Arm

Name of the organization: Addis Continental Institute of Public Health
Name of the Sponsor: Bill and Melinda Gates Foundation and Brigham and Women's Hospital

Good morning/Good afternoon.
My name is _________________________________. I am a study nurse at Addis Continental Institute of Public Health (ACIPH). ACIPH is doing the ENAT study together with the Federal Ministry of Health, Amhara Regional Health Bureau, Amhara Public Health Institute, and other partners.

Purpose: We are asking you to become part of a research study that has a goal to improve the health of the pregnant women and their babies. The main goal of the study is to see if babies will grow better in the womb if pregnant women are provided with better nutrition and are treated for infections in pregnancy. Pregnant women will be enrolled if they are less than 6 months pregnant and will be followed up to 6 months after the baby's birth. The total number of pregnant women enrolled in this study will be 3,600.

Explanation of study procedures:
We will measure your height, weight, and middle upper arm circumference. If your middle-upper arm circumference is >23 cm, you will be given iodized salt and iron folate to be consumed daily until you deliver your baby. Whenever you come to the health center, a midwife will give nutrition counseling about nutrients in IFA and iodized salt and maintaining appropriate weight gain during pregnancy for you and your baby.

If the study nurse finds that your arm size is small (middle upper arm circumference <23 cm), you will also be provided a nutritious food supplement of a corn soy blend flour. You may use the corn soy blend to make a daily serving to take on each day. These supplements will be given to you at the health center once a month until you give birth. We believe this will give the unborn baby the nutrition s/he needs to grow better.

We also hope that treating infections in pregnancy will help you to have a healthier pregnancy, increase the chances that the baby will deliver at the expected due date, and help the baby grow better in the womb.

If you decide to participate in the study, you will be put into one of two groups. The study team will choose which group you will be put into randomly. There is no way to tell which group you will be in. Neither you, the study staff, nor the health workers can choose what group you will be in. You will have an equal chance of being placed in either of the two group.

If you are assigned to the first group, you will be tested and treated for certain infections. You will be given deworming medication during the second trimester of your pregnancy, and you will be asked to provide a stool sample to screen and treat for persistent helminthiasis (worms) in the

third trimester. You also be asked to give a sample of your urine to test for infection that may affect your health and the health of the baby. The study nurse and laboratory personnel will demonstrate to you how to collect the sample. The urine sample will be analyzed at APHI and you will be asked to come back to the health center within 7 days to get results from your tests.

If you are not in the first group, you will receive the routine antenatal care that is provided at the health center. You will not receive any additional testing or treatment.

After your baby is born, we will need to very carefully measure your baby's height, weight, chest, head and arm size. It is important these measurements happen within three days of giving birth. Even if you give birth at home, a trained person will visit you to carefully take them. We may also measure your baby at a later study visits at 1, 3, and 6 months.

We ask for your permission to be contacted during the study to remind you of study visits, to notify you that your test results have arrived, visit you in case of birth at home and/or to allow us to come to your house to measure the level of iodine in the salt that you use for household consumption. We will remind you about your visit by phone call or text message. If you do not respond, we may visit your home. Please show below how you agree to be contacted:

☐ Text message (to you or a family member with a phone)

☐ Phone call (to you or a family member with a phone)

☐ Home visit (If you do not come to the facility for your visit, a study staff may visit you at your home.)

Participants for these activities are pregnant women, less than 6 months pregnant, who come for ANC at health centers at the study site during the study period. If you are willing to be a part of the study, you need to understand and sign this form. All your personal information for this study will be kept private in our office where no one will be able to see it. Your identity will not be used in any reports or publications that come from the study.

Risk or discomforts: You may feel embarrassed or nervous answering some questions about your health and your baby's health. You do not have to answer every question if you do not want to.

There also maybe risk of aside effect to a medication. If you are treated for an infection, all of the medicines that will be given by your midwife are routine and considered safe to have while pregnant and are not known to hurt the baby.

We will give you our phone number so that you can call us with any concerns about bad reactions to any of the medications. We will help you to be seen and treated at the health center or nearby hospital and help pay the cost of treatment or transportation if it's absolutely necessary.

Benefits: All mothers who join in the study will get an ultrasound and at the third trimester (28 weeks of GA), that will help make sure your pregnancy is healthy. You will be screened for

anemia, get iodized salt that may help you to be more nourished and help your baby grow. If you have poor nutrition, you will get a corn soy blend flour containing nutrients to eat every day. This supplement may also help improve your and your baby's nutrition during the pregnancy.

Some mothers will be tested for infections and get treatment that will help your and your baby's health. Though you may not get money or gifts, being a part of the study is very important in making programs that can help mother and child health, especially in helping babies grow and develop better in the womb.

Confidentiality and Anonymity: Information about you that we will collect by this research project will be kept. Your personal information will not be seen by anyone except the study team, and it will only be used for this research. No identities will be used in any reports or publications resulting from the study.

Right to Refuse or Withdraw: Being a part of this study and everything you are asked to do is completely voluntary. You have the full right to refuse from being a part of this study. You also have the full right to stop being a part of this study at any time you wish to. Refusing to be in the study or stopping will not affect the services that you get from the health facility.

We thank you very much for your time. If you have any questions, you can call:
Professor Yemane Berhane: Addis Continental Institute of Public Health,
Tel: +251 114 168207/ + 251 911219785 (mobile)
E-mail: yemaneberhane@addiscontinentual.edu.et OR yemaneberhane@gmail.com

Addis Continental Institute of Public Health, Tel: +251 116 390039
E-mail: aciphaddis@gmail.com

Consent Form

I have read this information and decided to be a part of this study. The general purpose of study has been explained well to me and I was given the chance to ask questions. I understand that I can stop my participation and anything for the study at any time.

______________________ _______________ ________________
Participants name Participants age ENAT Study ID

_______________________ _______________
Participants signature Date (DD/MM/YY)

______________________ _______________ ________________
Study nurse name Study nurse signature Date (DD/MM/YY)

If illiterate:

I have seen the careful reading of the consent form to the potential participant and the participant has been able to ask questions. I agree that the participant has given consent freely.

______________________ _______________ ________________
Witness name Witness signature Date (DD/MM/YY)

______________________ _______________ ________________
Participants name Participants age ENAT Study ID

Thumb print of participant Date (DD/MM/YY)

______________________ _______________ ________________
Study nurse name Study nurse signature Date (DD/MM/YY)
