# Supplementary material for: Enhancing Nutrition and Antenatal Infection Treatment (ENAT) study: protocol of a pragmatic clinical effectiveness study to improve birth outcomes in Ethiopia
Source: BMJ Paediatr Open. 2022 Jan 13;6(1):e001327. doi: 10.1136/bmjpo-2021-001327 (PMC8762145; doi:10.1136/bmjpo-2021-001327)
Supplement: Author's manuscript [file bmjpo-2021-001327.draft_revisions.pdf]

BMJ Paediatrics Open

BMJ Paediatrics Open is committed to open peer review. As part of this commitment we make the peer review history of every article we publish publicly available.

When an article is published we post the peer reviewers' comments and the authors' responses online. We also post the versions of the paper that were used during peer review. These are the versions that the peer review comments apply to.

The versions of the paper that follow are the versions that were submitted during the peer review process. They are not the versions of record or the final published versions. They should not be cited or distributed as the published version of this manuscript.

BMJ Paediatrics Open is an open access journal and the full, final, typeset and author-corrected version of record of the manuscript is available on our site with no access controls, subscription charges or pay-per-view fees (http://bmjpaedsopen.bmj.com).

If you have any questions on BMJ Paediatrics Open's open peer review process please email
info.bmjpo@bmj.com

BMJ Paediatrics Open

The Enhancing Nutrition and Antenatal Infection Treatment (ENAT) Study: Protocol of a pragmatic clinical effectiveness study to improve birth outcomes in Ethiopia

Journal:	*BMJ Paediatrics Open*
Manuscript ID	bmjpo-2021-001327
Article Type:	Protocol
Date Submitted by the Author:	21-Oct-2021
Complete List of Authors:	Lee, Anne; Brigham and Women's Hospital, Pediatric Newborn Medicine; Harvard Medical School<br>Abate, Firehiwot Workneh; Addis Continental Institute of Public Health<br>Mullany, Luke C.; Johns Hopkins Bloomberg School of Public Health, Department of International Health<br>Baye, Estifanos; Brigham and Women's Hospital, Department of Pediatric Newborn Medicine<br>Berhane, Yoseph Yemane; Addis Continental Institute of Public Health<br>Derebe, Mulatu Melese; Amhara Public Health Institute<br>Eglovitch, Michelle; Brigham and Women's Hospital, Department of Pediatric Newborn Medicine<br>Fasil, Nebiyou; Addis Continental Institute of Public Health<br>Olson, Ingrid; Brigham and Women's Hospital, Department of Pediatric Newborn Medicine<br>Kidane, Workagegnehu Tarekegn; Addis Continental Institute of Public Health<br>Shiferw, Tigest; Addis Continental Institute of Public Health<br>Shiferie, Fisseha; Addis Continental Institute of Public Health<br>Tsegaye, Fitsum; Addis Continental Institute of Public Health<br>Tsegaye, Sitota; Addis Continental Institute of Public Health<br>Yibeltal, Kalkidan; Addis Continental Institute of Public Health<br>Chan, Grace; Boston Children's Hospital, Harvard Medical School, Department of Medical Critical Care; Harvard TH Chan School of Public Health, Department of Epidemiology<br>Christian, Parul; Johns Hopkins Bloomberg School of Public Health, Department of International Health<br>Isanaka, Sheila; Harvard TH Chan School of Public Health, Departments of Nutrition and Global Health and Population<br>Kang, Yunhee; Johns Hopkins Bloomberg School of Public Health, Department of International Health<br>Lu, Chunling; Brigham and Women's Hospital, Division of Global Health Equity; Harvard Medical School, Department of Global Health and Social Medicine<br>Molina, Rose ; Beth Israel Deaconess Medical Center<br>Stojanov, Michele; Harvard Medical School, Department of Obstetrics and Gynecology<br>Van Dyk, Fred; Johns Hopkins Bloomberg School of Public Health, Department of International Health

Tadesse, Amare; Addis Continental Institute of Public Health; London School of Hygiene and Tropical Medicine, Department of Infectious Disease Epidemiology
Wylie, Blair; Harvard Medical School; Beth Israel Deaconess Medical Center, Department of Obstetrics and Gynecology
Worku, Alemayehu; Addis Continental Institute of Public Health
Berhane, Yemane; Addis Continental Institute of Public Health

Keywords: Neonatology, Growth

SCHOLARONE™
Manuscripts

I, the Submitting Author has the right to grant and does grant on behalf of all authors of the Work (as defined in the below author licence), an exclusive licence and/or a non-exclusive licence for contributions from authors who are: i) UK Crown employees; ii) where BMJ has agreed a CC-BY licence shall apply, and/or iii) in accordance with the terms applicable for US Federal Government officers or employees acting as part of their official duties; on a worldwide, perpetual, irrevocable, royalty-free basis to BMJ Publishing Group Ltd ("BMJ") its licensees and where the relevant Journal is co-owned by BMJ to the co-owners of the Journal, to publish the Work in this journal and any other BMJ products and to exploit all rights, as set out in our licence.

The Submitting Author accepts and understands that any supply made under these terms is made by BMJ to the Submitting Author unless you are acting as an employee on behalf of your employer or a postgraduate student of an affiliated institution which is paying any applicable article publishing charge ("APC") for Open Access articles. Where the Submitting Author wishes to make the Work available on an Open Access basis (and intends to pay the relevant APC), the terms of reuse of such Open Access shall be governed by a Creative Commons licence – details of these licences and which Creative Commons licence will apply to this Work are set out in our licence referred to above.

Other than as permitted in any relevant BMJ Author's Self Archiving Policies, I confirm this Work has not been accepted for publication elsewhere, is not being considered for publication elsewhere and does not duplicate material already published. I confirm all authors consent to publication of this Work and authorise the granting of this licence.

Confidential: For Review Only

The Enhancing Nutrition and Antenatal Infection Treatment (ENAT) Study: Protocol of a pragmatic clinical effectiveness study to improve birth outcomes in Ethiopia

Anne CC Lee[1,2*], Firehiwot Workneh Abate[3], Luke C. Mullany[4], Estifanos Baye[1], Yoseph Yemane Berhane[3], Mulatu Melese Derebe[5], Michelle Eglovitch[1], Nebiyou Fasil[3], Ingrid E Olson[1], Workagegnehu Tarekegn Kidane[3], Tigest Shiferw[3], Fisseha Shiferie[3], Fitsum Tsegaye[3], Sitota Tsegaye[3], Kalkidan Yibeltal[3], Grace J. Chan[2,6,7], Parul Christian[4], Sheila Isanaka[8], Yunhee Kang[4], Chunling Lu[2,9], Rose L. Molina[2,10], Michele D. Stojanov[10], Fred Van Dyk[4], Amare Worku Tadesse[3,11], Blair J. Wylie[2,9], Alemayehu Worku[3], Yemane Berhane[3]

[1]Department of Pediatric Newborn Medicine, Brigham and Women's Hospital, Boston, MA, USA
[2]Harvard Medical School, Boston, Massachusetts, USA
[3]Addis Continental Institute of Public Health, Addis Ababa, Ethiopia
[4] Department of International Health, Johns Hopkins Bloomberg School of Public Health, Baltimore, MD, USA.
[5]Amhara Public Health Institute, Bahir Dar, Ethiopia
[6]Department of Epidemiology, Harvard T.H. Chan School of Public Health, Boston, MA, USA
[7] Department of Medical Critical Care, Boston Children's Hospital, Harvard Medical School, Boston, Massachusetts, USA
[8]Departments of Nutrition and Global Health and Population, Harvard T.H. Chan School of Public Health, Boston, MA, USA
[9] Division of Global Health Equity, Brigham and Women's Hospital, Boston, Massachusetts, USA
[10]Department of Obstetrics and Gynecology, Beth Israel Deaconess Medical Center, Harvard Medical School, Boston, MA, USA
[11]Department of Infectious Disease Epidemiology, London School of Hygiene and Tropical Medicine, London, UK

Corresponding Author:
Anne CC Lee
75 Francis St, Boston MA 012115
617-732-8343
Alee6@bwh.harvard.edu

Word Count: 4264

Confidential: For Review Only

ABSTRACT

Introduction: The WHO Nutrition Target aims to reduce the global prevalence of low birth weight 30% by 2025. The Enhancing Nutrition and Antenatal Infection Treatment (ENAT) study will test the impact of packages of pregnancy interventions to enhance maternal nutrition and infection management on birth outcomes in rural Ethiopia.

Methods and analysis: ENAT is a pragmatic, open-label, 2x2 factorial, randomised clinical effectiveness study implemented in 12 rural health centers in Amhara, Ethiopia. Eligible pregnant women presenting at antenatal care (ANC) visits at <24 weeks gestation are enrolled (n=2400). ANC quality is strengthened across all centers. Health centers are randomised to receive an enhanced nutrition package (ENP) or standard nutrition care, and within each health center, individual women are randomised to receive an enhanced infection management package (EIMP) or standard infection care. At ENP centers, women receive a regular supply of adequately iodized salt and iron-folate (IFA), enhanced nutrition counseling, and those with mid-upper arm circumference <23 cm receive a micronutrient fortified balanced energy protein supplement (corn soya blend) until delivery. In standard nutrition centers, women receive routine counseling and IFA. EIMP women have additional screening/treatment for urinary and sexual/reproductive tract infections and intensive deworming. Non-EIMP women are managed syndromically per Ministry of Health Guidelines. Participants are followed until 1 month postpartum, and a subset until 6 months. The primary study outcomes are newborn weight and length measured at <72 hours of age. Secondary outcomes include preterm birth, low birthweight, and stillbirth rates; newborn head circumference; infant weight and length for age z-scores at birth; maternal anemia; and weight gain during pregnancy.

Ethics and dissemination: ENAT is approved by the Institutional Review Boards of Addis Continental Institute of Public Health (001-A1-2019) and Mass General Brigham (2018P002479). Results will be disseminated to local and international stakeholders.

ISRCTN Registration: 15116516

Funding: Bill & Melinda Gates Foundation

Protocol: Version 4.0 (August 2021)

Confidential: For Review Only

Article Summary

What is known about the subject:

- In low- and middle-income countries, maternal undernutrition is prevalent and a major risk factor for adverse birth outcomes, including spontaneous preterm birth, low birth weight and small for gestational age infants.
- Maternal infections in pregnancy are also common, yet under-recognized risk factors for preterm birth and poor fetal growth in low- and middle-income countries.
- Beyond their independent effects, maternal infections and nutritional status may have synergistic effects on fetal growth and gestational length.

What this study hopes to add:

- The ENAT study will increase the evidence-base on the role of antenatal infection management and targeted, fortified balanced energy protein supplementation on maternal and birth outcomes.
- ENAT intervention packages were co-designed with local stakeholders and are implemented within existing health systems; this approach will maximize potential uptake, scalability, impact and cost-effectiveness.
- The factorial study design allows testing of the individual and combined effects of nutrition and infection management packages on birth outcomes.

Confidential: For Review Only

INTRODUCTION

The World Health Organization (WHO) Third Global Nutrition Target aims to reduce the proportion of babies born low birthweight (LBW, <2500 grams) by 30% by the year 2025[1]. Approximately 20.5 million infants were born LBW in 2015, with 91% from low-middle income countries (LMIC) and 24% in Sub-Saharan Africa [2]. The main etiologies of low birthweight are preterm birth (<37 gestational weeks) and fetal growth restriction, commonly classified as small-for-gestational-age (SGA) at birth. Preterm and SGA infants carry increased risk of mortality, morbidity, childhood stunting, neurodevelopmental impairment, and adult chronic disease [3–7]. Prevention of preterm birth and SGA is a key public health strategy to improve child survival and health in LMICs.

In LMICs, maternal undernutrition is prevalent and a major risk factor for adverse birth outcomes, including spontaneous preterm birth, LBW and SGA [8,9]. Interventions to improve maternal nutritional status in pregnancy, including iron-folate [10], multiple-micronutrients [11], and balanced protein-energy (BEP) supplementation [12,13], have been individually tested and found to be effective in increasing mean birthweight. However, lower than expected benefit has been observed with the individual nutritional interventions [10,12–14]. Greater effect sizes are noted in undernourished women, and there is a need for additional studies with standardized supplementation criteria as well as the combination of BEP and micronutrient supplementation [12,13].

Maternal infections in pregnancy are also common, yet under-recognized risk factors for preterm birth and poor fetal growth in LMICs. Urinary tract infection or asymptomatic bacteriuria may affect 9-80% of pregnancies in sub-Saharan Africa [15] and are associated with a 2-fold elevated risk of preterm delivery [16]. Helminthic infections are prevalent and associated with systemic inflammation, LBW and preterm birth [17–19]. Genital tract infections may ascend the reproductive tract and lead to infection and inflammation in the amniotic fluid, predisposing to preterm birth [20]. In LMICs, screening and treatment of genitourinary tract infections during routine antenatal care (ANC) is infrequent due to lack of resources and capacity for laboratory testing. While epidemiologic data has consistently established associations between prenatal infections and adverse pregnancy outcomes, there is limited evidence on the effectiveness of prenatal interventions to screen and treat infections to prevent LBW in LMICs.

https://mc.manuscriptcentral.com/bmjpo
Confidential: For Review Only

Beyond their independent effects, maternal infections and nutritional status may have synergistic effects on fetal growth and gestational length [21–25]. Figure 1 depicts several pathways linking maternal nutrition and infections in pregnancy. This framework provides the basis for the hypothesis that targeting both risk factors in pregnancy may lead to more substantial, and potentially synergistic, improvements in fetal growth and pregnancy length [26].

This manuscript details the study protocol for the Enhancing Nutrition and Antenatal Infection Treatment (ENAT) study in Amhara region, Ethiopia. In 2016, WHO released recommendations on an evidence-based, core package of ANC to optimize the pregnancy experience and outcomes [27], include guidelines on nutrition and infection management in pregnancy (Web Table 1). In Ethiopia, not all recommendations have been adopted or achieved high level of coverage or quality in ANC. The primary aim of the ENAT study is to determine the impact of WHO-recommended ANC interventions to optimize maternal nutrition and manage maternal pregnancy infections on infant birth size in Amhara, Ethiopia. We hypothesize that the nutrition and infection management packages will independently increase newborn birth weight and length, and that the combined effect of both packages will be greater than either alone.

METHODS AND ANALYSIS

Study Design

The ENAT study is a 2x2 factorial pragmatic, open-label, randomised clinical effectiveness study with cluster randomisation of the enhanced nutrition package (ENP) versus standard care (non-ENP), and individual level randomisation of an enhanced infection management package (EIMP) versus standard care (non-EIMP) (Figure 2).

Study Setting

The ENAT study site was established in 2018 as a partnership with the Addis Continental Institute of Public Health (ACIPH), Amhara Regional Health Bureau, Amhara Public Health Institute and Brigham and Women's Hospital. The Amhara region has low-resourced health systems and poor health indicators. As per the 2016 Ethiopian Demographic Health Services (EDHS) data, Amhara had the country's highest rates of neonatal mortality (47 per 1000 live births) and LBW (22.2%), and high maternal mortality rate (412 per 100,000 live births) [28].

https://mc.manuscriptcentral.com/bmjpo
Confidential: For Review Only

Rates of any prenatal care and institutional delivery and were 82.6% and 54.2%, respectively [29]. One in four women of reproductive age are underweight (body mass index<18.5 kg/m^2) [28] and geohelminth infections are prevalent, ranging from 21.1-43.5% [30,31].

The ENAT study is conducted in 12 rural health centers (each serving ~25,000 population) in West Gojjam (South and North Achefer districts [woredas]) and South Gondar (Dera and Libokemem districts) zones, Amhara (Figure 3, Site Map). The districts were chosen in collaboration with the Amhara Regional Health Bureau based on the high rates of undernutrition, risk of low birth weight, need for nutritional programs, and proximity to the regional laboratory. The study health centers were chosen based on accessibility, total ANC volume (minimum 250 women presenting to ANC/year), and infrastructure (functional laboratory).

Patient and public involvement

Prior to the study, formative work was conducted with a range of community members (mothers, families, community and religious leaders, health providers) [32]. This feedback directly informed the design of the study interventions, packages, and their implementation. Community sensitization was performed prior to initiating the study.

Study participants and recruitment

Pregnant women are recruited from ANC visits in designated ENAT study health centers. A study nurse explains study procedures and obtains written informed consent. For illiterate women, an impartial witness attests to consent and the woman provides a thumbprint.

To encourage early presentation at ANC clinics, community sensitization was conducted prior to study initiation. Study field data collectors and community cadres disseminated information about the ENAT study at monthly community-based pregnant women's conferences, and community and religious gatherings and encouraged presentation to the health centers if/as soon as pregnancy was suspected.

Inclusion criteria:

- ≤24 weeks gestation based on a clinical algorithm (LMP and/or symphysis fundal height) who have a viable pregnancy

Exclusion criteria:

- Pregnant women presenting for first ANC >24 weeks

https://mc.manuscriptcentral.com/bmjpo
Confidential: For Review Only

- Pregnant women who live >2 hours walking distance from ENAT health centers
- Pregnant women presenting at first ANC with fetus that is non-viable (without a heartbeat on enrollment ultrasound)

ENAT Study Interventions

Health systems strengthening of ANC services was performed in ENAT health centers prior to the study to benefit all women receiving care in these facilities. Health systems strengthening was conducted in partnership with Amhara Regional Health Bureau and local partners including Jhpiego. Staff were trained in ANC standards, guidelines, and measurements (blood pressure, gestational age, birth weight [33]). Facilities were stocked with basic equipment, medications, and diagnostic testing. Laboratory capacity was augmented and all health centers were equipped with ultrasound machines.

A. Enhanced Nutrition Package (ENP) (Table 1)

Table 1: Enhanced Nutrition Package Components

ENP Content	Activity	Frequency
Nutritional education/counselling	Counselling about healthy eating, adequate pregnancy weight gain, increasing protein and energy in diet, importance of Iron/folate, iodized salt	Every ANC visit (monthly, potentially less frequently pending COVID)
Iron-Folic Acid	Strengthen counseling, supply and reinforcement of daily IFA (60 mg Fe/400 µg folic acid)	Provide 90-day supply per FMOH, adherence/counseling at every ANC visit
Iodized salt	For all pregnant women, provide up to 1kg at enrollment and follow up visit for household use during pregnancy with counselling	Provided adequately iodized salt at enrollment, and monthly follow up visits; adherence/counseling every ANC visit
Balanced Energy Protein supplement	For pregnant women with MUAC <23 cm, local Corn Soya Blend 200 gm daily supplement (784 kcal/day, 28 g protein)	Daily supplement, distributed at ANC visits (monthly, given COVID may provide longer term supply)

A.1 Nutritional Education/Counseling on Adequate Pregnancy Nutrition and Weight Gain: Routine ANC nutrition counseling includes increasing intake and dietary diversity, however can vary depending on patient load. For ENP centers, supplementary, locally-contextualized, counseling was developed based on our formative work[32], and is delivered by midwives. Content includes BMI-based recommended weight gain, dietary diversity, and educational messages developed to address local cultural beliefs related to dietary intake during pregnancy as well as side effects of iron-folate. Nutrition education materials, including posters and videos,

Confidential: For Review Only

are shown in ENP health centers to promote women's behavior change and maximize their exposure to various but consistent nutrition messages.

A.2 Iron Folic Acid (IFA): The Ethiopian FMOH recommends 60 mg iron plus 400 µg folic acid supplements, orally once daily in pregnancy. In August-Sept 2019, coverage of IFA was 46.3% in the ENAT health centers, and our formative work indicated that barriers included local beliefs about "big babies" and side effects such as constipation. In the ENP health centers, additional counseling is conducted, utilizing video/media to address common cultural beliefs and side effects, and women are reminded at each ANC visit about IFA consumption, management of side effects and provided refills per FMOH guidelines.

A.3 Provision of Adequately Iodized Household Salt: In ENP centers, we provide a monthly household supply of high-quality adequately iodized salt at every ANC visit. The iodized salt (Waff Manufacturing, 30-40 ppm potassium iodate, 600 gm bottle) is packaged in airtight, resealable, polyethylene containers, to allow resealing after use and to reduce risk of evaporative losses at the household level. Quality control procedures are in place to ensure that iodization is in the proper range at production and maintained at distribution sites. Women are counselled that salt should be used to replace their household salt, on the approximate daily use (~10 gm (3 pinches)/day), the proper storage of salt (away from light, heat, humidity, re-capping container after use) and use of salt only after cooking/heating of food.

A.4 Fortified Balanced Energy Protein (BEP) Supplement for Malnourished Women (MUAC <23 cm): In the ENP health centers, women who have MUAC <23 cm at enrollment, or at any follow up ANC visit, are provided with a monthly supply of locally produced micronutrient fortified corn soya flour blend (Super Cereal, Faffa Food Share Company, Addis Ababa, Ethiopia) at every ANC visit until delivery. The daily corn soya blend (CSB) supplement (200gm) is provided in addition to normal meals and contains 28 g of protein and 784 kcal (Web Table 2). This protein composition falls within recommendations that a BEP supplement provide ~50% of the additional protein requirement in the 3rd trimester (range of 28-36g for malnourished populations) [34]. Micronutrient composition of the provided BEP is also shown in Web Table 2. The fortified BEP supplement meets the IOM recommended levels in pregnancy [35] for Vitamins A, D, E, B_2, B_3, B_6, B_{12}, C, calcium and phosphorus (see Web Table 2). 35 sachets (200 gm each) are distributed to women at enrollment and at follow up monthly ANC visits to

Confidential: For Review Only

allow for additional doses in case she is delayed in returning for ANC and/or for potential family sharing practices.

B. Enhanced Infection Management Package (EIMP) (Table 2)

Women randomised to the EIMP intervention, receive the following interventions at their first ANC visit >12 weeks.

B.1 Urinary Tract Infection/Asymptomatic Bacteriuria: A clean catch midstream urine specimen is collected using a vacutainer with boric acid preservative (Beckton Dickinson). Urine culture and antibiotic susceptibility testing are performed at Amhara Public Health Institute (APHI), the regional laboratory certified by the ENAO (Ethiopian National Accreditation Office) - ISO (International Organization for Standardization) 15189. Antibiotic susceptibility is determined including the Vitek method (bioMerieux, Marcy l'Etoile, France), or Kirby Bauer Disk Diffusion. Urinary tract infections (UTI) are classified in Web Table 3 and treated with an oral antibiotic based on antibiotic sensitivity patterns (Web Table 4). Antibiotics are provided to pregnant women at no cost and the first dose is directly observed. Women with severe illness or difficult to treat infections are referred to the obstetrics department at Feligot Hiwot hospital. Women provide a test of cure specimens at the following ANC visit.

Table 2. ENAT EIMP Components

**Infection**	**ENAT Enhanced Infection Management Package (EIMP) Activity**
Urinary Tract Infection/Asymptomatic Bacteriuria	*Screen:* Urine culture and antibiotic sensitivity.<br><br>*Treat:* Initially per clinical protocol, with targeted antibiotic treatment based on antibiotic resistance patterns.
Sexually Transmitted/Reproductive Tract Infections	*Screen:*<br>1) **ALL pregnant women** for gonorrhea, chlamydia using accurate rapid diagnostic testing for chlamydia and gonorrhea<br><br>2) Pregnant women ***with symptoms*** are screened for trichomonas and bacterial vaginosis<br><br>*Treat:* All positive cases and partners (for gonorrhea, chlamydia, trichomonas)
Geo-helminths	Presumptive deworming with mebendazole 500 mg or albendazole 400 mg as per FMOH guideline; Stool screen and treatment for parasitic infections at least 4 weeks after deworming. If positive, treatment for intestinal parasites per FMOH guidelines.

https://mc.manuscriptcentral.com/bmjpo
Confidential: For Review Only

B.2 Sexually Transmitted/Reproductive Tract Infections: Women self-collect vaginal specimens that are tested for gonorrhea and chlamydia using the *Xpert® CT/NG assay* (*Cepheid*, Sunnyvale, CA), a modular cartridge-based platform for testing each specimen by nucleic acid amplification at APHI. Chlamydia is treated with Azithromycin 1 gram orally once and gonorrhea is treated with Ceftriaxone 250 mg IM once + Azithromycin 1gram orally once. Partner treatment is on a voluntary basis with a regimen as recommended by the Ethiopian STI management guidelines [36]. A test of cure is obtained at the next ANC visit.

For women who report symptoms of abnormal vaginal discharge, vulvar symptoms, or lower abdominal tenderness, additional vaginal swabs are collected for trichomonas and bacterial vaginosis by point of care diagnostics. Trichomonas is tested using the OSOM® trichomonas rapid test (Sekisui Diagnostics, Lexington, MA). Bacterial vaginosis is tested using Diagnosit® BVBLUE® test (Gryphus Diagnostics, Knoxville, TN)). Trichomonas is treated with Metronidazole 2 grams orally once, and partners are treated. Bacterial vaginosis is treated with metronidazole 500 mg twice daily for 7 days.

B.3. Parasitic Intestinal Infections: In settings of high geo-helminth burden, WHO recommends prophylactic deworming in 2nd and 3rd trimester of pregnancy [37]. At study initiation, mebendazole (500 mg) was provided twice in pregnancy consistent with WHO guidelines. Due to health provider concerns regarding medication package insert information contraindicating use in early pregnancy, in September 2020, the protocol was modified to a single presumptive deworming in the 3rd trimester. With the adoption of new Ethiopian FMOH ANC guidelines allowing earlier provision of anti-helminthics in pregnancy, in May 2021, the ENAT protocol was modified to provide presumptive deworming in the 2nd trimester followed by a stool screening and treatment at least 4 weeks later. In the first post-deworming visit, stool is screened for intestinal parasites in the health center lab using wet mount microscopy available at the health center. Women identified with parasitic infections are treated as per FMOH recommendations (Web Table 4).

Randomisation/Allocation

At the first level of randomisation, clusters (i.e., health centers) are randomised into one of two nutrition interventions: a) ENP or b) standard nutrition care. We performed a constrained randomisation to ensure balance across the two arms of the study for key

Confidential: For Review Only

indicators including: population size, pre-study ANC coverage rates, number of births, and travel time to the regional center of Bahir Dar. We: 1) set reasonable tolerance levels for the restriction variables, 2) created all possible random sequences, where each sequence allocated 6 health centers to the ENAT Nutrition Package and 6 health centers to routine care, 3) assessed each sequence as to whether or not it met these restriction criteria, and 4) choose randomly from the subset of all such allocation sequences that met the criteria. At the second level of randomisation, we randomised individual pregnant women presenting for ANC at each health center to receive a) *ENAT Enhanced Infection Management Package (EIMP)*, or b) standard infection care (Figure 2). Each health center received a pre-generated randomisation list of sequential individual assignments to EIMP or standard care, where assignments were equally allocated to each arm within randomly permuted blocks of size 4, 8, or 12. The randomisation lists were generated separately, by health center, using a script written by one of the authors (LCM) in R [38].

Outcome Measures

The **primary outcomes** are

P1. Newborn weight measured within 72 hours of birth
P2. Newborn length measured within 72 hours of birth

The **secondary outcomes** include:

S1. Length of gestation, with gestational age determined by <24-week pregnancy ultrasonography
S2. Proportion of pregnancies resulting in spontaneous preterm delivery
S3. Proportion of newborns born of low birthweight (<2500 grams), as measured within 72 hours of life
S4. Proportion of newborns born small-for-gestational age, as defined by the INTERGROWTH 21st neonatal birth weight standard.
S5. Stillbirth rate
S6. Newborn head circumference within 72 hours of birth
S7. Infant Z-scores for weight-for-age, length-for-age, head circumference-for-age within 72 hours of birth
S8. Maternal gestational weight gain
S9. Maternal anemia (3rd trimester)

The definitions used for each outcome measure is shown in Table 3.

https://mc.manuscriptcentral.com/bmjpo
Confidential: For Review Only

Table 3: ENAT Study Outcomes

**Primary outcomes**	
**P1. Newborn weight <72 hours of birth**	Weight of the unclothed infant measured at <72 hours of life
**P2. Newborn length measured at birth**	Infant Length measured at <72 hours of life
**Secondary outcomes**	
**S1. Gestational age**	Gestational age determined by enrollment ultrasound, CRL used if <95 mm (Intergrowth 21st), then BPD/FL (WHO Kiserud) used if CRL>95 mm or missing
**S2a. Proportion of pregnancies resulting in preterm delivery**	This measure is defined as the spontaneous termination of pregnancy from 24 to <37 weeks resulting in: 1) preterm live birth, 2) fetal loss (spontaneous pregnancy loss 24 to <37 weeks) that is not due to induced abortion.<br><br>Numerator<br>• Live births (28-<37weeks)<br>• Stillbirth (28-<37 weeks)<br>• Late Miscarriage (24-<28 weeks)<br>Denominator<br>All birth outcomes ≥24 weeks
**S2b. Preterm birth**	Numerator: Live births <37 weeks of gestation<br>Denominator: Livebirths
**S3. Small for Gestational Age[a] (Intergrowth)**	<10% birthweight for GA by sex compared to Intergrowth reference[2]
**S4. Low birthweight rate**	Low birthweight is defined as birthweight (measured within the first 72 h of life) of <2500 g. We will also assess the outcome of birthweight <2000 g.
**S5. Stillbirth rate**	Numerator: Stillbirth (≥28 weeks gestation)- fetal death with no signs of life<br>*A preterm stillbirth is defined as an infant born without signs of life (no spontaneous crying, breathing, and/or movement) at 28 to <37 weeks gestation.*<br>*A term stillbirth is defined as an infant born without signs of life at ≥37 wks*<br><br>Denominator: All live births and stillbirths ≥28 weeks
**S6. Newborn head circumference**	Head circumference of the infant measured at <72 hours of age
**S7. Newborn weight, length, and head circumference for age Z-scores**	Infant weight, length, and head circumference for age z-scores measured at <72 hours of life, calculated using the Intergrowth reference for size at birth.
**S8. Rate of weight gain in pregnancy**	Maternal weight gain (kg) per week gestation in the 2nd and 3rd trimester
**S9. Maternal anemia**	Mean hemoglobin concentration in 3rd trimester<br>(Mission hemoglobinometer)

[a]Abbreviations: WHO, World Health Organization; UTI, urinary tract infection; ANC, antenatal care; SGA, small-for-gestational-age; GA, gestational age

Confidential: For Review Only

Data Collection

The timeline of individual participant study visits, measurements and data collection are shown in Table 4. All study visits are conducted at the health center, with the exception of the birth visit that may be conducted at home within 72 hours of delivery, for births occurring at home or outside of the study area. Adherence monitoring visits also occur at the home for those participants who do not return to the health center for follow up.

The core of the data collection system is the Survey Solutions platform (World Bank, v20.08, 2021). Study nurses enter data directly into electronic tablets with programmed validity checks during study visits. Paper forms are used if tablets are temporarily unavailable. The tablets are regularly synchronized to the server on the ACIPH campus. A web-based dashboard supports data collectors, supervisors, and investigators in real time management and monitoring of study activities.

Confidential: For Review Only

Table 4. Participant timeline schedule of enrolment, interventions, assessment, and visits

	STUDY PERIOD						
		Post-allocation					
	Allocation	Prenatal				Postnatal	
**TIMEPOINT**	**Enrolment <24 weeks**	***ANC1***	***ANC2***	***ANC3***	***etc.***	***Birth***	***1 month***
**ENROLMENT:**							
**Eligibility screen**	X						
**Informed consent**	X						
**Allocation**	X						
**INTERVENTIONS:**							
**ENP**	X	X	X	X	X		
**EIMP**	X	X	X	X	X		
**ENP + EIMP**	X	X	X	X	X		
**ASSESSMENTS:**							
MOTHERS							
US (fetal growth & GA determination)	X			X			
Basic medical & obstetric history	X						
Socioeconomic status	X						
Health care costs	X		X				
Food insecurity and Dietary Intake	X	X		X			
Maternal stress and depression		X		X			X
Maternal anthropometrics	X	X	X	X	etc.	X	X
Maternal morbidity	X	X	X	X	etc.	X	X
Labor and Delivery characteristics						X	
Assessment of home environment						X	
INFANTS							
Anthropometrics						X	X
Breast feeding practices						X	X
Morbidity and mortality						X	X

Confidential: For Review Only

Enrollment Visit

At the enrollment visit at the health center, data is collected on the participant's socio-economic status, basic medical and obstetric history, pregnancy history, maternal morbidity including COVID-19, food security, and dietary intake. A dietary quality questionnaire is administered, which has been used in the Ethiopian context [39].

A basic abdominal obstetric ultrasound is performed by a trained research nurse at the enrollment visit for pregnancy dating. An intensive ultrasonography training and standardization was performed by General Electric, Ethiopian Radiography Association and sonographers from Beth Israel Deaconess Hospital (Boston, MA). Sonographers measure crown-rump length, bi-parietal diameter (outer to inner), head circumference, femoral length, abdominal circumference in duplicate. Approximately 10% of images are externally reviewed (MS, BJW) for quality control.

Maternal and infant anthropometrics are measured by research staff (nurses, data collectors) at baseline and follow up visits (Table 3). Maternal weight is measured with a digital scale (ADE M317600, Germany; precision 100 gm) and height is measured using an adult stadiometer (Shorr Productions HeightLite). All measurements are performed twice, with a third measurement done if the difference is greater than the minimal acceptable difference defined by Intergrowth 21 [40]. Nurses and data collectors are trained and standardized in anthropometric measurements at the start of the study and every 6 months.

Follow up ANC Visits

During the follow up ANC visits at the health center, research staff interview women about their health status, morbidity, pregnancy history/complications, counselling/services received, maternal mental health screen, and dietary intake. Data is abstracted from routine ANC records, including blood pressure, lab testing results, and management. Maternal weight and mid-upper arm circumference are measured. In a subset of women, a semi-quantitative food frequency questionnaire for ~70 food items is administered at ANC visits. Hemoglobin is measured during enrollment in the 1st or 2nd and 3rd trimester ANC visits (Hemocue 301c). Venous hemoglobin is measured if blood is already being drawn for other purposes, and otherwise capillary hemoglobin is measured.

Confidential: For Review Only

Adherence to each nutritional supplement is assessed at every ANC visit. The participant is asked to bring the used IFA bottle, salt container, and BEP sachets back to the health center at each ANC visit. Pill or empty sachet count is done, and the salt container weighed. The participant is also asked to recall the number of sachets and/or pills that were taken in the last 7 days, and since the last visit. For mothers who do not attend scheduled ANC visits, a home visit is made by a data collector to assess adherence and conduct pill/sachet counts and remind the mother to return for ANC and study visits.

A repeat ultrasound is performed in the 3rd trimester to monitor fetal growth and assess the position of the baby. If the fetus is determined to be in non-vertex positioning at the 3rd trimester scan, the nurse recommends that the women deliver in the nearest hospital with Cesarean section capacity.

Birth Visit

Participants who deliver in health facilities are assessed by research staff based in health centers or hospitals as soon as possible after birth, but within 72 hours of life. Data is gathered from women and from chart review about the delivery and immediate postpartum period. Medical records are reviewed for intrapartum course (e.g., vital signs, duration of labor), delivery complications, and maternal/neonatal morbidity. For deliveries that occur at home, a home visit is made by research staff as soon as possible upon birth notification (within 72 hours). Maternal history is obtained per self-report regarding delivery history/complications, and postpartum maternal/neonatal morbidity.

Infant weight is measured using a high quality, precise digital infant scale (ADE M112600, Germany; precision 5 gm). Infant length is measured using a portable infantometer (Perspective Enterprises PE-RILB-LTWT, Michigan USA, precision 1 mm). Recumbent length is recorded to the last completed (not the nearest) mm. Head, chest, and mid upper arm circumferences (MUAC) is measured to the nearest millimeter (mm) using insertion tapes (Shorr productions, Maryland USA). Daily calibration checks are made before each use of infant weighing scales, and length boards to ensure accuracy of measurement [41,42].

Postnatal Visit

Confidential: For Review Only

Postnatal visits are made at 4-6 weeks for all participants to collect data on maternal and infant vital status, health, morbidity and anthropometrics. Visits are conducted primarily at the health center, and home visits are made for those who do not return for follow up. For infants who follow up at the health center for routine postnatal care or immunizations, study visits are additionally conducted at 3 and 6 months.

Cost Data

Data regarding the costs of delivery of the ENAT study interventions is collected in all study arms. Costs of interventions include three components: system-level costs, costs incurred by health workers for participating in the interventions, and costs incurred by individual patients and families. At the system level, costs are captured using the World Health Organization (WHO) framework [43] using modified survey tools based on our published survey instruments, cost estimation protocols, and procedures that have been validated and used in other LMICs (e.g., Rwanda, India) [44–46]; Costs incurred by health workers for receiving training include time or money spent for participating in training sessions. For all time spent, monetary value is assigned based on their average hourly wage. Costs incurred by patients include costs or time spent for received care or home visits. For visits in health facilities, we collect self-reported cost data from patients.

Biospecimens: In a subset of consenting women, additional biospecimens will be collected for future analysis. These specimens are shown in Web Table 5.

Statistical Analysis

The Statistical Analysis Plan (SAP) of the ENAT study is published separately at: https://addiscontinental.edu.et/.

Sample Size

We have estimated the effect size detectable with 80% power under a cluster-randomised design, with 6 health centers per study arm. Fixing recruitment of pregnant women to 18 months, we estimated that within this time period the average health center in the proposed study site would enroll around 200 women into ANC at ≤24 weeks gestation, and would yield 112 live born infants weighed within 72 hours of life (assuming ~70% of enrolled pregnancies result in a live birth, and ~80% are followed up and weighed within 72 hours). Beyond the above determination of average cluster size, we have additionally made the following

Confidential: For Review Only

assumptions in order to estimate effect sizes detectable with 80% power: 1) mean birthweight and standard deviation as per prior studies in Gondor (mean birth weight of 2900 gm, SD 450gm) and 2) variation in distribution of weight between clusters as reflected through an coefficient of variation (k=0.01) [47]. In total this includes 2,400 pregnant mothers enrolled in 12 health centers, resulting in 1,440 live births with a birthweight within 72 hours. This sample size provides 80% power to detect a 66 gram difference in birthweight between the ENAT EIMP or routine care group in a marginal analysis (i.e. irrespective of whether mothers did or did not receive the ENP), and a 90 gm difference between ENP vs routine care (marginal analysis).

Length. With the assumptions of clusters and enrollment above, we assumed mean infant length of 49.5 cm (SD 2.4) (based on the Malawi LAIS study [48,49] and coefficient of variation k=0.008 (sector level variation in JiVitA study) [47]. For the EIMP vs. routine care comparison, we would have 80% power to detect a 3.0 mm difference in mean infant length. For the ENP vs. non-ENP comparison (marginal analysis) we would have 80% power to detect a 7.8 mm difference in infant length between the women receiving the package of enhanced nutrition-infection compared to standard nutrition care.

Study Monitoring

An external Study Monitoring Committee (SMC) is established to monitor the progress of the study, including enrollment, progress indicators and adverse events. The SMC includes an independent Ethiopian obstetrician (Dr. Delayehu Bekele) and an epidemiologist (Professor Simon Cousens). The committee met before study initiation, at mid-enrollment, and every 6 months to review study progress. Interim analysis will not be performed.

ETHICS AND DISSEMINATION

Research Ethics approval: The ENAT protocol was approved at Addis Continental Institute of Public Health (001-A1-2019) and Mass General Brigham Institutional Review Board (2018P002479). The APHI granted local permission to conduct the study.
The study is registered at ISRCTN (ISRCTN15116516). Protocol modifications will be communicated to respective IRBs and ISRCTN.

Confidentiality

All the data collected for this study is kept strictly confidential at Addis Continental Institute of Public Health on a local encrypted server. Personal identifiers are not used in study-specific

Confidential: For Review Only

forms, aside from the identifier module. Paper copies of data forms for data entry and analysis are stored in a locked file when not in use. Access to data files containing personal identifying information is limited to the principal investigators and key staff.

Ancillary care

ENAT project helps cover ancillary care related to study participation that is not covered by the health system.

Dissemination plan

ENAT study findings will be disseminated to FMoH, APHI, ARHB, Zonal health departments, woreda health offices, community representatives, and to other relevant stakeholders. Involving relevant stakeholders in the dissemination process will help to enhance ownership of the research output and the ultimate integration of findings into programs. The dissemination will be in the form of presentations in workshops, conferences, and symposiums at local, regional, national, and international levels as appropriate. In addition, reports and peer-review journal publications will be produced.

DISCUSSION

Despite the high global burden of low birthweight, preterm birth and fetal growth restriction, few interventions have demonstrated efficacy or effectiveness in the prevention of these adverse birth outcomes. Novel, integrated approaches are needed to make more substantial, scalable and sustainable impacts in order to meet the WHO's Nutrition targets. A critical feature of the ENAT study is its health systems approach and involvement of key local stakeholders in the design of intervention packages and implementation. The packaging/bundling of interventions aims to maximize their potential impact, cost-effectiveness, learning, and future scalability. This study will provide important evidence on the role of strengthening ANC programs to improve maternal health and birth outcomes, and will help inform future ANC policies in Ethiopia. ENAT will contribute to the growing body of knowledge regarding the effectiveness of BEP supplementation during pregnancy. Secondly, despite the high burden of antenatal infections, there is limited high-quality evidence that interventions to treat prenatal infections improves birth outcomes. To our knowledge, there is even more limited data on the interactions of nutrition and infections in pregnancy and this study will provide novel new insight on the potential for synergistic benefits of an integrated approach. Finally, the study will generate implementation learning on how to optimize delivery of WHO guidelines in resource limited health systems.

Confidential: For Review Only

Access to data

The final dataset will be available to the ENAT investigative team and collaborators. Anonymized study data may be made available upon request to the study PIs in accordance with Ethiopian and US regulatory guidelines.

Declaration of interests

Financial and competing interests of the PIs

ACL reports funding from the Bill & Melinda Gates Foundation, Eunice Kennedy Shriver National Institute of Child Health and Human Development and the World Health Organization. YB reports funding from FORMAS, USAID, National Institute of Environmental Health Sciences and from the Bill & Melinda Gates Foundation. BJW reports funding from National Institute of Environmental Health Sciences and from the Bill & Melinda Gates Foundation.

Funding Statement: This work was supported by the Bill & Melinda Gates Foundation grant number OPP1184363. The funder provided input on the study design, but played no role in the data collection, data analysis, interpretation of data, writing of the report or decision to submit the report for publication.

Acknowledgements

We would like to thank the ENAT partners for their support and contributions to the study, including the Amhara Regional Health Bureau, Amhara Public Health Institute, Ethiopian Federal Ministry of Health, Ethiopian Society of Obstetrics and Gynecology, General Electric, Ethiopian Society of Radiology, and Jhpiego. We acknowledge the Child Investment Fund who supported Jhpiego health systems strengthening efforts in study areas. We would also like to thank our Study Monitoring Committee, Dr. Delayehu Bekele and Professor Simon Cousens.

Author Contributions

Wrote manuscript: ACL, LCM, FW, ME, YB
Designed study/protocol: ACL, FW, LCM, EB, ME, GC, PC, SI, CL, RM, AWT, BJW, AQ, YB
Designed study materials/study tools: ACL, FW, EB, YB, MD, ME, NF, IO, WK, TS, FS, FT, ST, KY, SI, YK, CL, RM, MS, FV, AWT, YB
Trained field data collectors: FW, EB, YB, MD, ME, WK, TS, FS, FT, ST, KY, AWT, ACL, YB
Designed Data management system: NF, FS, FT, FV, AW
Trained ultrasonography: MS, BW, KY, ST
Critically reviewed/revised manuscript: all authors
All authors have met ICJME criteria.

Confidential: For Review Only

FIGURE CAPTIONS

Figure 1. Conceptual diagram showing the pathways that link maternal undernutrition, maternal infection, and infant outcomes (A, B, and C represent mechanisms, which are described in detail in the associated sections above)

Figure 2. ENAT Study Consort Diagram

Figure 3. ENAT Study Site Map, Amhara Region, Ethiopia

Confidential: For Review Only

REFERENCES

1 World Health Organization. Global nutrition targets 2025: low birth weight policy brief. Geneva, Switzerland: 2014.

2 Blencowe H, Krasevec J, de Onis M, *et al.* National, regional, and worldwide estimates of low birthweight in 2015, with trends from 2000: a systematic analysis. *Lancet Glob Health* 2019;**7**:e849–60. doi:10.1016/S2214-109X(18)30565-5

3 Katz J, Lee AC, Kozuki N, *et al.* Mortality risk in preterm and small-for-gestational-age infants in low-income and middle-income countries: a pooled country analysis. *Lancet* 2013;**382**:417–25. doi:10.1016/S0140-6736(13)60993-9

4 Christian P, Lee SE, Donahue Angel M, *et al.* Risk of childhood undernutrition related to small-for-gestational age and preterm birth in low- and middle-income countries. *Int J Epidemiol* 2013;**42**:1340–55. doi:10.1093/ije/dyt109

5 Blencowe H, Lee ACC, Cousens S, *et al.* Preterm birth-associated neurodevelopmental impairment estimates at regional and global levels for 2010. *Pediatr Res* 2013;**74 Suppl 1**:17–34. doi:10.1038/pr.2013.204

6 Murray E, Fernandes M, Fazel M, *et al.* Differential effect of intrauterine growth restriction on childhood neurodevelopment: a systematic review. *BJOG* 2015;**122**:1062–72. doi:10.1111/1471-0528.13435

7 Danaei G, Andrews KG, Sudfeld CR, *et al.* Risk factors for childhood stunting in 137 developing countries: A comparative risk assessment analysis at global, regional, and country levels. *PLoS Med* 2016;**13**:e1002164. doi:10.1371/journal.pmed.1002164

8 Han Z, Mulla S, Beyene J, *et al.* Maternal underweight and the risk of preterm birth and low birth weight: a systematic review and meta-analyses. *Int J Epidemiol* 2011;**40**:65–101. doi:10.1093/ije/dyq195

9 Kozuki N, Katz J, Lee ACC, *et al.* Short Maternal Stature Increases Risk of Small-for-Gestational-Age and Preterm Births in Low- and Middle-Income Countries: Individual Participant Data Meta-Analysis and Population Attributable Fraction. *J Nutr* 2015;**145**:2542–50. doi:10.3945/jn.115.216374

10 Peña-Rosas JP, De-Regil LM, Garcia-Casal MN, *et al.* Daily oral iron supplementation during pregnancy. *Cochrane Database Syst Rev* 2015;:CD004736. doi:10.1002/14651858.CD004736.pub5

Confidential: For Review Only

11 Keats EC, Neufeld LM, Garrett GS, *et al.* Improved micronutrient status and health outcomes in low- and middle-income countries following large-scale fortification: evidence from a systematic review and meta-analysis. *Am J Clin Nutr* 2019;**109**:1696–708. doi:10.1093/ajcn/nqz023

12 Imdad A, Bhutta ZA. Maternal nutrition and birth outcomes: effect of balanced protein-energy supplementation. *Paediatr Perinat Epidemiol* 2012;**26 Suppl 1**:178–90. doi:10.1111/j.1365-3016.2012.01308.x

13 Ota E, Hori H, Mori R, *et al.* Antenatal dietary education and supplementation to increase energy and protein intake. *Cochrane Database Syst Rev* 2015;:CD000032. doi:10.1002/14651858.CD000032.pub3

14 Buppasiri P, Lumbiganon P, Thinkhamrop J, *et al.* Calcium supplementation (other than for preventing or treating hypertension) for improving pregnancy and infant outcomes. *Cochrane Database Syst Rev* 2011;:CD007079. doi:10.1002/14651858.CD007079.pub2

15 Gilbert NM, O'Brien VP, Hultgren S, *et al.* Urinary tract infection as a preventable cause of pregnancy complications: opportunities, challenges, and a global call to action. *Glob Adv Health Med* 2013;**2**:59–69. doi:10.7453/gahmj.2013.061

16 Romero R, Oyarzun E, Mazor M, *et al.* Meta-analysis of the relationship between asymptomatic bacteriuria and preterm delivery/low birth weight. *Obstet Gynecol* 1989;**73**:576–82.

17 Honkpéhèdji YJ, Adegbite BR, Zinsou JF, *et al.* Association of low birth weight and polyparasitic infection during pregnancy in Lambaréné, Gabon. *Trop Med Int Health* 2021;**26**:973–81. doi:10.1111/tmi.13591

18 Aderoba AK, Iribhogbe OI, Olagbuji BN, *et al.* Prevalence of helminth infestation during pregnancy and its association with maternal anemia and low birth weight. *Int J Gynaecol Obstet* 2015;**129**:199–202. doi:10.1016/j.ijgo.2014.12.002

19 Abioye AI, McDonald EA, Park S, *et al.* Maternal, placental and cord blood cytokines and the risk of adverse birth outcomes among pregnant women infected with Schistosoma japonicum in the Philippines. *PLoS Negl Trop Dis* 2019;**13**:e0007371. doi:10.1371/journal.pntd.0007371

20 Goldenberg RL, Hauth JC, Andrews WW. Intrauterine infection and preterm delivery. *N Engl J Med* 2000;**342**:1500–7. doi:10.1056/NEJM200005183422007

21 Christensen DL, Kapur A, Bygbjerg IC. Physiological adaption to maternal malaria and other adverse exposure: low birth weight, functional capacity, and possible metabolic disease in adult life. *Int J Gynaecol Obstet* 2011;**115 Suppl 1**:S16-9. doi:10.1016/S0020-7292(11)60006-4

Confidential: For Review Only

22 Krawinkel MB. Interaction of nutrition and infections globally: an overview. *Ann Nutr Metab* 2012;**61 Suppl 1**:39–45. doi:10.1159/000345162

23 Vohr BR, Poggi Davis E, Wanke CA, *et al.* Neurodevelopment: The Impact of Nutrition and Inflammation During Preconception and Pregnancy in Low-Resource Settings. *Pediatrics* 2017;**139**:S38–49. doi:10.1542/peds.2016-2828F

24 Kim H, Hwang JY, Ha EH, *et al.* Association of maternal folate nutrition and serum C-reactive protein concentrations with gestational age at delivery. *Eur J Clin Nutr* 2011;**65**:350–6. doi:10.1038/ejcn.2010.267

25 Kutlesic V, Brewinski Isaacs M, Freund LS, *et al.* Executive Summary: Research Gaps at the Intersection of Pediatric Neurodevelopment, Nutrition, and Inflammation in Low-Resource Settings. *Pediatrics* 2017;**139**:S1–11. doi:10.1542/peds.2016-2828C

26 Collin SM, Baggaley RF, Pittrof R, *et al.* Could a simple antenatal package combining micronutritional supplementation with presumptive treatment of infection prevent maternal deaths in sub-Saharan Africa? *BMC Pregnancy Childbirth* 2007;**7**:6. doi:10.1186/1471-2393-7-6

27 World Health Organization (WHO). WHO recommendations on antental care for a positive pregnancy experience. 2016.

28 Central Statistical Agency CSAE, Icf. Ethiopia Demographic and Health Survey 2016. Addis Ababa, Ethiopia: : CSA and ICF 2017.

29 Ethiopia Mini Demographic and Health Survey 2019: Key indicators. Rockville, Maryland, USA: EPHI and ICF: : Ethiopian Public Health Institute (EPHI) [Ethiopia] and ICF 2019.

30 Shiferaw MB, Zegeye AM, Mengistu AD. Helminth infections and practice of prevention and control measures among pregnant women attending antenatal care at Anbesame health center, Northwest Ethiopia. *BMC Res Notes* 2017;**10**:274. doi:10.1186/s13104-017-2609-6

31 Belyhun Y, Medhin G, Amberbir A, *et al.* Prevalence and risk factors for soil-transmitted helminth infection in mothers and their infants in Butajira, Ethiopia: a population based study. *BMC Public Health* 2010;**10**:21. doi:10.1186/1471-2458-10-21

32 Workneh F, Eglovitch M, Shiferaw T, *et al.* Dietary practices among pregnant women in rural amhara, ethiopia. *Curr Dev Nutr* 2021;**5**:698–698. doi:10.1093/cdn/nzab045_080

Confidential: For Review Only

33 Baye E, Abate FW, Eglovitch M, *et al.* Effect of birthweight measurement quality improvement on low birthweight prevalence in rural Ethiopia. *Popul Health Metr* 2021;**19**:35. doi:10.1186/s12963-021-00265-0

34 Members Of An Expert Consultation On Nutritious Food Supplements For Pregnant And Lactating Women. Framework and specifications for the nutritional composition of a food supplement for pregnant and lactating women (PLW) in undernourished and low income settings. *Gates Open Res* Published Online First: 2019. doi:10.21955/gatesopenres.1116379.1

35 Institute of Medicine. Dietary Reference Intakes (DRIs): Recommended Dietary Allowances and Adequate Intakes. Washington, DC: The National Academies Press

36 Federal Democratic Republic of Ethiopia Ministry of Health. National Guidelines for the Management of Sexually Transmitted Infections Using Syndromic. Addis Ababa, Ethiopia: 2015.

37 World Health Organization. Soil-transmitted helminth infections. 2020.https://www.who.int/news-room/fact-sheets/detail/soil-transmitted-helminth-infections (accessed 8 Sep2021).

38 R Core Team. *R: A language and environment for statistical computing*. Vienna, Austria: R Foundation for Statistical Computing 2021.

39 Workicho A, Belachew T, Feyissa GT, *et al.* Household dietary diversity and Animal Source Food consumption in Ethiopia: evidence from the 2011 Welfare Monitoring Survey. *BMC Public Health* 2016;**16**:1192. doi:10.1186/s12889-016-3861-8

40 International Fetal and Newborn Growth Consortium. The International Fetal and Newborn Growth Standards for the 21st Century(INTERGROWTH-21st) Study Protocol. 2009.

41 de Onis M, Onyango AW, Borghi E, *et al.* Comparison of the World Health Organization (WHO) Child Growth Standards and the National Center for Health Statistics/WHO international growth reference: implications for child health programmes. *Public Health Nutr* 2006;**9**:942–7. doi:10.1017/phn20062005

42 Bloem M. The 2006 WHO child growth standards. *BMJ* 2007;**334**:705–6. doi:10.1136/bmj.39155.658843.BE

43 The world health report 2000 - Health systems: improving performance. Geneva, Switzerland: : World Health Organization 2000.

Confidential: For Review Only

44 Lu C, Tsai S, Ruhumuriza J, *et al.* Tracking rural health facility financial data in resource-limited settings: a case study from Rwanda. *PLoS Med* 2014;**11**:e1001763. doi:10.1371/journal.pmed.1001763

45 Tsai S, Umugiraneza G, Ruhumuriza J, *et al.* Training manual on tracking financial data in health facilities in Kayonza and Kirehe. Boston, MA: : Program in Global Health Economics and Social Change, Harvard Medical School 2013.

46 Lu C, Tsai S, Ruhumuriza J, *et al.* Obtaining financing data for local health facilities in two districts of Rwanda: General guidelines. Boston, MA: : Program in Global Helath Economics and Social Change, Harvard Medical School 2013.

47 West KP, Shamim AA, Mehra S, *et al.* Effect of maternal multiple micronutrient vs iron-folic acid supplementation on infant mortality and adverse birth outcomes in rural Bangladesh: the JiVitA-3 randomized trial. *JAMA* 2014;**312**:2649–58. doi:10.1001/jama.2014.16819

48 Luntamo M, Kulmala T, Mbewe B, *et al.* Effect of repeated treatment of pregnant women with sulfadoxine-pyrimethamine and azithromycin on preterm delivery in Malawi: a randomized controlled trial. *Am J Trop Med Hyg* 2010;**83**:1212–20. doi:10.4269/ajtmh.2010.10-0264

49 Luntamo M, Kulmala T, Cheung YB, *et al.* The effect of antenatal monthly sulphadoxine-pyrimethamine, alone or with azithromycin, on foetal and neonatal growth faltering in Malawi: a randomised controlled trial. *Trop Med Int Health* 2013;**18**:386–97. doi:10.1111/tmi.12074

Confidential: For Review Only

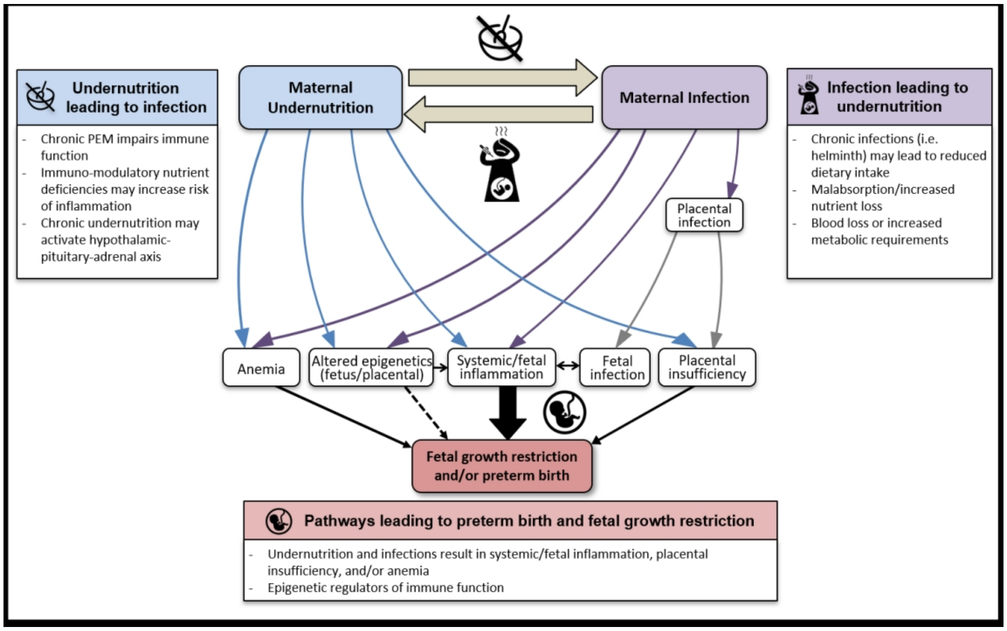

319x199mm (118 x 118 DPI)

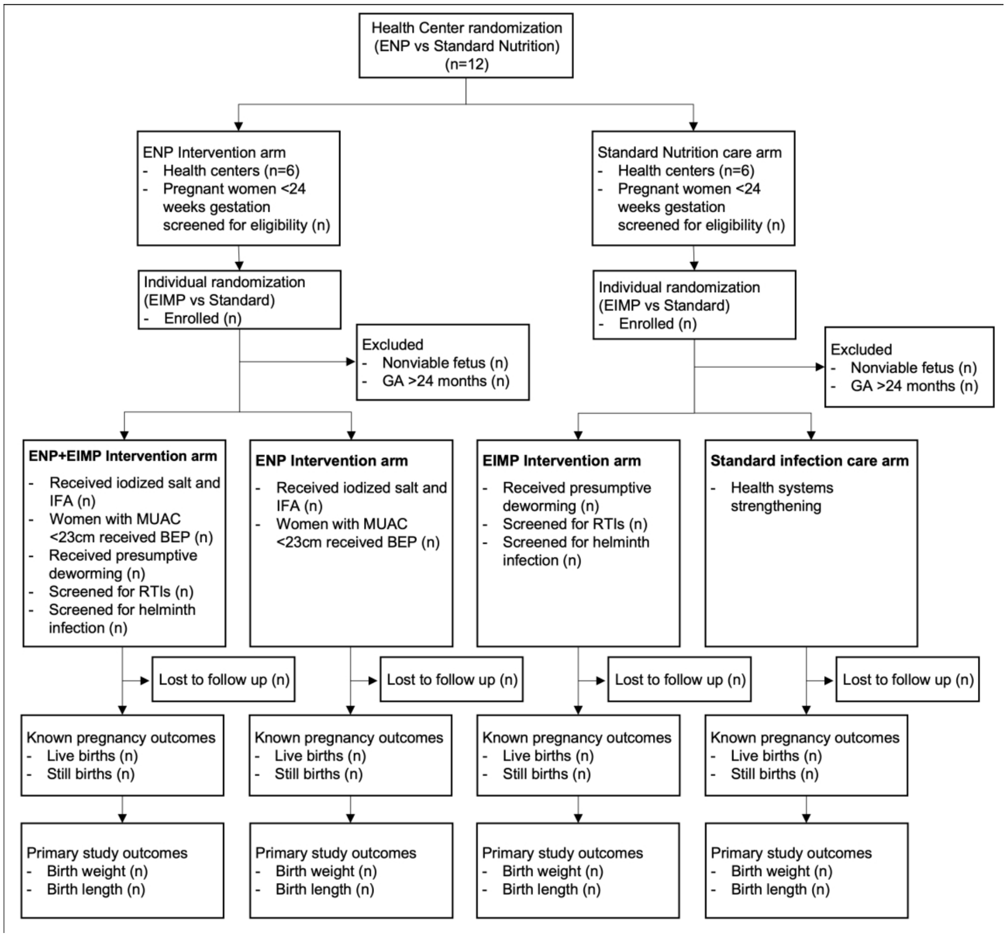

653x606mm (118 x 118 DPI)

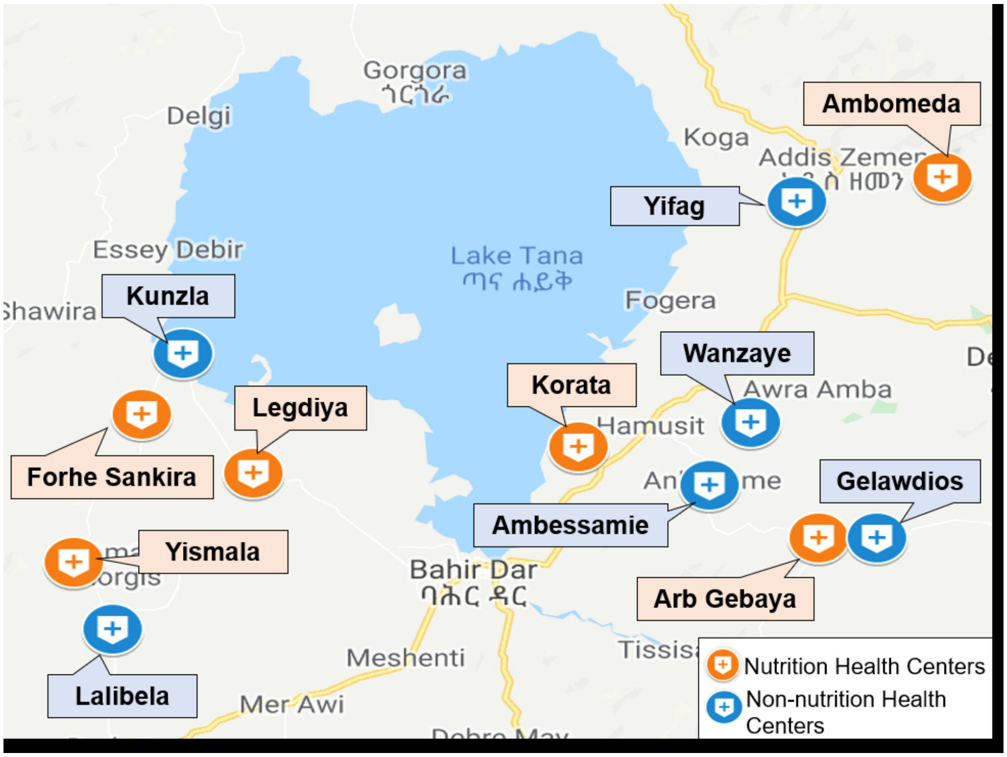

210x157mm (118 x 118 DPI)

WEBAPPENDIX

Web Table 1. WHO Recommendations for Antenatal Care

**Nutritional Interventions**	
A.1.1: Counselling about healthy eating and keeping physically active during pregnancy is recommended for pregnant women to stay healthy and to prevent excessive weight gain during pregnancy.	Recommended
A.1.2: In undernourished populations, nutrition education on increasing daily energy and protein intake is recommended for pregnant women to reduce the risk of low-birth-weight neonates.	Context-specific recommendation
A.1.3: In undernourished populations, balanced energy and protein dietary supplementation is recommended for pregnant women to reduce the risk of stillbirths and small-for-gestational-age neonates.	Context-specific recommendation
A.2.1: Daily oral iron and folic acid supplementation with 30 mg to 60 mg of elemental iron and 400 µg (0.4 mg) of folic acid is recommended for pregnant women to prevent maternal anaemia, puerperal sepsis, low birth weight, and preterm birth.	Recommended
A.2.2: Intermittent oral iron and folic acid supplementation with 120 mg of elemental iron and 2800 µg (2.8 mg) of folic acid once weekly is recommended for pregnant women to improve maternal and neonatal outcomes if daily iron is not acceptable due to side-effects, and in populations with an anaemia prevalence among pregnant women of less than 20%.	Context-specific recommendation
A.3: In populations with low dietary calcium intake, daily calcium supplementation (1.5–2.0 g oral elemental calcium) is recommended for pregnant women to reduce the risk of pre-eclampsia.	Context-specific recommendation
A.4: Vitamin A supplementation is only recommended for pregnant women in areas where vitamin A deficiency is a severe public health problem, to prevent night blindness.	Context-specific recommendation
A.5: Zinc supplementation for pregnant women is only recommended in the context of rigorous research.	Context-specific recommendation (research)
A.10: For pregnant women with high daily caffeine intake (more than 300 mg per day), lowering daily caffeine intake during pregnancy is recommended to reduce the risk of pregnancy loss and low-birth-weight neonates.	Context-specific recommendation
**Maternal Assessment**	
B.1.1: Full blood count testing is the recommended method for diagnosing anaemia in pregnancy. In settings where full blood count testing is not available, on-site haemoglobin testing with a haemoglobinometer is recommended over the use of the haemoglobin colour scale as the method for diagnosing anaemia in pregnancy.	Context-specific recommendation
B.1.2: Midstream urine culture is the recommended method for diagnosing asymptomatic bacteriuria (ASB) in pregnancy. In settings where urine culture is not available, on-site midstream urine Gram-staining is recommended over the use of dipstick tests as the method for diagnosing ASB in pregnancy.	Context-specific recommendation
B.2.4: One ultrasound scan before 24 weeks of gestation (early ultrasound) is recommended for pregnant women to estimate gestational age, improve detection of fetal anomalies and multiple pregnancies, reduce induction of labour for post-term pregnancy, and improve a woman's pregnancy experience.	Recommended
**Preventive Measures**	
C.1: A seven-day antibiotic regimen is recommended for all pregnant women with asymptomatic bacteriuria (ASB) to prevent persistent bacteriuria, preterm birth and low birth weight.	Recommended
C.2: Antibiotic prophylaxis is only recommended to prevent recurrent urinary tract infections in pregnant women in the context of rigorous research.	Context-specific recommendation (research)
C.4: In endemic areas, preventive anthelminthic treatment is recommended for pregnant women after the first trimester as part of worm infection reduction programmes.	Context-specific recommendation

Confidential: For Review Only

Web Table 2. Super Cereal Corn Soya Blend Composition (per daily 200 gm serving)

NUTRIENT	Units	ENAT CSB CONTENT (200 gm)	Recommended Target*
Energy	Kcal	760	250-500kcal per daily serving**
Protein	gm	28	14-18 g**
Fat	%	12%	10-60% of energy
Vitamin A	mcg RE	2076	550-770***
Vitamin D	mcg	22.1	10-15
Vitamin E	mg	16.6	16-19
Vitamin K	mcg	60	72-90
Vitamin B1	mg	0.4	1.2-1.4
Vitamin B2	mg	2.8	1.3-1.6
Vitamin B3	mg	16	14-18
Folic Acid	mcg	220	400-600
Vitamin B6	mg	2.0	1.7-2.0
Vitamin C	mg	180	100-120
Calcium	mg	724	500-1500
Iron	mg	8	22-27
Iodine	mcg	80	209-290
Phosphorous	mg	560	300-700
Zinc	mg	10	15-20

* Targets are results of an Expert Consultation held at Bill and Melinda Gates Foundation.[50]
** Energy balance from macronutrient: portion size can be doubled in settings of high energy gaps, such as maternal malnutrition and where prevalence of low birthweight is high.
*** Micronutrient recommendations were primarily based upon Institute of Medicine (IOM) estimated average requirement (EAR) and recommended dietary allowances (RDA) values.

Confidential: For Review Only

Web Table 3. Classification of UTI in ENAT study

UTI Terminology	Definition
High-burden growth	bacteriuria of >10^5 colony forming units (CFU) per 1mL of urine of a single uropathogen [49],
Intermediate growth	bacteriuria with >10^3 -10^5 CFU/mL of a single uropathogen,
Contamination	bacterial growth of $\geq$3 micro-organism OR growth of a non-urinary tract pathogen.
UTI symptoms	dysuria, urinary frequency, urinary urgency, hematuria, abdominal pain, fever, OR flank pain
Symptomatic intermediate growth	women with intermediate burden growth and UTI symptoms (as above)
Asymptomatic bacteriuria	women with high burden bacterial growth without UTI symptoms
Cystitis	women with positive urine culture (high burden or intermediate growth) and symptoms of dysuria, urinary frequency, hematuria, urinary urgency or suprapubic tenderness, without upper urinary tract symptoms (fever, chills, flank or back pain) [49]
Pyelonephritis	women with positive urine culture and systemic symptoms (fever, chills, flank pain or back pain) [49].

Confidential: For Review Only

Web Table 4. Recommended Treatments for Parasitic Stool Infections FMHACA Standard Treatment Guideline,3rd Edition 2014

Intestinal parasite	Recommended treatment, and alternative
Entameoba Histolytica	Metronidazole 500 mg P.O. TID x 5-7 days
Giardia lamblia	Tinidazole 2 gm po single dose<br>Or Alternative:<br>Metronidazole 500 mg po TID x 5 days
**Ascariasis**<br><br>***Ascaris lumbricoids***	Mebendazole, 500mg P.O. once or<br>Albendazole, 400mg P.O. as a single dose<br>Or Alternative:<br>Pyrantel pamoate, 700mg P.O. as a single dose
**Hookworm infestation**<br>***Necator americanus or Ancylostoma duodenale***	Mebendazole, 500mg stat or 100mg P.O. BID for 3 days<br>Albendazole, 400mg P.O. as a single dose<br>Or Alternative:<br>Pyrantel pamoate, 700mg P.O. as a single dose
**Enterobiasis**<br><br>***Enterobius Vermicularis***	Mebendazole, 100mg P.O. BID for 3 days or<br>Or Alternative:<br>Albendazole, 400mg P.O. as a single dose
**Trichuriasis**<br>***T.tricura [Whipworm]***	Mebendazole, 500mg P.O. single dose or,<br>Or Alternative:<br>Albendazole, 400mg, P.O. for three days
**Taeniasis**<br><br>***T.saginata or T.solium***	Praziquantel P.O. 600mg or 10mg/Kg, single dose<br>Or Alternative:<br>Niclosamide, 2g in a single dose P.O.
Hymenolepis nana	Praziquantel, 25mg/kg or 1800mg P.O. single dose<br>Alternative:<br>Niclosamide, 2g P.O. on the first day followed by 1g QD for 6 days
Schistosoma Mansoni	Praziquantel P.O. 1200 mg single dose (or 600 mg po in 2 doses)
**Strongyloidiasis**<br><br>***Strongloidexs stercolaries***	Albendazole 400mg P.O. BID for three consecutive days.

Confidential: For Review Only

Web Table 5. Biological Specimens Collected in ENAT Study

A table of biospecimens and planned lab testing is presented below. Biospecimens collected for immediate analyses at the APHI laboratory are stored at room temperature using special collection containers and swabs designed to meet this storage requirement. Samples collected for future analyses are aliquoted to appropriate containers and stored at APHI for long term storage in a specially procured freezer for this purpose. All samples are stored for up to 5 years.

ENAT Bio-specimens

			Antenatal		Post-natal	
			≤24 wks	~30-34 wks	0 -<72 hrs	
Biospecimen	N (Total)	Planned lab testing	Enroll	ANCFU2	Birth	1-2 mo.
Maternal urine	800	Culture, subset of 600 for iodine concentration, selenium, environmental toxins	■	■		
Maternal vaginal swab	150	STI/RTI	■			
Maternal blood serum	700	Nutrient levels, TORCH infections	■	■		
blood spot	1000	Inflammation, thyroid, nutrients, miRNAs	■	■		
VAMS + DBS	120	metabolomics, proteomics	■	■	■	■
Maternal vaginal swab	150	Microbiome, metabolome	■	■		■
Maternal stool	120	Microbiome	■	■		■
Maternal oral sample	120	Microbiome	■	■		■
Umbilical cord blood/spot	650	Inflammation, thyroid, nutrients, miRNAs			■	
Infant blood (VAMS + DBS)	120	Metabolomics, Proteomics			■	■
Infant stool	160	Microbiome			■	■
Infant oral sample	100	Microbiome, CMV			■	■
Breast milk	100	Nutrient composition, microbiome				■

For peer review only

Web Form 1. Model Informed Consent Form

Information sheet and Consent: ENP Arm

Name of the organization: Addis Continental Institute of Public Health
Name of the Sponsor: Bill and Melinda Gates Foundation and Brigham and Women's Hospital

Good morning/Good afternoon.
My name is ________________________________. I am a study nurse at Addis Continental Institute of Public Health (ACIPH). ACIPH is doing the ENAT study together with the Federal Ministry of Health, Amhara Regional Health Bureau, Amhara Public Health Institute, and other partners.

Purpose: We are asking you to become part of a research study that has a goal to improve the health of the pregnant women and their babies. The main goal of the study is to see if babies will grow better in the womb if pregnant women are provided with better nutrition and are treated for infections in pregnancy. Pregnant women will be enrolled if they are less than 6 months pregnant and will be followed up to 6 months after the baby's birth. The total number of pregnant women enrolled in this study will be 3,600.

Explanation of study procedures:
We will measure your height, weight, and middle upper arm circumference. If your middle-upper arm circumference is >23 cm, you will be given iodized salt and iron folate to be consumed daily until you deliver your baby. Whenever you come to the health center, a midwife will give nutrition counseling about nutrients in IFA and iodized salt and maintaining appropriate weight gain during pregnancy for you and your baby.

If the study nurse finds that your arm size is small (middle upper arm circumference <23 cm), you will also be provided a nutritious food supplement of a corn soy blend flour. You may use the corn soy blend to make a daily serving to take on each day. These supplements will be given to you at the health center once a month until you give birth. We believe this will give the unborn baby the nutrition s/he needs to grow better.

We also hope that treating infections in pregnancy will help you to have a healthier pregnancy, increase the chances that the baby will deliver at the expected due date, and help the baby grow better in the womb.

If you decide to participate in the study, you will be put into one of two groups. The study team will choose which group you will be put into randomly. There is no way to tell which group you will be in. Neither you, the study staff, nor the health workers can choose what group you will be in. You will have an equal chance of being placed in either of the two group.

If you are assigned to the first group, you will be tested and treated for certain infections. You will be given deworming medication during the second trimester of your pregnancy, and you will be asked to provide a stool sample to screen and treat for persistent helminthiasis (worms) in the

Confidential: For Review Only

third trimester. You also be asked to give a sample of your urine to test for infection that may affect your health and the health of the baby. The study nurse and laboratory personnel will demonstrate to you how to collect the sample. The urine sample will be analyzed at APHI and you will be asked to come back to the health center within 7 days to get results from your tests.

If you are not in the first group, you will receive the routine antenatal care that is provided at the health center. You will not receive any additional testing or treatment.

After your baby is born, we will need to very carefully measure your baby's height, weight, chest, head and arm size. It is important these measurements happen within three days of giving birth. Even if you give birth at home, a trained person will visit you to carefully take them. We may also measure your baby at a later study visits at 1, 3, and 6 months.

We ask for your permission to be contacted during the study to remind you of study visits, to notify you that your test results have arrived, visit you in case of birth at home and/or to allow us to come to your house to measure the level of iodine in the salt that you use for household consumption. We will remind you about your visit by phone call or text message. If you do not respond, we may visit your home. Please show below how you agree to be contacted:

☐ Text message (to you or a family member with a phone)

☐ Phone call (to you or a family member with a phone)

☐ Home visit (If you do not come to the facility for your visit, a study staff may visit you at your home.)

Participants for these activities are pregnant women, less than 6 months pregnant, who come for ANC at health centers at the study site during the study period. If you are willing to be a part of the study, you need to understand and sign this form. All your personal information for this study will be kept private in our office where no one will be able to see it. Your identity will not be used in any reports or publications that come from the study.

Risk or discomforts: You may feel embarrassed or nervous answering some questions about your health and your baby's health. You do not have to answer every question if you do not want to.

There also maybe risk of aside effect to a medication. If you are treated for an infection, all of the medicines that will be given by your midwife are routine and considered safe to have while pregnant and are not known to hurt the baby.

We will give you our phone number so that you can call us with any concerns about bad reactions to any of the medications. We will help you to be seen and treated at the health center or nearby hospital and help pay the cost of treatment or transportation if it's absolutely necessary.

Benefits: All mothers who join in the study will get an ultrasound and at the third trimester (28 weeks of GA), that will help make sure your pregnancy is healthy. You will be screened for

Confidential: For Review Only

anemia, get iodized salt that may help you to be more nourished and help your baby grow. If you have poor nutrition, you will get a corn soy blend flour containing nutrients to eat every day. This supplement may also help improve your and your baby's nutrition during the pregnancy.

Some mothers will be tested for infections and get treatment that will help your and your baby's health. Though you may not get money or gifts, being a part of the study is very important in making programs that can help mother and child health, especially in helping babies grow and develop better in the womb.

Confidentiality and Anonymity: Information about you that we will collect by this research project will be kept. Your personal information will not be seen by anyone except the study team, and it will only be used for this research. No identities will be used in any reports or publications resulting from the study.

Right to Refuse or Withdraw: Being a part of this study and everything you are asked to do is completely voluntary. You have the full right to refuse from being a part of this study. You also have the full right to stop being a part of this study at any time you wish to. Refusing to be in the study or stopping will not affect the services that you get from the health facility.

We thank you very much for your time. If you have any questions, you can call:
Professor Yemane Berhane: Addis Continental Institute of Public Health,
Tel: +251 114 168207/ + 251 911219785 (mobile)
E-mail: yemaneberhane@addiscontinentual.edu.et OR yemaneberhane@gmail.com

Addis Continental Institute of Public Health, Tel: +251 116 390039
E-mail: aciphaddis@gmail.com

Confidential: For Review Only

Consent Form

I have read this information and decided to be a part of this study. The general purpose of study has been explained well to me and I was given the chance to ask questions. I understand that I can stop my participation and anything for the study at any time.

______________________	______________	_______________
Participants name	Participants age	ENAT Study ID
______________________	______________	
Participants signature	Date (DD/MM/YY)	
______________________	______________	_______________
Study nurse name	Study nurse signature	Date (DD/MM/YY)

If illiterate:

I have seen the careful reading of the consent form to the potential participant and the participant has been able to ask questions. I agree that the participant has given consent freely.

______________________	______________	_______________
Witness name	Witness signature	Date (DD/MM/YY)
______________________	______________	_______________
Participants name	Participants age	ENAT Study ID

Thumb print of participant Date (DD/MM/YY)

______________________	______________	_______________
Study nurse name	Study nurse signature	Date (DD/MM/YY)

Confidential: For Review Only

BMJ Paediatrics Open

The Enhancing Nutrition and Antenatal Infection Treatment (ENAT) Study: Protocol of a pragmatic clinical effectiveness study to improve birth outcomes in Ethiopia

Journal:	*BMJ Paediatrics Open*
Manuscript ID	bmjpo-2021-001327.R1
Article Type:	Protocol
Date Submitted by the Author:	02-Nov-2021

Complete List of Authors:	Lee, Anne; Brigham and Women's Hospital, Pediatric Newborn Medicine; Harvard Medical School<br>Abate, Firehiwot Workneh; Addis Continental Institute of Public Health<br>Mullany, Luke C.; Johns Hopkins Bloomberg School of Public Health, Department of International Health<br>Baye, Estifanos; Brigham and Women's Hospital, Department of Pediatric Newborn Medicine<br>Berhane, Yoseph Yemane; Addis Continental Institute of Public Health<br>Derebe, Mulatu Melese; Amhara Public Health Institute<br>Eglovitch, Michelle; Brigham and Women's Hospital, Department of Pediatric Newborn Medicine<br>Fasil, Nebiyou; Addis Continental Institute of Public Health<br>Olson, Ingrid; Brigham and Women's Hospital, Department of Pediatric Newborn Medicine<br>Kidane, Workagegnehu Tarekegn; Addis Continental Institute of Public Health<br>Shiferw, Tigest; Addis Continental Institute of Public Health<br>Shiferie, Fisseha; Addis Continental Institute of Public Health<br>Tsegaye, Fitsum; Addis Continental Institute of Public Health<br>Tsegaye, Sitota; Addis Continental Institute of Public Health<br>Yibeltal, Kalkidan; Addis Continental Institute of Public Health<br>Chan, Grace; Boston Children's Hospital, Harvard Medical School, Department of Medical Critical Care; Harvard TH Chan School of Public Health, Department of Epidemiology<br>Christian, Parul; Johns Hopkins Bloomberg School of Public Health, Department of International Health<br>Isanaka, Sheila; Harvard TH Chan School of Public Health, Departments of Nutrition and Global Health and Population<br>Kang, Yunhee; Johns Hopkins Bloomberg School of Public Health, Department of International Health<br>Lu, Chunling; Brigham and Women's Hospital, Division of Global Health Equity; Harvard Medical School, Department of Global Health and Social Medicine<br>Molina, Rose ; Beth Israel Deaconess Medical Center<br>Stojanov, Michele; Harvard Medical School, Department of Obstetrics and Gynecology<br>Van Dyk, Fred; Johns Hopkins Bloomberg School of Public Health, Department of International Health

	Tadesse, Amare; Addis Continental Institute of Public Health; London School of Hygiene and Tropical Medicine, Department of Infectious Disease Epidemiology<br>Wylie, Blair; Harvard Medical School; Beth Israel Deaconess Medical Center, Department of Obstetrics and Gynecology<br>Worku, Alemayehu; Addis Continental Institute of Public Health<br>Berhane, Yemane; Addis Continental Institute of Public Health

Keywords:	Neonatology, Growth

SCHOLARONE™ Manuscripts

I, the Submitting Author has the right to grant and does grant on behalf of all authors of the Work (as defined in the below author licence), an exclusive licence and/or a non-exclusive licence for contributions from authors who are: i) UK Crown employees; ii) where BMJ has agreed a CC-BY licence shall apply, and/or iii) in accordance with the terms applicable for US Federal Government officers or employees acting as part of their official duties; on a worldwide, perpetual, irrevocable, royalty-free basis to BMJ Publishing Group Ltd ("BMJ") its licensees and where the relevant Journal is co-owned by BMJ to the co-owners of the Journal, to publish the Work in this journal and any other BMJ products and to exploit all rights, as set out in our licence.

The Submitting Author accepts and understands that any supply made under these terms is made by BMJ to the Submitting Author unless you are acting as an employee on behalf of your employer or a postgraduate student of an affiliated institution which is paying any applicable article publishing charge ("APC") for Open Access articles. Where the Submitting Author wishes to make the Work available on an Open Access basis (and intends to pay the relevant APC), the terms of reuse of such Open Access shall be governed by a Creative Commons licence – details of these licences and which Creative Commons licence will apply to this Work are set out in our licence referred to above.

Other than as permitted in any relevant BMJ Author's Self Archiving Policies, I confirm this Work has not been accepted for publication elsewhere, is not being considered for publication elsewhere and does not duplicate material already published. I confirm all authors consent to publication of this Work and authorise the granting of this licence.

Confidential: For Review Only

The Enhancing Nutrition and Antenatal Infection Treatment (ENAT) Study: Protocol of a pragmatic clinical effectiveness study to improve birth outcomes in Ethiopia

Anne CC Lee[1,2*], Firehiwot Workneh Abate[3], Luke C. Mullany[4], Estifanos Baye[1], Yoseph Yemane Berhane[3], Mulatu Melese Derebe[5], Michelle Eglovitch[1], Nebiyou Fasil[3], Ingrid E Olson[1], Workagegnehu Tarekegn Kidane[3], Tigest Shiferw[3], Fisseha Shiferie[3], Fitsum Tsegaye[3], Sitota Tsegaye[3], Kalkidan Yibeltal[3], Grace J. Chan[2,6,7], Parul Christian[4], Sheila Isanaka[8], Yunhee Kang[4], Chunling Lu[2,9], Rose L. Molina[2,10], Michele D. Stojanov[10], Fred Van Dyk[4], Amare Worku Tadesse[3,11], Blair J. Wylie[2,9], Alemayehu Worku[3], Yemane Berhane[3]

[1]Department of Pediatric Newborn Medicine, Brigham and Women's Hospital, Boston, MA, USA
[2]Harvard Medical School, Boston, Massachusetts, USA
[3]Addis Continental Institute of Public Health, Addis Ababa, Ethiopia
[4] Department of International Health, Johns Hopkins Bloomberg School of Public Health, Baltimore, MD, USA.
[5]Amhara Public Health Institute, Bahir Dar, Ethiopia
[6]Department of Epidemiology, Harvard T.H. Chan School of Public Health, Boston, MA, USA
[7] Department of Medical Critical Care, Boston Children's Hospital, Harvard Medical School, Boston, Massachusetts, USA
[8]Departments of Nutrition and Global Health and Population, Harvard T.H. Chan School of Public Health, Boston, MA, USA
[9] Division of Global Health Equity, Brigham and Women's Hospital, Boston, Massachusetts, USA
[10]Department of Obstetrics and Gynecology, Beth Israel Deaconess Medical Center, Harvard Medical School, Boston, MA, USA
[11]Department of Infectious Disease Epidemiology, London School of Hygiene and Tropical Medicine, London, UK

Corresponding Author:
Anne CC Lee
75 Francis St, Boston MA 012115
617-732-8343
Alee6@bwh.harvard.edu

Word Count: 4400

Confidential: For Review Only

ABSTRACT

Introduction: The WHO Nutrition Target aims to reduce the global prevalence of low birth weight 30% by 2025. The Enhancing Nutrition and Antenatal Infection Treatment (ENAT) study will test the impact of packages of pregnancy interventions to enhance maternal nutrition and infection management on birth outcomes in rural Ethiopia.

Methods and analysis: ENAT is a pragmatic, open-label, 2x2 factorial, randomised clinical effectiveness study implemented in 12 rural health centers in Amhara, Ethiopia. Eligible pregnant women presenting at antenatal care (ANC) visits at <24 weeks gestation are enrolled (n=2400). ANC quality is strengthened across all centers. Health centers are randomised to receive an enhanced nutrition package (ENP) or standard nutrition care, and within each health center, individual women are randomised to receive an enhanced infection management package (EIMP) or standard infection care. At ENP centers, women receive a regular supply of adequately iodized salt and iron-folate (IFA), enhanced nutrition counseling, and those with mid-upper arm circumference <23 cm receive a micronutrient fortified balanced energy protein supplement (corn soya blend) until delivery. In standard nutrition centers, women receive routine counseling and IFA. EIMP women have additional screening/treatment for urinary and sexual/reproductive tract infections and intensive deworming. Non-EIMP women are managed syndromically per Ministry of Health Guidelines. Participants are followed until 1 month postpartum, and a subset until 6 months. The primary study outcomes are newborn weight and length measured at <72 hours of age. Secondary outcomes include preterm birth, low birthweight, and stillbirth rates; newborn head circumference; infant weight and length for age z-scores at birth; maternal anemia; and weight gain during pregnancy.

Ethics and dissemination: ENAT is approved by the Institutional Review Boards of Addis Continental Institute of Public Health (001-A1-2019) and Mass General Brigham (2018P002479). Results will be disseminated to local and international stakeholders.

ISRCTN Registration: 15116516

Funding: Bill & Melinda Gates Foundation

Protocol: Version 4.0 (August 2021)

Confidential: For Review Only

Article Summary

What is known about the subject:

- In low- and middle-income countries, maternal undernutrition is prevalent and a major risk factor for adverse birth outcomes, including spontaneous preterm birth, low birth weight and small for gestational age infants.
- Maternal infections in pregnancy are also common, yet under-recognized risk factors for preterm birth and poor fetal growth in low- and middle-income countries.
- Beyond their independent effects, maternal infections and nutritional status may have synergistic effects on fetal growth and gestational length.

What this study hopes to add:

- Increase the evidence-base on the role of antenatal infection management on maternal and birth outcomes in a low resource rural setting in Sub-Saharan Africa.
- Increase the evidence-base on the role of targeted, fortified balanced energy protein supplementation on maternal and birth outcomes in a low resource rural setting Sub-Saharan Africa.
- Evaluate the benefit of developing intervention packages with local stakeholders that are implemented within existing health systems

Confidential: For Review Only

INTRODUCTION

The World Health Organization (WHO) Third Global Nutrition Target aims to reduce the proportion of babies born low birthweight (LBW, <2500 grams) by 30% by the year 2025[1]. Approximately 20.5 million infants were born LBW in 2015, with 91% from low-middle income countries (LMIC) and 24% in Sub-Saharan Africa [2]. The main etiologies of low birthweight are preterm birth (<37 gestational weeks) and fetal growth restriction, commonly classified as small-for-gestational-age (SGA) at birth. Preterm and SGA infants carry increased risk of mortality, morbidity, childhood stunting, neurodevelopmental impairment, and adult chronic disease [3–7]. Prevention of preterm birth and SGA is a key public health strategy to improve child survival and health in LMICs.

In LMICs, maternal undernutrition is prevalent and a major risk factor for adverse birth outcomes, including spontaneous preterm birth, LBW and SGA [8,9]. Interventions to improve maternal nutritional status in pregnancy, including iron-folate [10], multiple-micronutrients [11], and balanced protein-energy (BEP) supplementation [12,13], have been individually tested and found to be effective in increasing mean birthweight. However, lower than expected benefit has been observed with the individual nutritional interventions [10,12–14]. Greater effect sizes are noted in undernourished women, and there is a need for additional studies with standardized supplementation criteria as well as the combination of BEP and micronutrient supplementation [12,13].

Maternal infections in pregnancy are also common, yet under-recognized risk factors for preterm birth and poor fetal growth in LMICs. Urinary tract infection or asymptomatic bacteriuria may affect 9-80% of pregnancies in sub-Saharan Africa [15] and are associated with a 2-fold elevated risk of preterm delivery [16]. Helminthic infections are prevalent and associated with systemic inflammation, LBW and preterm birth [17–19]. Genital tract infections may ascend the reproductive tract and lead to infection and inflammation in the amniotic fluid, predisposing to preterm birth [20]. In LMICs, screening and treatment of genitourinary tract infections during routine antenatal care (ANC) is infrequent due to lack of resources and capacity for laboratory testing. While epidemiologic data has consistently established associations between prenatal infections and adverse pregnancy outcomes, there is limited evidence on the effectiveness of prenatal interventions to screen and treat infections to prevent LBW in LMICs.

Confidential: For Review Only

Beyond their independent effects, maternal infections and nutritional status may have synergistic effects on fetal growth and gestational length [21–25]. Figure 1 depicts several pathways linking maternal nutrition and infections in pregnancy. This framework provides the basis for the hypothesis that targeting both risk factors in pregnancy may lead to more substantial, and potentially synergistic, improvements in fetal growth and pregnancy length [26].

This manuscript details the study protocol for the Enhancing Nutrition and Antenatal Infection Treatment (ENAT) study in Amhara region, Ethiopia. In 2016, WHO released recommendations on an evidence-based, core package of ANC to optimize the pregnancy experience and outcomes [27], include guidelines on nutrition and infection management in pregnancy (Web Table 1). In Ethiopia, not all recommendations have been adopted or achieved high level of coverage or quality in ANC. The primary aim of the ENAT study is to determine the impact of WHO-recommended ANC interventions to optimize maternal nutrition and manage maternal pregnancy infections on infant birth size in Amhara, Ethiopia. We hypothesize that the nutrition and infection management packages will independently increase newborn birth weight and length, and that the combined effect of both packages will be greater than either alone.

METHODS AND ANALYSIS

Study Design

The ENAT study is a 2x2 factorial pragmatic, open-label, randomised clinical effectiveness study with cluster randomisation of the enhanced nutrition package (ENP) versus standard care (non-ENP), and individual level randomisation of an enhanced infection management package (EIMP) versus standard care (non-EIMP) (Figure 2).

Study Setting

The ENAT study site was established in 2018 as a partnership with the Addis Continental Institute of Public Health (ACIPH), Amhara Regional Health Bureau, Amhara Public Health Institute and Brigham and Women's Hospital. The Amhara region has low-resourced health systems and poor health indicators. As per the 2016 Ethiopian Demographic Health Services (EDHS) data, Amhara had the country's highest rates of neonatal mortality (47 per 1000 live births) and LBW (22.2%), and high maternal mortality rate (412 per 100,000 live births) [28].

Confidential: For Review Only

Rates of any prenatal care and institutional delivery and were 82.6% and 54.2%, respectively [29]. One in four women of reproductive age are underweight (body mass index<18.5 kg/m^2) [28] and geohelminth infections are prevalent, ranging from 21.1-43.5% [30,31].

The ENAT study is conducted in 12 rural health centers (each serving ~25,000 population) in West Gojjam (South and North Achefer districts [woredas]) and South Gondar (Dera and Libokemem districts) zones, Amhara (Figure 3, Site Map). The districts were chosen in collaboration with the Amhara Regional Health Bureau based on the high rates of undernutrition, risk of low birth weight, need for nutritional programs, and proximity to the regional laboratory. The study health centers were chosen based on accessibility, total ANC volume (minimum 250 women presenting to ANC/year), and infrastructure (functional laboratory).

Patient and public involvement

Prior to the study, formative work was conducted with a range of community members (mothers, families, community and religious leaders, health providers) [32]. This feedback directly informed the design of the study interventions, packages, and their implementation. Community sensitization was performed prior to initiating the study.

Study participants and recruitment

Pregnant women are recruited from ANC visits in designated ENAT study health centers. A study nurse explains study procedures and obtains written informed consent. For illiterate women, an impartial witness attests to consent and the woman provides a thumbprint.

To encourage early presentation at ANC clinics, community sensitization was conducted prior to study initiation. Study field data collectors and community cadres disseminated information about the ENAT study at monthly community-based pregnant women's conferences, and community and religious gatherings and encouraged presentation to the health centers if/as soon as pregnancy was suspected. Study enrollment began in August 2020 and will continue until the sample size has been met. As of September 1, 2021 ENAT has enrolled 2,148 women.

Inclusion criteria:

- ≤24 weeks gestation based on a clinical algorithm (LMP and/or symphysis fundal height) who have a viable pregnancy

Exclusion criteria:

https://mc.manuscriptcentral.com/bmjpo
Confidential: For Review Only

- Pregnant women presenting for first ANC >24 weeks
- Pregnant women who live >2 hours walking distance from ENAT health centers
- Pregnant women presenting at first ANC with fetus that is non-viable (without a heartbeat on enrollment ultrasound)

ENAT Study Interventions

Health systems strengthening of ANC services was performed in ENAT health centers prior to the study to benefit all women receiving care in these facilities. Health systems strengthening was conducted in partnership with Amhara Regional Health Bureau and local partners including Jhpiego. Staff were trained in ANC standards, guidelines, and measurements (blood pressure, gestational age, birth weight [33]). Facilities were stocked with basic equipment, medications, and diagnostic testing. Laboratory capacity was augmented, and all health centers were equipped with ultrasound machines.

A. Enhanced Nutrition Package (ENP) (Table 1)

Table 1: Enhanced Nutrition Package Components

ENP Content	Activity	Frequency
Nutritional education/counselling	Counselling about healthy eating, adequate pregnancy weight gain, increasing protein and energy in diet, importance of Iron/folate, iodized salt	Every ANC visit (monthly, potentially less frequently pending COVID)
Iron-Folic Acid	Strengthen counseling, supply and reinforcement of daily IFA (60 mg Fe/400 μg folic acid)	Provide 90-day supply per FMOH, adherence/counseling at every ANC visit
Iodized salt	For all pregnant women, provide up to 1kg at enrollment and follow up visit for household use during pregnancy with counselling	Provided adequately iodized salt at enrollment, and monthly follow up visits; adherence/counseling every ANC visit
Balanced Energy Protein supplement	For pregnant women with MUAC <23 cm, local Corn Soya Blend 200 gm daily supplement (784 kcal/day, 28 g protein)	Daily supplement, distributed at ANC visits (monthly, given COVID may provide longer term supply)

A.1 Nutritional Education/Counseling on Adequate Pregnancy Nutrition and Weight Gain:

Routine ANC nutrition counseling includes increasing intake and dietary diversity, however, can vary depending on patient load. For ENP centers, supplementary, locally contextualized, counseling was developed based on our formative work [32], and is delivered by midwives. Content includes BMI-based recommended weight gain, dietary diversity, and educational messages developed to address local cultural beliefs related to dietary intake during pregnancy

Confidential: For Review Only

as well as side effects of iron-folate. Nutrition education materials, including posters and videos, are shown in ENP health centers to promote women's behavior change and maximize their exposure to various but consistent nutrition messages.

A.2 Iron Folic Acid (IFA): The Ethiopian FMOH recommends 60 mg iron plus 400 µg folic acid supplements, orally once daily in pregnancy. In August-Sept 2019, coverage of IFA was 46.3% in the ENAT health centers, and our formative work indicated that barriers included local beliefs about "big babies" and side effects such as constipation. In the ENP health centers, additional counseling is conducted, utilizing video/media to address common cultural beliefs and side effects, and women are reminded at each ANC visit about IFA consumption, management of side effects and provided refills per FMOH guidelines.

A.3 Provision of Adequately Iodized Household Salt: In ENP centers, we provide a monthly household supply of high-quality adequately iodized salt at every ANC visit. The iodized salt (Waff Manufacturing, 30-40 ppm potassium iodate, 600 gm bottle) is packaged in airtight, resealable, polyethylene containers, to allow resealing after use and to reduce risk of evaporative losses at the household level. Quality control procedures are in place to ensure that iodization is in the proper range at production and maintained at distribution sites. Women are counselled that salt should be used to replace their household salt, on the approximate daily use (~10 gm (3 pinches)/day), the proper storage of salt (away from light, heat, humidity, re-capping container after use) and use of salt only after cooking/heating of food.

A.4 Fortified Balanced Energy Protein (BEP) Supplement for Malnourished Women (MUAC <23 cm): In the ENP health centers, women who have MUAC <23 cm at enrollment, or at any follow up ANC visit, are provided with a monthly supply of locally produced micronutrient fortified corn soya flour blend (Super Cereal, Faffa Food Share Company, Addis Ababa, Ethiopia) at every ANC visit until delivery. The daily corn soya blend (CSB) supplement (200gm) is provided in addition to normal meals and contains 28 g of protein and 784 kcal (Web Table 2). This protein composition falls within recommendations that a BEP supplement provide ~50% of the additional protein requirement in the 3rd trimester (range of 28-36g for malnourished populations) [34]. Micronutrient composition of the provided BEP is also shown in Web Table 2. The fortified BEP supplement meets the IOM recommended levels in pregnancy [35] for Vitamins A, D, E, B_2, B_3, B_6, B_{12}, C, calcium and phosphorus (see Web Table 2). 35 sachets (200 gm each) are distributed to women at enrollment and at follow up monthly ANC visits to

Confidential: For Review Only

allow for additional doses in case she is delayed in returning for ANC and/or for potential family sharing practices.

B. Enhanced Infection Management Package (EIMP) (Table 2)

Women randomised to the EIMP intervention, receive the following interventions at their first ANC visit >12 weeks.

B.1 Urinary Tract Infection/Asymptomatic Bacteriuria: A clean catch midstream urine specimen is collected using a vacutainer with boric acid preservative (Beckton Dickinson). Urine culture and antibiotic susceptibility testing are performed at Amhara Public Health Institute (APHI), the regional laboratory certified by the ENAO (Ethiopian National Accreditation Office) - ISO (International Organization for Standardization) 15189. Antibiotic susceptibility is determined including the Vitek method (bioMerieux, Marcy l'Etoile, France), or Kirby Bauer Disk Diffusion. Urinary tract infections (UTI) are classified in Web Table 3 and treated with an oral antibiotic based on antibiotic sensitivity patterns (Web Table 4). Antibiotics are provided to pregnant women at no cost and the first dose is directly observed. Women with severe illness or difficult to treat infections are referred to the obstetrics department at Feligot Hiwot hospital. Women provide a test of cure specimens at the following ANC visit.

Table 2. ENAT EIMP Components

**Infection**	**ENAT Enhanced Infection Management Package (EIMP) Activity**
Urinary Tract Infection/Asymptomatic Bacteriuria	*Screen:* Urine culture and antibiotic sensitivity.<br>*Treat:* Initially per clinical protocol, with targeted antibiotic treatment based on antibiotic resistance patterns.
Sexually Transmitted/ Reproductive Tract Infections	*Screen:*<br>1) **ALL pregnant women** for gonorrhea, chlamydia using accurate rapid diagnostic testing for chlamydia and gonorrhea<br>2) Pregnant women ***with symptoms*** are screened for trichomonas and bacterial vaginosis<br>*Treat:* All positive cases and partners (for gonorrhea, chlamydia, trichomonas)
Geo-helminths	Presumptive deworming with mebendazole 500 mg or albendazole 400 mg as per FMOH guideline; Stool screen and treatment for parasitic infections at least 4 weeks after deworming. If positive, treatment for intestinal parasites per FMOH guidelines.

Confidential: For Review Only

B.2 Sexually Transmitted/Reproductive Tract Infections: Women self-collect vaginal specimens that are tested for gonorrhea and chlamydia using the *Xpert® CT/NG assay* (*Cepheid*, Sunnyvale, CA), a modular cartridge-based platform for testing each specimen by nucleic acid amplification at APHI. Chlamydia is treated with Azithromycin 1 gram orally once and gonorrhea is treated with Ceftriaxone 250 mg IM once + Azithromycin 1gram orally once. Partner treatment is on a voluntary basis with a regimen as recommended by the Ethiopian STI management guidelines [36]. A test of cure is obtained at the next ANC visit.

For women who report symptoms of abnormal vaginal discharge, vulvar symptoms, or lower abdominal tenderness, additional vaginal swabs are collected for trichomonas and bacterial vaginosis by point of care diagnostics. Trichomonas is tested using the OSOM® trichomonas rapid test (Sekisui Diagnostics, Lexington, MA). Bacterial vaginosis is tested using Diagnosit® BVBLUE® test (Gryphus Diagnostics, Knoxville, TN)). Trichomonas is treated with Metronidazole 2 grams orally once, and partners are treated. Bacterial vaginosis is treated with metronidazole 500 mg twice daily for 7 days.

B.3. Parasitic Intestinal Infections: In settings of high geo-helminth burden, WHO recommends prophylactic deworming in 2nd and 3rd trimester of pregnancy [37]. At study initiation, mebendazole (500 mg) was provided twice in pregnancy consistent with WHO guidelines. Due to health provider concerns regarding medication package insert information contraindicating use in early pregnancy, in September 2020, the protocol was modified to a single presumptive deworming in the 3rd trimester. With the adoption of new Ethiopian FMOH ANC guidelines allowing earlier provision of anti-helminthics in pregnancy, in May 2021, the ENAT protocol was modified to provide presumptive deworming in the 2nd trimester followed by a stool screening and treatment at least 4 weeks later. In the first post-deworming visit, stool is screened for intestinal parasites in the health center lab using wet mount microscopy available at the health center. Women identified with parasitic infections are treated as per FMOH recommendations (Web Table 4).

Randomisation/Allocation

At the first level of randomisation, clusters (i.e., health centers) are randomised into one of two nutrition interventions: a) ENP or b) standard nutrition care. We performed a constrained randomisation to ensure balance across the two arms of the study for key

Confidential: For Review Only

indicators including: population size, pre-study ANC coverage rates, number of births, and travel time to the regional center of Bahir Dar. We: 1) set reasonable tolerance levels for the restriction variables, 2) created all possible random sequences, where each sequence allocated 6 health centers to the ENAT Nutrition Package and 6 health centers to routine care, 3) assessed each sequence as to whether or not it met these restriction criteria, and 4) choose randomly from the subset of all such allocation sequences that met the criteria. At the second level of randomisation, we randomised individual pregnant women presenting for ANC at each health center to receive a) *ENAT Enhanced Infection Management Package (EIMP)*, or b) standard infection care (Figure 2). Each health center received a pre-generated randomisation list of sequential individual assignments to EIMP or standard care, where assignments were equally allocated to each arm within randomly permuted blocks of size 4, 8, or 12. The randomisation lists were generated separately, by health center, using a script written by one of the authors (LCM) in R [38].

Outcome Measures

The **primary outcomes** are

P1. Newborn weight measured within 72 hours of birth
P2. Newborn length measured within 72 hours of birth

The **secondary outcomes** include:

S1. Length of gestation, with gestational age determined by <24-week pregnancy ultrasonography
S2. Proportion of pregnancies resulting in spontaneous preterm delivery
S3. Proportion of newborns born of low birthweight (<2500 grams), as measured within 72 hours of life
S4. Proportion of newborns born small-for-gestational age, as defined by the INTERGROWTH 21st neonatal birth weight standard.
S5. Stillbirth rate
S6. Newborn head circumference within 72 hours of birth
S7. Infant Z-scores for weight-for-age, length-for-age, head circumference-for-age within 72 hours of birth
S8. Maternal gestational weight gain
S9. Maternal anemia (3rd trimester)

The definitions used for each outcome measure is shown in Table 3.

Confidential: For Review Only

Table 3: ENAT Study Outcomes

**Primary outcomes**	
**P1. Newborn weight <72 hours of birth**	Weight of the unclothed infant measured at <72 hours of life
**P2. Newborn length measured at birth**	Infant Length measured at <72 hours of life
**Secondary outcomes**	
**S1. Gestational age**	Gestational age determined by enrollment ultrasound, CRL used if <95 mm (Intergrowth 21st), then BPD/FL (WHO Kiserud) used if CRL>95 mm or missing
**S2a. Proportion of pregnancies resulting in preterm delivery**	Numerator: number of pregnancies resulting in spontaneous termination of pregnancy from 24 to <37 weeks (including preterm live birth or fetal loss (spontaneous pregnancy loss 24 to <37 weeks, not due to induced abortion)).<br>Denominator: All pregnancy outcomes ≥24 weeks
**S2b. Preterm live birth**	Numerator: Live births <37 weeks of gestation<br>Denominator: All Livebirths
**S3. Small for Gestational Age[a] (Intergrowth)**	<10% birthweight for GA by sex compared to Intergrowth reference[2]
**S4. Low birthweight rate**	Low birthweight is defined as birthweight (measured within the first 72 h of life) of <2500 g. We will also assess the outcome of birthweight <2000 g.
**S5. Stillbirth rate**	Numerator: Stillbirth (≥28 weeks gestation)- fetal death with no signs of life<br>*A preterm stillbirth is defined as an infant born without signs of life (no spontaneous crying, breathing, and/or movement) at 28 to <37 weeks gestation.*<br>*A term stillbirth is defined as an infant born without signs of life at ≥37 wks*<br><br>Denominator: All live births and stillbirths ≥28 weeks
**S6. Newborn head circumference**	Head circumference of the infant measured at <72 hours of age
**S7. Newborn weight, length, and head circumference for age Z-scores**	Infant weight, length, and head circumference for age z-scores measured at <72 hours of life, calculated using the Intergrowth reference for size at birth.
**S8. Rate of weight gain in pregnancy**	Maternal weight gain (kg) per week gestation in the 2nd and 3rd trimester
**S9. Maternal anemia**	Mean hemoglobin concentration in 3rd trimester<br>(Mission hemoglobinometer)

[a]Abbreviations: WHO, World Health Organization; UTI, urinary tract infection; ANC, antenatal care; SGA, small-for-gestational-age; GA, gestational age

Data Collection

The timeline of individual participant study visits, measurements and data collection are shown in Table 4. All study visits are conducted at the health center, with the exception of the birth visit

Confidential: For Review Only

that may be conducted at home within 72 hours of delivery, for births occurring at home or outside of the study area. Adherence monitoring visits also occur at the home for those participants who do not return to the health center for follow up.

The core of the data collection system is the Survey Solutions platform (World Bank, v20.08, 2021). Study nurses enter data directly into electronic tablets with programmed validity checks during study visits. Paper forms are used if tablets are temporarily unavailable. The tablets are regularly synchronized to the server on the ACIPH campus. A web-based dashboard supports data collectors, supervisors, and investigators in real time management and monitoring of study activities.

Confidential: For Review Only

Table 4. Participant timeline schedule of enrolment, interventions, assessment, and visits

	STUDY PERIOD						
		Post-allocation					
	Allocation	Prenatal				Postnatal	
**TIMEPOINT**	**Enrolment <24 weeks**	***ANC1***	***ANC2***	***ANC3***	***etc.***	***Birth***	***1 month***
**ENROLMENT:**							
**Eligibility screen**	X						
**Informed consent**	X						
**Allocation**	X						
**INTERVENTIONS:**							
**ENP**	X	X	X	X	X		
**EIMP**	X	X	X	X	X		
**ENP + EIMP**	X	X	X	X	X		
**ASSESSMENTS:**							
MOTHERS							
US (fetal growth & GA determination)	X			X			
Basic medical & obstetric history	X						
Socioeconomic status	X						
Health care costs	X		X				
Food insecurity and Dietary Intake	X	X		X			
Maternal stress and depression		X		X			X
Maternal anthropometrics	X	X	X	X	etc.	X	X
Maternal morbidity	X	X	X	X	etc.	X	X
Labor and Delivery characteristics						X	
Assessment of home environment						X	
INFANTS							
Anthropometrics						X	X
Breast feeding practices						X	X
Morbidity and mortality						X	X

Confidential: For Review O

Enrollment Visit

At the enrollment visit at the health center, data is collected on the participant's socio-economic status, basic medical and obstetric history, pregnancy history, maternal morbidity including COVID-19, food security, and dietary intake. A dietary quality questionnaire is administered, which has been used in the Ethiopian context [39].

A basic abdominal obstetric ultrasound is performed by a trained research nurse at the enrollment visit for pregnancy dating. An intensive ultrasonography training and standardization was performed by General Electric, Ethiopian Radiography Association and sonographers from Beth Israel Deaconess Hospital (Boston, MA). Sonographers measure crown-rump length, bi-parietal diameter (outer to inner), head circumference, femoral length, abdominal circumference in duplicate. Approximately 10% of images are externally reviewed (MS, BJW) for quality control.

Maternal and infant anthropometrics are measured by research staff (nurses, data collectors) at baseline and follow up visits (Table 3). Maternal weight is measured with a digital scale (ADE M317600, Germany; precision 100 gm) and height is measured using an adult stadiometer (Shorr Productions HeightLite). All measurements are performed twice, with a third measurement done if the difference is greater than the minimal acceptable difference defined by Intergrowth 21 [40]. Nurses and data collectors are trained and standardized in anthropometric measurements at the start of the study and every 6 months.

Follow up ANC Visits

During the follow up ANC visits at the health center, research staff interview women about their health status, morbidity, pregnancy history/complications, counselling/services received, maternal mental health screen, and dietary intake. Data is abstracted from routine ANC records, including blood pressure, lab testing results, and management. Maternal weight and mid-upper arm circumference are measured. In a subset of women, a semi-quantitative food frequency questionnaire for ~70 food items is administered at ANC visits. Hemoglobin is measured during enrollment in the 1st or 2nd and 3rd trimester ANC visits (Hemocue 301c). Venous hemoglobin is measured if blood is already being drawn for other purposes, and otherwise capillary hemoglobin is measured.

Confidential: For Review Only

Adherence to each nutritional supplement is assessed at every ANC visit. The participant is asked to bring the used IFA bottle, salt container, and BEP sachets back to the health center at each ANC visit. Pill or empty sachet count is done, and the salt container weighed. The participant is also asked to recall the number of sachets and/or pills that were taken in the last 7 days, and since the last visit. For mothers who do not attend scheduled ANC visits, a home visit is made by a data collector to assess adherence and conduct pill/sachet counts and remind the mother to return for ANC and study visits.

A repeat ultrasound is performed in the 3rd trimester to monitor fetal growth and assess the position of the baby. If the fetus is determined to be in non-vertex positioning at the 3rd trimester scan, the nurse recommends that the women deliver in the nearest hospital with Cesarean section capacity.

Birth Visit

Participants who deliver in health facilities are assessed by research staff based in health centers or hospitals as soon as possible after birth, but within 72 hours of life. Data is gathered from women and from chart review about the delivery and immediate postpartum period. Medical records are reviewed for intrapartum course (e.g., vital signs, duration of labor), delivery complications, and maternal/neonatal morbidity. For deliveries that occur at home, a home visit is made by research staff as soon as possible upon birth notification (within 72 hours). Maternal history is obtained per self-report regarding delivery history/complications, and postpartum maternal/neonatal morbidity.

Infant weight is measured using a high quality, precise digital infant scale (ADE M112600, Germany; precision 5 gm). Infant length is measured using a portable infantometer (Perspective Enterprises PE-RILB-LTWT, Michigan USA, precision 1 mm). Recumbent length is recorded to the last completed (not the nearest) mm. Head, chest, and mid upper arm circumferences (MUAC) is measured to the nearest millimeter (mm) using insertion tapes (Shorr productions, Maryland USA). Daily calibration checks are made before each use of infant weighing scales, and length boards to ensure accuracy of measurement [41,42].

Postnatal Visit

Confidential: For Review Only

Postnatal visits are made at 4-6 weeks for all participants to collect data on maternal and infant vital status, health, morbidity and anthropometrics. Visits are conducted primarily at the health center, and home visits are made for those who do not return for follow up. For infants who follow up at the health center for routine postnatal care or immunizations, study visits are additionally conducted at 3 and 6 months.

Cost Data

Data regarding the costs of delivery of the ENAT study interventions is collected in all study arms. Costs of interventions include three components: system-level costs, costs incurred by health workers for participating in the interventions, and costs incurred by individual patients and families. At the system level, costs are captured using the World Health Organization (WHO) framework [43] using modified survey tools based on our published survey instruments, cost estimation protocols, and procedures that have been validated and used in other LMICs (e.g., Rwanda, India) [44–46]; Costs incurred by health workers for receiving training include time or money spent for participating in training sessions. For all time spent, monetary value is assigned based on their average hourly wage. Costs incurred by patients include costs or time spent for received care or home visits. For visits in health facilities, we collect self-reported cost data from patients.

Biospecimens: In a subset of consenting women, additional biospecimens will be collected for future analysis. These specimens are shown in Web Table 5.

Statistical Analysis

Data collected in this study will enable us to conduct a comprehensive analysis of the impact of interventions randomized at the health center and individual level. The Detailed Statistical Analysis Plan (SAP) of the ENAT study is published separately at: https://addiscontinental.edu.et/. In brief, our statistical approach will have multiple steps. We will describe the health centers and pregnant women enrolled in the study and conduct a descriptive quantitative analysis of variables at multiple levels to assess the degree to which our randomization scheme resulted in similar sub-populations of pregnant women. We will assess the receipt of and adherence to interventions offered, conduct descriptive analyses of the primary and secondary outcomes, compare the outcomes between intervention groups, assess potential effect modifiers, and conduct pre-specified sub-group analysis.

Confidential: For Review Only

Sample Size

We have estimated the effect size detectable with 80% power under a cluster-randomised design, with 6 health centers per study arm. Fixing recruitment of pregnant women to 18 months, we estimated that within this time period the average health center in the proposed study site would enroll around 200 women into ANC at ≤24 weeks gestation, and would yield 112 live born infants weighed within 72 hours of life (assuming ~70% of enrolled pregnancies result in a live birth, and ~80% are followed up and weighed within 72 hours). Beyond the above determination of average cluster size, we have additionally made the following assumptions in order to estimate effect sizes detectable with 80% power: 1) mean birthweight and standard deviation as per prior studies in Gondor (mean birth weight of 2900 gm, SD 450gm) and 2) variation in distribution of weight between clusters as reflected through an coefficient of variation (k=0.01) [47]. In total this includes 2,400 pregnant mothers enrolled in 12 health centers, resulting in 1,440 live births with a birthweight within 72 hours. This sample size provides 80% power to detect a 66 gram difference in birthweight between the ENAT EIMP or routine care group in a marginal analysis (i.e. irrespective of whether mothers did or did not receive the ENP), and a 90 gm difference between ENP vs routine care (marginal analysis).

Length. With the assumptions of clusters and enrollment above, we assumed mean infant length of 49.5 cm (SD 2.4) (based on the Malawi LAIS study [48,49] and coefficient of variation k=0.008 (sector level variation in JiVitA study) [47]. For the EIMP vs. routine care comparison, we would have 80% power to detect a 3.0 mm difference in mean infant length. For the ENP vs. non-ENP comparison (marginal analysis) we would have 80% power to detect a 7.8 mm difference in infant length between the women receiving the package of enhanced nutrition-infection compared to standard nutrition care.

Study Monitoring

An external Study Monitoring Committee (SMC) is established to monitor the progress of the study, including enrollment, progress indicators and adverse events. The SMC includes an independent Ethiopian obstetrician (Dr. Delayehu Bekele) and an epidemiologist (Professor Simon Cousens). The committee met before study initiation, at mid-enrollment, and every 6 months to review study progress. Interim analysis will not be performed.

https://mc.manuscriptcentral.com/bmjpo
Confidential: For Review Only

ETHICS AND DISSEMINATION

Research Ethics approval: The ENAT protocol was approved at Addis Continental Institute of Public Health (001-A1-2019) and Mass General Brigham Institutional Review Board (2018P002479). The APHI granted local permission to conduct the study.
The study is registered at ISRCTN (ISRCTN15116516). Protocol modifications will be communicated to respective IRBs and ISRCTN.

Confidentiality

All the data collected for this study is kept strictly confidential at Addis Continental Institute of Public Health on a local encrypted server. Personal identifiers are not used in study-specific forms, aside from the identifier module. Paper copies of data forms for data entry and analysis are stored in a locked file when not in use. Access to data files containing personal identifying information is limited to the principal investigators and key staff.

Ancillary care

ENAT project helps cover ancillary care related to study participation that is not covered by the health system.

Dissemination plan

ENAT study findings will be disseminated to FMoH, APHI, ARHB, Zonal health departments, woreda health offices, community representatives, and to other relevant stakeholders. Involving relevant stakeholders in the dissemination process will help to enhance ownership of the research output and the ultimate integration of findings into programs. The dissemination will be in the form of presentations in workshops, conferences, and symposiums at local, regional, national, and international levels as appropriate. In addition, reports and peer-review journal publications will be produced.

DISCUSSION

Despite the high global burden of low birthweight, preterm birth and fetal growth restriction, few interventions have demonstrated efficacy or effectiveness in the prevention of these adverse birth outcomes. Novel, integrated approaches are needed to make more substantial, scalable and sustainable impacts in order to meet the WHO's Nutrition targets. A critical feature of the ENAT study is its health systems approach and involvement of key local stakeholders in the design of intervention packages and implementation. The packaging/bundling of interventions

https://mc.manuscriptcentral.com/bmjpo

aims to maximize their potential impact, cost-effectiveness, learning, and future scalability. This study will provide important evidence on the role of strengthening ANC programs to improve maternal health and birth outcomes, and will help inform future ANC policies in Ethiopia. ENAT will contribute to the growing body of knowledge regarding the effectiveness of BEP supplementation during pregnancy. Secondly, despite the high burden of antenatal infections, there is limited high-quality evidence that interventions to treat prenatal infections improves birth outcomes. To our knowledge, there is even more limited data on the interactions of nutrition and infections in pregnancy and this study will provide novel new insight on the potential for synergistic benefits of an integrated approach. Finally, the study will generate implementation learning on how to optimize delivery of WHO guidelines in resource limited health systems.

Access to data

The final dataset will be available to the ENAT investigative team and collaborators. Anonymized study data may be made available upon request to the study PIs in accordance with Ethiopian and US regulatory guidelines.

Declaration of interests

Financial and competing interests of the PIs

ACL reports funding from the Bill & Melinda Gates Foundation, Eunice Kennedy Shriver National Institute of Child Health and Human Development and the World Health Organization. YB reports funding from FORMAS, USAID, National Institute of Environmental Health Sciences and from the Bill & Melinda Gates Foundation. BJW reports funding from National Institute of Environmental Health Sciences and from the Bill & Melinda Gates Foundation.

Funding Statement: This work was supported by the Bill & Melinda Gates Foundation grant number OPP1184363. The funder provided input on the study design, but played no role in the data collection, data analysis, interpretation of data, writing of the report or decision to submit the report for publication.

Acknowledgements

We would like to thank the ENAT partners for their support and contributions to the study, including the Amhara Regional Health Bureau, Amhara Public Health Institute, Ethiopian Federal Ministry of Health, Ethiopian Society of Obstetrics and Gynecology, General Electric, Ethiopian Society of Radiology, and Jhpiego. We acknowledge the Child Investment Fund who supported Jhpiego health systems strengthening efforts in study areas. We would also like to thank our Study Monitoring Committee, Dr. Delayehu Bekele and Professor Simon Cousens.

Author Contributions

Wrote manuscript: ACL, LCM, FW, ME, YB

Designed study/protocol: ACL, FW, LCM, EB, ME, GC, PC, SI, CL, RM, AWT, BJW, AQ, YB

Confidential: For Review Only

Designed study materials/study tools: ACL, FW, EB, YB, MD, ME, NF, IO, WK, TS, FS, FT, ST, KY, SI, YK, CL, RM, MS, FV, AWT, YB
Trained field data collectors: FW, EB, YB, MD, ME, WK, TS, FS, FT, ST, KY, AWT, ACL, YB
Designed Data management system: NF, FS, FT, FV, AW
Trained ultrasonography: MS, BW, KY, ST
Critically reviewed/revised manuscript: all authors
All authors have met ICJME criteria.

FIGURE CAPTIONS

Figure 1. Conceptual diagram showing the pathways that link maternal undernutrition, maternal infection, and infant outcomes (A, B, and C represent mechanisms, which are described in detail in the associated sections above)

Figure 2. ENAT Study Consort Diagram

Figure 3. ENAT Study Site Map, Amhara Region, Ethiopia

Confidential: For Review Only

REFERENCES

1 World Health Organization. Global nutrition targets 2025: low birth weight policy brief. Geneva, Switzerland: 2014.

2 Blencowe H, Krasevec J, de Onis M, *et al.* National, regional, and worldwide estimates of low birthweight in 2015, with trends from 2000: a systematic analysis. *Lancet Glob Health* 2019;**7**:e849–60. doi:10.1016/S2214-109X(18)30565-5

3 Katz J, Lee AC, Kozuki N, *et al.* Mortality risk in preterm and small-for-gestational-age infants in low-income and middle-income countries: a pooled country analysis. *Lancet* 2013;**382**:417–25. doi:10.1016/S0140-6736(13)60993-9

4 Christian P, Lee SE, Donahue Angel M, *et al.* Risk of childhood undernutrition related to small-for-gestational age and preterm birth in low- and middle-income countries. *Int J Epidemiol* 2013;**42**:1340–55. doi:10.1093/ije/dyt109

5 Blencowe H, Lee ACC, Cousens S, *et al.* Preterm birth-associated neurodevelopmental impairment estimates at regional and global levels for 2010. *Pediatr Res* 2013;**74 Suppl 1**:17–34. doi:10.1038/pr.2013.204

6 Murray E, Fernandes M, Fazel M, *et al.* Differential effect of intrauterine growth restriction on childhood neurodevelopment: a systematic review. *BJOG* 2015;**122**:1062–72. doi:10.1111/1471-0528.13435

7 Danaei G, Andrews KG, Sudfeld CR, *et al.* Risk factors for childhood stunting in 137 developing countries: A comparative risk assessment analysis at global, regional, and country levels. *PLoS Med* 2016;**13**:e1002164. doi:10.1371/journal.pmed.1002164

8 Han Z, Mulla S, Beyene J, *et al.* Maternal underweight and the risk of preterm birth and low birth weight: a systematic review and meta-analyses. *Int J Epidemiol* 2011;**40**:65–101. doi:10.1093/ije/dyq195

9 Kozuki N, Katz J, Lee ACC, *et al.* Short Maternal Stature Increases Risk of Small-for-Gestational-Age and Preterm Births in Low- and Middle-Income Countries: Individual Participant Data Meta-Analysis and Population Attributable Fraction. *J Nutr* 2015;**145**:2542–50. doi:10.3945/jn.115.216374

10 Peña-Rosas JP, De-Regil LM, Garcia-Casal MN, *et al.* Daily oral iron supplementation during pregnancy. *Cochrane Database Syst Rev* 2015;:CD004736. doi:10.1002/14651858.CD004736.pub5

Confidential: For Review Only

11 Keats EC, Neufeld LM, Garrett GS, *et al.* Improved micronutrient status and health outcomes in low- and middle-income countries following large-scale fortification: evidence from a systematic review and meta-analysis. *Am J Clin Nutr* 2019;**109**:1696–708. doi:10.1093/ajcn/nqz023

12 Imdad A, Bhutta ZA. Maternal nutrition and birth outcomes: effect of balanced protein-energy supplementation. *Paediatr Perinat Epidemiol* 2012;**26 Suppl 1**:178–90. doi:10.1111/j.1365-3016.2012.01308.x

13 Ota E, Hori H, Mori R, *et al.* Antenatal dietary education and supplementation to increase energy and protein intake. *Cochrane Database Syst Rev* 2015;:CD000032. doi:10.1002/14651858.CD000032.pub3

14 Buppasiri P, Lumbiganon P, Thinkhamrop J, *et al.* Calcium supplementation (other than for preventing or treating hypertension) for improving pregnancy and infant outcomes. *Cochrane Database Syst Rev* 2011;:CD007079. doi:10.1002/14651858.CD007079.pub2

15 Gilbert NM, O'Brien VP, Hultgren S, *et al.* Urinary tract infection as a preventable cause of pregnancy complications: opportunities, challenges, and a global call to action. *Glob Adv Health Med* 2013;**2**:59–69. doi:10.7453/gahmj.2013.061

16 Romero R, Oyarzun E, Mazor M, *et al.* Meta-analysis of the relationship between asymptomatic bacteriuria and preterm delivery/low birth weight. *Obstet Gynecol* 1989;**73**:576–82.

17 Honkpéhèdji YJ, Adegbite BR, Zinsou JF, *et al.* Association of low birth weight and polyparasitic infection during pregnancy in Lambaréné, Gabon. *Trop Med Int Health* 2021;**26**:973–81. doi:10.1111/tmi.13591

18 Aderoba AK, Iribhogbe OI, Olagbuji BN, *et al.* Prevalence of helminth infestation during pregnancy and its association with maternal anemia and low birth weight. *Int J Gynaecol Obstet* 2015;**129**:199–202. doi:10.1016/j.ijgo.2014.12.002

19 Abioye AI, McDonald EA, Park S, *et al.* Maternal, placental and cord blood cytokines and the risk of adverse birth outcomes among pregnant women infected with Schistosoma japonicum in the Philippines. *PLoS Negl Trop Dis* 2019;**13**:e0007371. doi:10.1371/journal.pntd.0007371

20 Goldenberg RL, Hauth JC, Andrews WW. Intrauterine infection and preterm delivery. *N Engl J Med* 2000;**342**:1500–7. doi:10.1056/NEJM200005183422007

21 Christensen DL, Kapur A, Bygbjerg IC. Physiological adaption to maternal malaria and other adverse exposure: low birth weight, functional capacity, and possible metabolic disease in adult life. *Int J Gynaecol Obstet* 2011;**115 Suppl 1**:S16-9. doi:10.1016/S0020-7292(11)60006-4

Confidential: For Review Only

22 Krawinkel MB. Interaction of nutrition and infections globally: an overview. *Ann Nutr Metab* 2012;**61 Suppl 1**:39–45. doi:10.1159/000345162

23 Vohr BR, Poggi Davis E, Wanke CA, *et al.* Neurodevelopment: The Impact of Nutrition and Inflammation During Preconception and Pregnancy in Low-Resource Settings. *Pediatrics* 2017;**139**:S38–49. doi:10.1542/peds.2016-2828F

24 Kim H, Hwang JY, Ha EH, *et al.* Association of maternal folate nutrition and serum C-reactive protein concentrations with gestational age at delivery. *Eur J Clin Nutr* 2011;**65**:350–6. doi:10.1038/ejcn.2010.267

25 Kutlesic V, Brewinski Isaacs M, Freund LS, *et al.* Executive Summary: Research Gaps at the Intersection of Pediatric Neurodevelopment, Nutrition, and Inflammation in Low-Resource Settings. *Pediatrics* 2017;**139**:S1–11. doi:10.1542/peds.2016-2828C

26 Collin SM, Baggaley RF, Pittrof R, *et al.* Could a simple antenatal package combining micronutritional supplementation with presumptive treatment of infection prevent maternal deaths in sub-Saharan Africa? *BMC Pregnancy Childbirth* 2007;**7**:6. doi:10.1186/1471-2393-7-6

27 World Health Organization (WHO). WHO recommendations on antental care for a positive pregnancy experience. 2016.

28 Central Statistical Agency CSAE, Icf. Ethiopia Demographic and Health Survey 2016. Addis Ababa, Ethiopia: : CSA and ICF 2017.

29 Ethiopia Mini Demographic and Health Survey 2019: Key indicators. Rockville, Maryland, USA: EPHI and ICF: : Ethiopian Public Health Institute (EPHI) [Ethiopia] and ICF 2019.

30 Shiferaw MB, Zegeye AM, Mengistu AD. Helminth infections and practice of prevention and control measures among pregnant women attending antenatal care at Anbesame health center, Northwest Ethiopia. *BMC Res Notes* 2017;**10**:274. doi:10.1186/s13104-017-2609-6

31 Belyhun Y, Medhin G, Amberbir A, *et al.* Prevalence and risk factors for soil-transmitted helminth infection in mothers and their infants in Butajira, Ethiopia: a population based study. *BMC Public Health* 2010;**10**:21. doi:10.1186/1471-2458-10-21

32 Workneh F, Eglovitch M, Shiferaw T, *et al.* Dietary practices among pregnant women in rural amhara, ethiopia. *Curr Dev Nutr* 2021;**5**:698–698. doi:10.1093/cdn/nzab045_080

Confidential: For Review Only

33 Baye E, Abate FW, Eglovitch M, *et al.* Effect of birthweight measurement quality improvement on low birthweight prevalence in rural Ethiopia. *Popul Health Metr* 2021;**19**:35. doi:10.1186/s12963-021-00265-0

34 Members Of An Expert Consultation On Nutritious Food Supplements For Pregnant And Lactating Women. Framework and specifications for the nutritional composition of a food supplement for pregnant and lactating women (PLW) in undernourished and low income settings. *Gates Open Res* Published Online First: 2019. doi:10.21955/gatesopenres.1116379.1

35 Institute of Medicine. Dietary Reference Intakes (DRIs): Recommended Dietary Allowances and Adequate Intakes. Washington, DC: The National Academies Press

36 Federal Democratic Republic of Ethiopia Ministry of Health. National Guidelines for the Management of Sexually Transmitted Infections Using Syndromic. Addis Ababa, Ethiopia: 2015.

37 World Health Organization. Soil-transmitted helminth infections. 2020.https://www.who.int/news-room/fact-sheets/detail/soil-transmitted-helminth-infections (accessed 8 Sep2021).

38 R Core Team. *R: A language and environment for statistical computing*. Vienna, Austria: R Foundation for Statistical Computing 2021.

39 Workicho A, Belachew T, Feyissa GT, *et al.* Household dietary diversity and Animal Source Food consumption in Ethiopia: evidence from the 2011 Welfare Monitoring Survey. *BMC Public Health* 2016;**16**:1192. doi:10.1186/s12889-016-3861-8

40 International Fetal and Newborn Growth Consortium. The International Fetal and Newborn Growth Standards for the 21st Century(INTERGROWTH-21st) Study Protocol. 2009.

41 de Onis M, Onyango AW, Borghi E, *et al.* Comparison of the World Health Organization (WHO) Child Growth Standards and the National Center for Health Statistics/WHO international growth reference: implications for child health programmes. *Public Health Nutr* 2006;**9**:942–7. doi:10.1017/phn20062005

42 Bloem M. The 2006 WHO child growth standards. *BMJ* 2007;**334**:705–6. doi:10.1136/bmj.39155.658843.BE

43 The world health report 2000 - Health systems: improving performance. Geneva, Switzerland: : World Health Organization 2000.

Confidential: For Review Only

44 Lu C, Tsai S, Ruhumuriza J, *et al.* Tracking rural health facility financial data in resource-limited settings: a case study from Rwanda. *PLoS Med* 2014;**11**:e1001763. doi:10.1371/journal.pmed.1001763

45 Tsai S, Umugiraneza G, Ruhumuriza J, *et al.* Training manual on tracking financial data in health facilities in Kayonza and Kirehe. Boston, MA: : Program in Global Health Economics and Social Change, Harvard Medical School 2013.

46 Lu C, Tsai S, Ruhumuriza J, *et al.* Obtaining financing data for local health facilities in two districts of Rwanda: General guidelines. Boston, MA: : Program in Global Helath Economics and Social Change, Harvard Medical School 2013.

47 West KP, Shamim AA, Mehra S, *et al.* Effect of maternal multiple micronutrient vs iron-folic acid supplementation on infant mortality and adverse birth outcomes in rural Bangladesh: the JiVitA-3 randomized trial. *JAMA* 2014;**312**:2649–58. doi:10.1001/jama.2014.16819

48 Luntamo M, Kulmala T, Mbewe B, *et al.* Effect of repeated treatment of pregnant women with sulfadoxine-pyrimethamine and azithromycin on preterm delivery in Malawi: a randomized controlled trial. *Am J Trop Med Hyg* 2010;**83**:1212–20. doi:10.4269/ajtmh.2010.10-0264

49 Luntamo M, Kulmala T, Cheung YB, *et al.* The effect of antenatal monthly sulphadoxine-pyrimethamine, alone or with azithromycin, on foetal and neonatal growth faltering in Malawi: a randomised controlled trial. *Trop Med Int Health* 2013;**18**:386–97. doi:10.1111/tmi.12074

Confidential: For Review Only

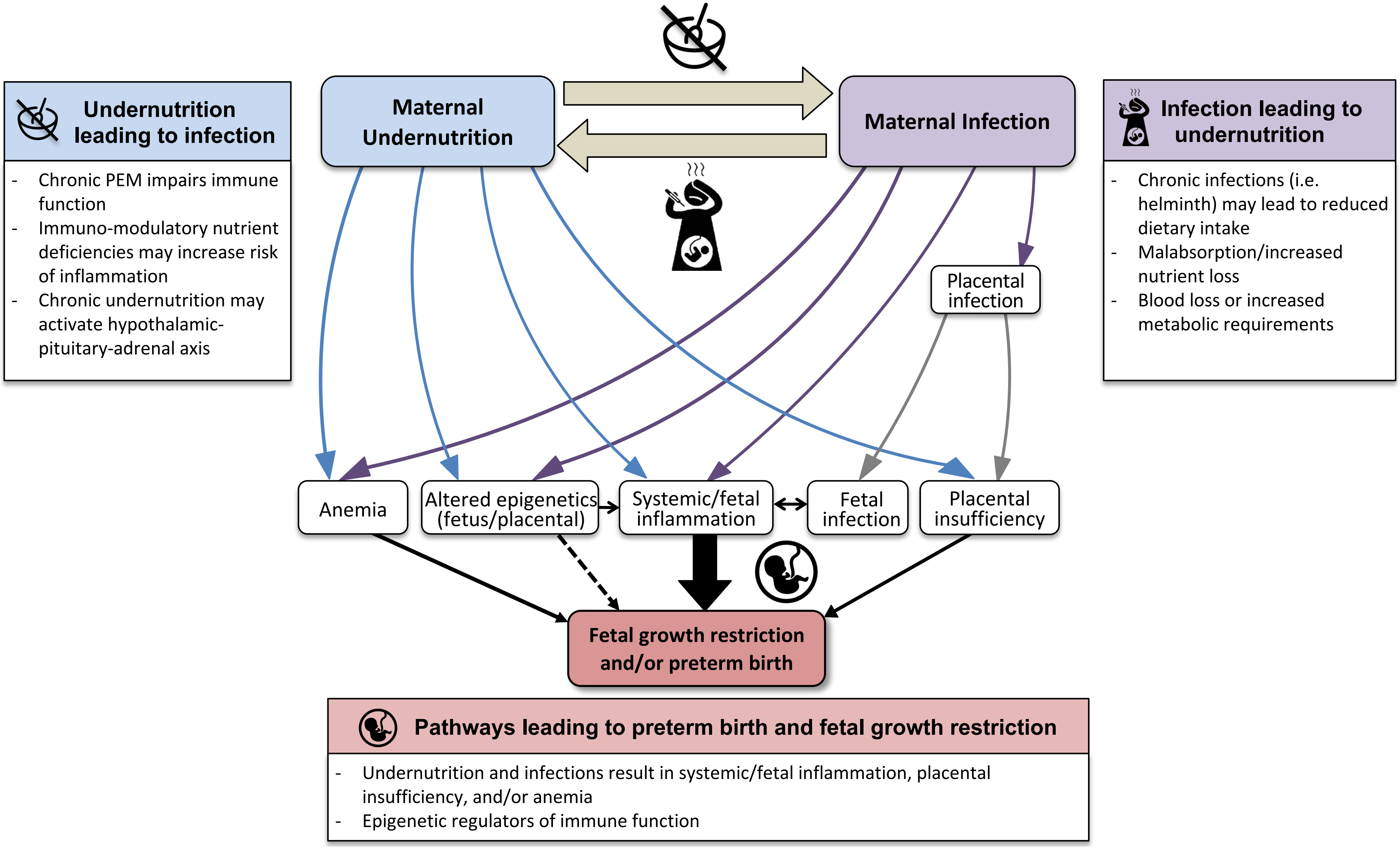
Undernutrition leading to infection
- Chronic PEM impairs immune function
- Immuno-modulatory nutrient deficiencies may increase risk of inflammation
- Chronic undernutrition may activate hypothalamic-pituitary-adrenal axis
Maternal Undernutrition
Maternal Infection
Infection leading to undernutrition
- Chronic infections (i.e. helminth) may lead to reduced dietary intake
- Malabsorption/increased nutrient loss
- Blood loss or increased metabolic requirements
Placental infection
Anemia
Altered epigenetics (fetus/placental)
Systemic/fetal inflammation
Fetal infection
Placental insufficiency
Fetal growth restriction and/or preterm birth
Pathways leading to preterm birth and fetal growth restriction
- Undernutrition and infections result in systemic/fetal inflammation, placental insufficiency, and/or anemia
- Epigenetic regulators of immune function

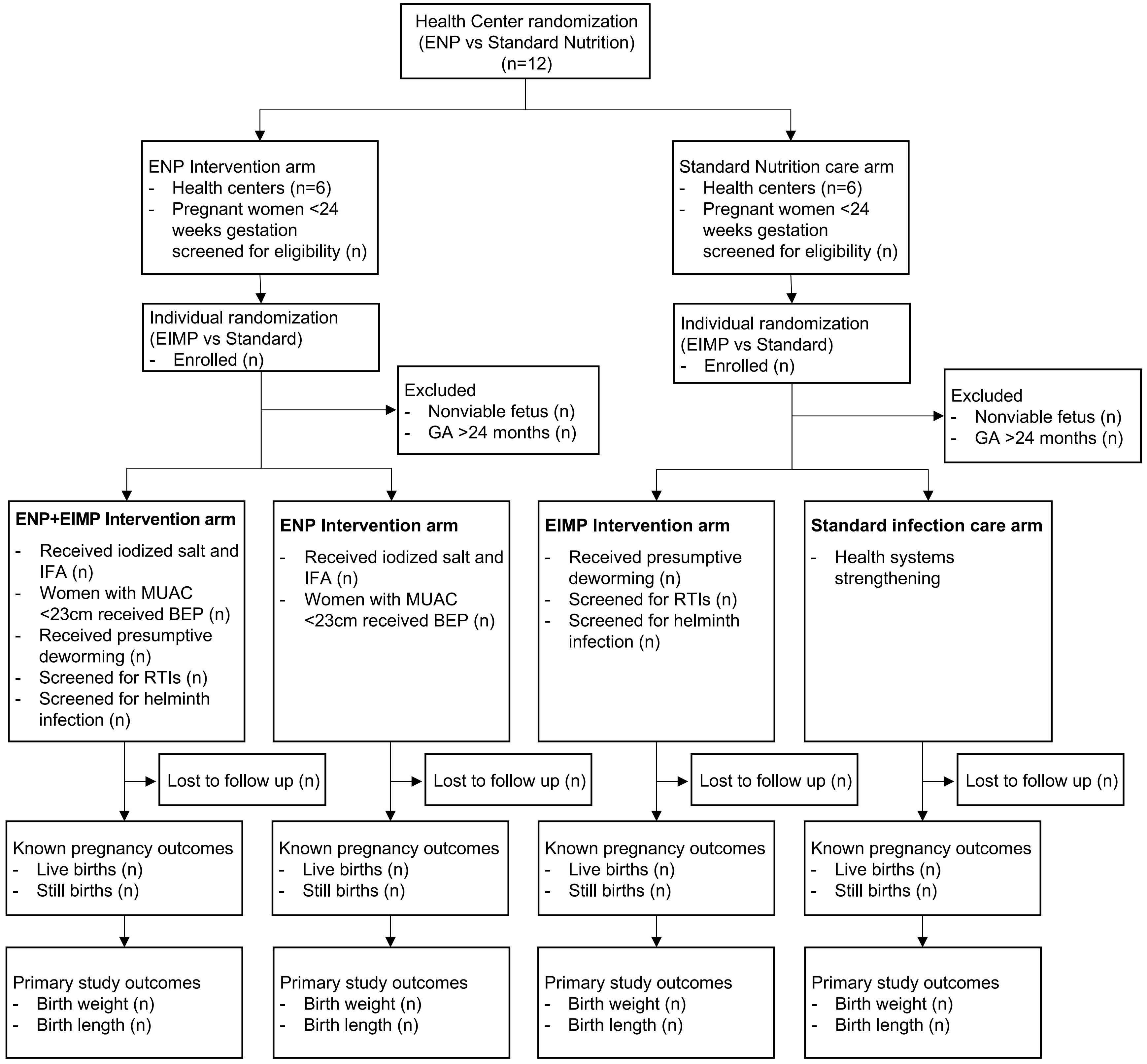
Health Center randomization (ENP vs Standard Nutrition) (n=12)
ENP Intervention arm
- Health centers (n=6)
- Pregnant women <24 weeks gestation screened for eligibility (n)
Standard Nutrition care arm
- Health centers (n=6)
- Pregnant women <24 weeks gestation screened for eligibility (n)
Individual randomization (EIMP vs Standard)
- Enrolled (n)
Individual randomization (EIMP vs Standard)
- Enrolled (n)
Excluded
- Nonviable fetus (n)
- GA >24 months (n)
Excluded
- Nonviable fetus (n)
- GA >24 months (n)
ENP+EIMP Intervention arm
- Received iodized salt and IFA (n)
- Women with MUAC <23cm received BEP (n)
- Received presumptive deworming (n)
- Screened for RTIs (n)
- Screened for helminth infection (n)
ENP Intervention arm
- Received iodized salt and IFA (n)
- Women with MUAC <23cm received BEP (n)
EIMP Intervention arm
- Received presumptive deworming (n)
- Screened for RTIs (n)
- Screened for helminth infection (n)
Standard infection care arm
- Health systems strengthening
Lost to follow up (n)
Lost to follow up (n)
Lost to follow up (n)
Lost to follow up (n)
Known pregnancy outcomes
- Live births (n)
- Still births (n)
Known pregnancy outcomes
- Live births (n)
- Still births (n)
Known pregnancy outcomes
- Live births (n)
- Still births (n)
Known pregnancy outcomes
- Live births (n)
- Still births (n)
Primary study outcomes
- Birth weight (n)
- Birth length (n)
Primary study outcomes
- Birth weight (n)
- Birth length (n)
Primary study outcomes
- Birth weight (n)
- Birth length (n)
Primary study outcomes
- Birth weight (n)
- Birth length (n)

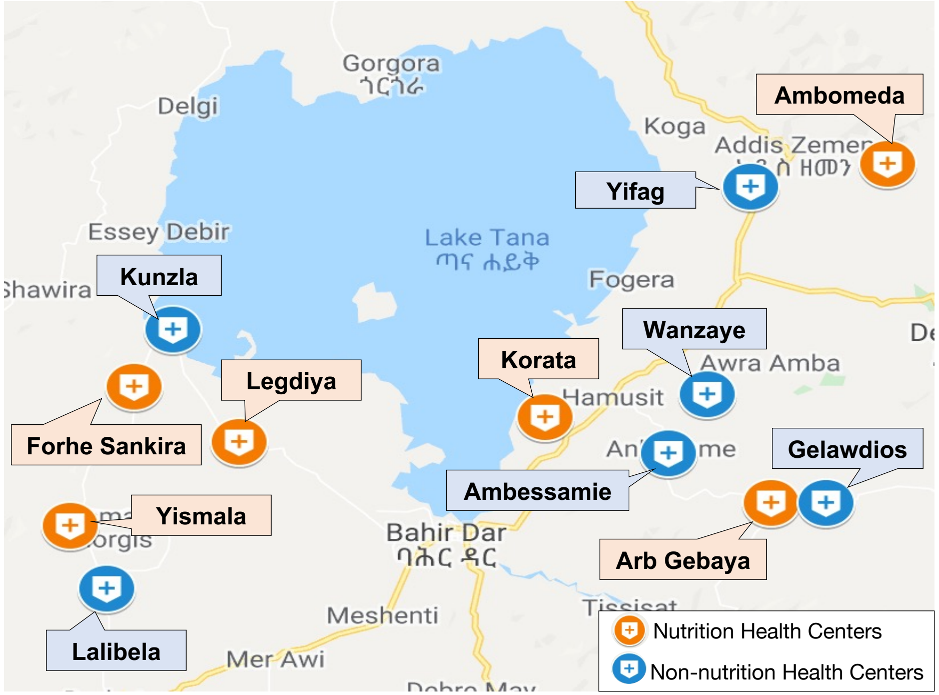
Gorgora
Delgi
Koga
Addis Zemen
Ambomeda
Yifag
Essey Debir
Lake Tana
Fogera
Kunzla
Wanzaye
Awra Amba
Korata
Legdiya
Hamusit
Forhe Sankira
Gelawdios
Ambessamie
Yismala
Bahir Dar
Arb Gebaya
Lalibela
Meshenti
Mer Awi
Nutrition Health Centers
Non-nutrition Health Centers

WEBAPPENDIX

Web Table 1. WHO Recommendations for Antenatal Care

**Nutritional Interventions**	
A.1.1: Counselling about healthy eating and keeping physically active during pregnancy is recommended for pregnant women to stay healthy and to prevent excessive weight gain during pregnancy.	Recommended
A.1.2: In undernourished populations, nutrition education on increasing daily energy and protein intake is recommended for pregnant women to reduce the risk of low-birth-weight neonates.	Context-specific recommendation
A.1.3: In undernourished populations, balanced energy and protein dietary supplementation is recommended for pregnant women to reduce the risk of stillbirths and small-for-gestational-age neonates.	Context-specific recommendation
A.2.1: Daily oral iron and folic acid supplementation with 30 mg to 60 mg of elemental iron and 400 µg (0.4 mg) of folic acid is recommended for pregnant women to prevent maternal anaemia, puerperal sepsis, low birth weight, and preterm birth.	Recommended
A.2.2: Intermittent oral iron and folic acid supplementation with 120 mg of elemental iron and 2800 µg (2.8 mg) of folic acid once weekly is recommended for pregnant women to improve maternal and neonatal outcomes if daily iron is not acceptable due to side-effects, and in populations with an anaemia prevalence among pregnant women of less than 20%.	Context-specific recommendation
A.3: In populations with low dietary calcium intake, daily calcium supplementation (1.5–2.0 g oral elemental calcium) is recommended for pregnant women to reduce the risk of pre-eclampsia.	Context-specific recommendation
A.4: Vitamin A supplementation is only recommended for pregnant women in areas where vitamin A deficiency is a severe public health problem, to prevent night blindness.	Context-specific recommendation
A.5: Zinc supplementation for pregnant women is only recommended in the context of rigorous research.	Context-specific recommendation (research)
A.10: For pregnant women with high daily caffeine intake (more than 300 mg per day), lowering daily caffeine intake during pregnancy is recommended to reduce the risk of pregnancy loss and low-birth-weight neonates.	Context-specific recommendation
**Maternal Assessment**	
B.1.1: Full blood count testing is the recommended method for diagnosing anaemia in pregnancy. In settings where full blood count testing is not available, on-site haemoglobin testing with a haemoglobinometer is recommended over the use of the haemoglobin colour scale as the method for diagnosing anaemia in pregnancy.	Context-specific recommendation
B.1.2: Midstream urine culture is the recommended method for diagnosing asymptomatic bacteriuria (ASB) in pregnancy. In settings where urine culture is not available, on-site midstream urine Gram-staining is recommended over the use of dipstick tests as the method for diagnosing ASB in pregnancy.	Context-specific recommendation
B.2.4: One ultrasound scan before 24 weeks of gestation (early ultrasound) is recommended for pregnant women to estimate gestational age, improve detection of fetal anomalies and multiple pregnancies, reduce induction of labour for post-term pregnancy, and improve a woman's pregnancy experience.	Recommended
**Preventive Measures**	
C.1: A seven-day antibiotic regimen is recommended for all pregnant women with asymptomatic bacteriuria (ASB) to prevent persistent bacteriuria, preterm birth and low birth weight.	Recommended
C.2: Antibiotic prophylaxis is only recommended to prevent recurrent urinary tract infections in pregnant women in the context of rigorous research.	Context-specific recommendation (research)
C.4: In endemic areas, preventive anthelminthic treatment is recommended for pregnant women after the first trimester as part of worm infection reduction programmes.	Context-specific recommendation

Confidential: For Review Only

Web Table 2. Super Cereal Corn Soya Blend Composition (per daily 200 gm serving)

NUTRIENT	Units	ENAT CSB CONTENT (200 gm)	Recommended Target*
Energy	Kcal	760	250-500kcal per daily serving**
Protein	gm	28	14-18 g**
Fat	%	12%	10-60% of energy
Vitamin A	mcg RE	2076	550-770***
Vitamin D	mcg	22.1	10-15
Vitamin E	mg	16.6	16-19
Vitamin K	mcg	60	72-90
Vitamin B1	mg	0.4	1.2-1.4
Vitamin B2	mg	2.8	1.3-1.6
Vitamin B3	mg	16	14-18
Folic Acid	mcg	220	400-600
Vitamin B6	mg	2.0	1.7-2.0
Vitamin C	mg	180	100-120
Calcium	mg	724	500-1500
Iron	mg	8	22-27
Iodine	mcg	80	209-290
Phosphorous	mg	560	300-700
Zinc	mg	10	15-20

* Targets are results of an Expert Consultation held at Bill and Melinda Gates Foundation.[50]
** Energy balance from macronutrient: portion size can be doubled in settings of high energy gaps, such as maternal malnutrition and where prevalence of low birthweight is high.
*** Micronutrient recommendations were primarily based upon Institute of Medicine (IOM) estimated average requirement (EAR) and recommended dietary allowances (RDA) values.

Confidential: For Review Only

Web Table 3. Classification of UTI in ENAT study

UTI Terminology	Definition
High-burden growth	bacteriuria of >10^5 colony forming units (CFU) per 1mL of urine of a single uropathogen [49],
Intermediate growth	bacteriuria with >10^3 -10^5 CFU/mL of a single uropathogen,
Contamination	bacterial growth of $\geq$3 micro-organism OR growth of a non-urinary tract pathogen.
UTI symptoms	dysuria, urinary frequency, urinary urgency, hematuria, abdominal pain, fever, OR flank pain
Symptomatic intermediate growth	women with intermediate burden growth and UTI symptoms (as above)
Asymptomatic bacteriuria	women with high burden bacterial growth without UTI symptoms
Cystitis	women with positive urine culture (high burden or intermediate growth) and symptoms of dysuria, urinary frequency, hematuria, urinary urgency or suprapubic tenderness, without upper urinary tract symptoms (fever, chills, flank or back pain) [49]
Pyelonephritis	women with positive urine culture and systemic symptoms (fever, chills, flank pain or back pain) [49].

Confidential: For Review Only

Web Table 4. Recommended Treatments for Parasitic Stool Infections FMHACA Standard Treatment Guideline,3rd Edition 2014

Intestinal parasite	Recommended treatment, and alternative
Entameoba Histolytica	Metronidazole 500 mg P.O. TID x 5-7 days
Giardia lamblia	Tinidazole 2 gm po single dose<br>Or Alternative:<br>Metronidazole 500 mg po TID x 5 days
**Ascariasis**<br><br>***Ascaris lumbricoids***	Mebendazole, 500mg P.O. once or<br>Albendazole, 400mg P.O. as a single dose<br>Or Alternative:<br>Pyrantel pamoate, 700mg P.O. as a single dose
**Hookworm infestation**<br>***Necator americanus or Ancylostoma duodenale***	Mebendazole, 500mg stat or 100mg P.O. BID for 3 days<br>Albendazole, 400mg P.O. as a single dose<br>Or Alternative:<br>Pyrantel pamoate, 700mg P.O. as a single dose
**Enterobiasis**<br><br>***Enterobius Vermicularis***	Mebendazole, 100mg P.O. BID for 3 days or<br>Or Alternative:<br>Albendazole, 400mg P.O. as a single dose
**Trichuriasis**<br>***T.tricura [Whipworm]***	Mebendazole, 500mg P.O. single dose or,<br>Or Alternative:<br>Albendazole, 400mg, P.O. for three days
**Taeniasis**<br><br>***T.saginata or T.solium***	Praziquantel P.O. 600mg or 10mg/Kg, single dose<br>Or Alternative:<br>Niclosamide, 2g in a single dose P.O.
Hymenolepis nana	Praziquantel, 25mg/kg or 1800mg P.O. single dose<br>Alternative:<br>Niclosamide, 2g P.O. on the first day followed by 1g QD for 6 days
Schistosoma Mansoni	Praziquantel P.O. 1200 mg single dose (or 600 mg po in 2 doses)
**Strongyloidiasis**<br><br>***Strongloidexs stercolaries***	Albendazole 400mg P.O. BID for three consecutive days.

Confidential: For Review Only

Web Table 5. Biological Specimens Collected in ENAT Study

A table of biospecimens and planned lab testing is presented below. Biospecimens collected for immediate analyses at the APHI laboratory are stored at room temperature using special collection containers and swabs designed to meet this storage requirement. Samples collected for future analyses are aliquoted to appropriate containers and stored at APHI for long term storage in a specially procured freezer for this purpose. All samples are stored for up to 5 years.

ENAT Bio-specimens

			Antenatal		Post-natal	
			≤24 wks	~30-34 wks	0 -<72 hrs	
Biospecimen	N (Total)	Planned lab testing	Enroll	ANCFU2	Birth	1-2 mo.
Maternal urine	800	Culture, subset of 600 for iodine concentration, selenium, environmental toxins	■	■		
Maternal vaginal swab	150	STI/RTI	■			
Maternal blood serum	700	Nutrient levels, TORCH infections	■	■		
blood spot	1000	Inflammation, thyroid, nutrients, miRNAs	■	■		
VAMS + DBS	120	metabolomics, proteomics	■	■	■	■
Maternal vaginal swab	150	Microbiome, metabolome	■	■		■
Maternal stool	120	Microbiome	■	■		■
Maternal oral sample	120	Microbiome	■	■		■
Umbilical cord blood/spot	650	Inflammation, thyroid, nutrients, miRNAs			■	
Infant blood (VAMS + DBS)	120	Metabolomics, Proteomics			■	■
Infant stool	160	Microbiome			■	■
Infant oral sample	100	Microbiome, CMV			■	■
Breast milk	100	Nutrient composition, microbiome				■

Web Form 1. Model Informed Consent Form

Information sheet and Consent: ENP Arm

Name of the organization: Addis Continental Institute of Public Health
Name of the Sponsor: Bill and Melinda Gates Foundation and Brigham and Women's Hospital

Good morning/Good afternoon.
My name is ________________________________. I am a study nurse at Addis Continental Institute of Public Health (ACIPH). ACIPH is doing the ENAT study together with the Federal Ministry of Health, Amhara Regional Health Bureau, Amhara Public Health Institute, and other partners.

Purpose: We are asking you to become part of a research study that has a goal to improve the health of the pregnant women and their babies. The main goal of the study is to see if babies will grow better in the womb if pregnant women are provided with better nutrition and are treated for infections in pregnancy. Pregnant women will be enrolled if they are less than 6 months pregnant and will be followed up to 6 months after the baby's birth. The total number of pregnant women enrolled in this study will be 3,600.

Explanation of study procedures:
We will measure your height, weight, and middle upper arm circumference. If your middle-upper arm circumference is >23 cm, you will be given iodized salt and iron folate to be consumed daily until you deliver your baby. Whenever you come to the health center, a midwife will give nutrition counseling about nutrients in IFA and iodized salt and maintaining appropriate weight gain during pregnancy for you and your baby.

If the study nurse finds that your arm size is small (middle upper arm circumference <23 cm), you will also be provided a nutritious food supplement of a corn soy blend flour. You may use the corn soy blend to make a daily serving to take on each day. These supplements will be given to you at the health center once a month until you give birth. We believe this will give the unborn baby the nutrition s/he needs to grow better.

We also hope that treating infections in pregnancy will help you to have a healthier pregnancy, increase the chances that the baby will deliver at the expected due date, and help the baby grow better in the womb.

If you decide to participate in the study, you will be put into one of two groups. The study team will choose which group you will be put into randomly. There is no way to tell which group you will be in. Neither you, the study staff, nor the health workers can choose what group you will be in. You will have an equal chance of being placed in either of the two group.

If you are assigned to the first group, you will be tested and treated for certain infections. You will be given deworming medication during the second trimester of your pregnancy, and you will be asked to provide a stool sample to screen and treat for persistent helminthiasis (worms) in the

Confidential: For Review Only

third trimester. You also be asked to give a sample of your urine to test for infection that may affect your health and the health of the baby. The study nurse and laboratory personnel will demonstrate to you how to collect the sample. The urine sample will be analyzed at APHI and you will be asked to come back to the health center within 7 days to get results from your tests.

If you are not in the first group, you will receive the routine antenatal care that is provided at the health center. You will not receive any additional testing or treatment.

After your baby is born, we will need to very carefully measure your baby's height, weight, chest, head and arm size. It is important these measurements happen within three days of giving birth. Even if you give birth at home, a trained person will visit you to carefully take them. We may also measure your baby at a later study visits at 1, 3, and 6 months.

We ask for your permission to be contacted during the study to remind you of study visits, to notify you that your test results have arrived, visit you in case of birth at home and/or to allow us to come to your house to measure the level of iodine in the salt that you use for household consumption. We will remind you about your visit by phone call or text message. If you do not respond, we may visit your home. Please show below how you agree to be contacted:

☐ Text message (to you or a family member with a phone)

☐ Phone call (to you or a family member with a phone)

☐ Home visit (If you do not come to the facility for your visit, a study staff may visit you at your home.)

Participants for these activities are pregnant women, less than 6 months pregnant, who come for ANC at health centers at the study site during the study period. If you are willing to be a part of the study, you need to understand and sign this form. All your personal information for this study will be kept private in our office where no one will be able to see it. Your identity will not be used in any reports or publications that come from the study.

Risk or discomforts: You may feel embarrassed or nervous answering some questions about your health and your baby's health. You do not have to answer every question if you do not want to.

There also maybe risk of aside effect to a medication. If you are treated for an infection, all of the medicines that will be given by your midwife are routine and considered safe to have while pregnant and are not known to hurt the baby.

We will give you our phone number so that you can call us with any concerns about bad reactions to any of the medications. We will help you to be seen and treated at the health center or nearby hospital and help pay the cost of treatment or transportation if it's absolutely necessary.

Benefits: All mothers who join in the study will get an ultrasound and at the third trimester (28 weeks of GA), that will help make sure your pregnancy is healthy. You will be screened for

Confidential: For Review Only

anemia, get iodized salt that may help you to be more nourished and help your baby grow. If you have poor nutrition, you will get a corn soy blend flour containing nutrients to eat every day. This supplement may also help improve your and your baby's nutrition during the pregnancy.

Some mothers will be tested for infections and get treatment that will help your and your baby's health. Though you may not get money or gifts, being a part of the study is very important in making programs that can help mother and child health, especially in helping babies grow and develop better in the womb.

Confidentiality and Anonymity: Information about you that we will collect by this research project will be kept. Your personal information will not be seen by anyone except the study team, and it will only be used for this research. No identities will be used in any reports or publications resulting from the study.

Right to Refuse or Withdraw: Being a part of this study and everything you are asked to do is completely voluntary. You have the full right to refuse from being a part of this study. You also have the full right to stop being a part of this study at any time you wish to. Refusing to be in the study or stopping will not affect the services that you get from the health facility.

We thank you very much for your time. If you have any questions, you can call:
Professor Yemane Berhane: Addis Continental Institute of Public Health,
Tel: +251 114 168207/ + 251 911219785 (mobile)
E-mail: yemaneberhane@addiscontinentual.edu.et OR yemaneberhane@gmail.com

Addis Continental Institute of Public Health, Tel: +251 116 390039
E-mail: aciphaddis@gmail.com

Confidential: For Review Only

Consent Form

I have read this information and decided to be a part of this study. The general purpose of study has been explained well to me and I was given the chance to ask questions. I understand that I can stop my participation and anything for the study at any time.

___________________________ ________________ _________________

Participants name Participants age ENAT Study ID

___________________________ ________________

Participants signature Date (DD/MM/YY)

___________________________ ________________ _________________

Study nurse name Study nurse signature Date (DD/MM/YY)

If illiterate:

I have seen the careful reading of the consent form to the potential participant and the participant has been able to ask questions. I agree that the participant has given consent freely.

___________________________ ________________ _________________

Witness name Witness signature Date (DD/MM/YY)

___________________________ ________________ _________________

Participants name Participants age ENAT Study ID

Thumb print of participant Date (DD/MM/YY)

___________________________ ________________ _________________

Study nurse name Study nurse signature Date (DD/MM/YY)

Confidential: For Review Only

The Enhancing Nutrition and Antenatal Infection Treatment (ENAT) Study: Protocol of a pragmatic clinical effectiveness study to improve birth outcomes in Ethiopia

Anne CC Lee[1,2*], Firehiwot Workneh Abate[3], Luke C. Mullany[4], Estifanos Baye[1], Yoseph Yemane Berhane[3], Mulatu Melese Derebe[5], Michelle Eglovitch[1], Nebiyou Fasil[3], Ingrid E Olson[1], Workagegnehu Tarekegn Kidane[3], Tigest Shiferw[3], Fisseha Shiferie[3], Fitsum Tsegaye[3], Sitota Tsegaye[3], Kalkidan Yibeltal[3], Grace J. Chan[2,6,7], Parul Christian[4], Sheila Isanaka[8], Yunhee Kang[4], Chunling Lu[2,9], Rose L. Molina[2,10], Michele D. Stojanov[10], Fred Van Dyk[4], Amare Worku Tadesse[3,11], Blair J. Wylie[2,9], Alemayehu Worku[3], Yemane Berhane[3]

[1]Department of Pediatric Newborn Medicine, Brigham and Women's Hospital, Boston, MA, USA
[2]Harvard Medical School, Boston, Massachusetts, USA
[3]Addis Continental Institute of Public Health, Addis Ababa, Ethiopia
[4] Department of International Health, Johns Hopkins Bloomberg School of Public Health, Baltimore, MD, USA.
[5]Amhara Public Health Institute, Bahir Dar, Ethiopia
[6]Department of Epidemiology, Harvard T.H. Chan School of Public Health, Boston, MA, USA
[7] Department of Medical Critical Care, Boston Children's Hospital, Harvard Medical School, Boston, Massachusetts, USA
[8]Departments of Nutrition and Global Health and Population, Harvard T.H. Chan School of Public Health, Boston, MA, USA
[9] Division of Global Health Equity, Brigham and Women's Hospital, Boston, Massachusetts, USA
[10]Department of Obstetrics and Gynecology, Beth Israel Deaconess Medical Center, Harvard Medical School, Boston, MA, USA
[11]Department of Infectious Disease Epidemiology, London School of Hygiene and Tropical Medicine, London, UK

Corresponding Author:
Anne CC Lee
75 Francis St, Boston MA 012115
617-732-8343
Alee6@bwh.harvard.edu

Word Count: 4400

Confidential: For Review Only

ABSTRACT

Introduction: The WHO Nutrition Target aims to reduce the global prevalence of low birth weight 30% by 2025. The Enhancing Nutrition and Antenatal Infection Treatment (ENAT) study will test the impact of packages of pregnancy interventions to enhance maternal nutrition and infection management on birth outcomes in rural Ethiopia.

Methods and analysis: ENAT is a pragmatic, open-label, 2x2 factorial, randomised clinical effectiveness study implemented in 12 rural health centers in Amhara, Ethiopia. Eligible pregnant women presenting at antenatal care (ANC) visits at <24 weeks gestation are enrolled (n=2400). ANC quality is strengthened across all centers. Health centers are randomised to receive an enhanced nutrition package (ENP) or standard nutrition care, and within each health center, individual women are randomised to receive an enhanced infection management package (EIMP) or standard infection care. At ENP centers, women receive a regular supply of adequately iodized salt and iron-folate (IFA), enhanced nutrition counseling, and those with mid-upper arm circumference <23 cm receive a micronutrient fortified balanced energy protein supplement (corn soya blend) until delivery. In standard nutrition centers, women receive routine counseling and IFA. EIMP women have additional screening/treatment for urinary and sexual/reproductive tract infections and intensive deworming. Non-EIMP women are managed syndromically per Ministry of Health Guidelines. Participants are followed until 1 month postpartum, and a subset until 6 months. The primary study outcomes are newborn weight and length measured at <72 hours of age. Secondary outcomes include preterm birth, low birthweight, and stillbirth rates; newborn head circumference; infant weight and length for age z-scores at birth; maternal anemia; and weight gain during pregnancy.

Ethics and dissemination: ENAT is approved by the Institutional Review Boards of Addis Continental Institute of Public Health (001-A1-2019) and Mass General Brigham (2018P002479). Results will be disseminated to local and international stakeholders.

ISRCTN Registration: 15116516

Funding: Bill & Melinda Gates Foundation

Protocol: Version 4.0 (August 2021)

Confidential: For Review Only

Article Summary

What is known about the subject:

- In low- and middle-income countries, maternal undernutrition is prevalent and a major risk factor for adverse birth outcomes, including spontaneous preterm birth, low birth weight and small for gestational age infants.
- Maternal infections in pregnancy are also common, yet under-recognized risk factors for preterm birth and poor fetal growth in low- and middle-income countries.
- Beyond their independent effects, maternal infections and nutritional status may have synergistic effects on fetal growth and gestational length.

What this study hopes to add:

- Increase the evidence-base on the role of antenatal infection management on maternal and birth outcomes in a low resource rural setting in Sub-Saharan Africa.
- Increase the evidence-base on the role of targeted, fortified balanced energy protein supplementation on maternal and birth outcomes in a low resource rural setting Sub-Saharan Africa.
- Evaluate the benefit of developing intervention packages with local stakeholders that are implemented within existing health systems
- ~~Increase the evidence-base on the role of antenatal infection management on maternal and birth outcomes in a low resource rural setting.~~
- ~~Increase the evidence-base on the role of targeted, fortified balanced energy protein supplementation on maternal and birth outcomes in a low resource rural setting.~~
- ~~Evaluate the benefit of developing intervention packages with local stakeholders that are implemented within existing health systems.~~

Confidential: For Review Only

INTRODUCTION

The World Health Organization (WHO) Third Global Nutrition Target aims to reduce the proportion of babies born low birthweight (LBW, <2500 grams) by 30% by the year 2025[1]. Approximately 20.5 million infants were born LBW in 2015, with 91% from low-middle income countries (LMIC) and 24% in Sub-Saharan Africa [2]. The main etiologies of low birthweight are preterm birth (<37 gestational weeks) and fetal growth restriction, commonly classified as small-for-gestational-age (SGA) at birth. Preterm and SGA infants carry increased risk of mortality, morbidity, childhood stunting, neurodevelopmental impairment, and adult chronic disease [3–7]. Prevention of preterm birth and SGA is a key public health strategy to improve child survival and health in LMICs.

In LMICs, maternal undernutrition is prevalent and a major risk factor for adverse birth outcomes, including spontaneous preterm birth, LBW and SGA [8,9]. Interventions to improve maternal nutritional status in pregnancy, including iron-folate [10], multiple-micronutrients [11], and balanced protein-energy (BEP) supplementation [12,13], have been individually tested and found to be effective in increasing mean birthweight. However, lower than expected benefit has been observed with the individual nutritional interventions [10,12–14]. Greater effect sizes are noted in undernourished women, and there is a need for additional studies with standardized supplementation criteria as well as the combination of BEP and micronutrient supplementation [12,13].

Maternal infections in pregnancy are also common, yet under-recognized risk factors for preterm birth and poor fetal growth in LMICs. Urinary tract infection or asymptomatic bacteriuria may affect 9-80% of pregnancies in sub-Saharan Africa [15] and are associated with a 2-fold elevated risk of preterm delivery [16]. Helminthic infections are prevalent and associated with systemic inflammation, LBW and preterm birth [17–19]. Genital tract infections may ascend the reproductive tract and lead to infection and inflammation in the amniotic fluid, predisposing to preterm birth [20]. In LMICs, screening and treatment of genitourinary tract infections during routine antenatal care (ANC) is infrequent due to lack of resources and capacity for laboratory testing. While epidemiologic data has consistently established associations between prenatal infections and adverse pregnancy outcomes, there is limited evidence on the effectiveness of prenatal interventions to screen and treat infections to prevent LBW in LMICs.

Confidential: For Review Only

Beyond their independent effects, maternal infections and nutritional status may have synergistic effects on fetal growth and gestational length [21–25]. Figure 1 depicts several pathways linking maternal nutrition and infections in pregnancy. This framework provides the basis for the hypothesis that targeting both risk factors in pregnancy may lead to more substantial, and potentially synergistic, improvements in fetal growth and pregnancy length [26].

This manuscript details the study protocol for the Enhancing Nutrition and Antenatal Infection Treatment (ENAT) study in Amhara region, Ethiopia. In 2016, WHO released recommendations on an evidence-based, core package of ANC to optimize the pregnancy experience and outcomes [27], include guidelines on nutrition and infection management in pregnancy (Web Table 1). In Ethiopia, not all recommendations have been adopted or achieved high level of coverage or quality in ANC. The primary aim of the ENAT study is to determine the impact of WHO-recommended ANC interventions to optimize maternal nutrition and manage maternal pregnancy infections on infant birth size in Amhara, Ethiopia. We hypothesize that the nutrition and infection management packages will independently increase newborn birth weight and length, and that the combined effect of both packages will be greater than either alone.

METHODS AND ANALYSIS

Study Design

The ENAT study is a 2x2 factorial pragmatic, open-label, randomised clinical effectiveness study with cluster randomisation of the enhanced nutrition package (ENP) versus standard care (non-ENP), and individual level randomisation of an enhanced infection management package (EIMP) versus standard care (non-EIMP) (Figure 2).

Study Setting

The ENAT study site was established in 2018 as a partnership with the Addis Continental Institute of Public Health (ACIPH), Amhara Regional Health Bureau, Amhara Public Health Institute and Brigham and Women's Hospital. The Amhara region has low-resourced health systems and poor health indicators. As per the 2016 Ethiopian Demographic Health Services (EDHS) data, Amhara had the country's highest rates of neonatal mortality (47 per 1000 live births) and LBW (22.2%), and high maternal mortality rate (412 per 100,000 live births) [28].

Confidential: For Review Only

Rates of any prenatal care and institutional delivery and were 82.6% and 54.2%, respectively [29]. One in four women of reproductive age are underweight (body mass index<18.5 kg/m^2) [28] and geohelminth infections are prevalent, ranging from 21.1-43.5% [30,31].

The ENAT study is conducted in 12 rural health centers (each serving ~25,000 population) in West Gojjam (South and North Achefer districts [woredas]) and South Gondar (Dera and Libokemem districts) zones, Amhara (Figure 3, Site Map). The districts were chosen in collaboration with the Amhara Regional Health Bureau based on the high rates of undernutrition, risk of low birth weight, need for nutritional programs, and proximity to the regional laboratory. The study health centers were chosen based on accessibility, total ANC volume (minimum 250 women presenting to ANC/year), and infrastructure (functional laboratory).

Patient and public involvement

Prior to the study, formative work was conducted with a range of community members (mothers, families, community and religious leaders, health providers) [32]. This feedback directly informed the design of the study interventions, packages, and their implementation. Community sensitization was performed prior to initiating the study.

Study participants and recruitment

Pregnant women are recruited from ANC visits in designated ENAT study health centers. A study nurse explains study procedures and obtains written informed consent. For illiterate women, an impartial witness attests to consent and the woman provides a thumbprint.

To encourage early presentation at ANC clinics, community sensitization was conducted prior to study initiation. Study field data collectors and community cadres disseminated information about the ENAT study at monthly community-based pregnant women's conferences, and community and religious gatherings and encouraged presentation to the health centers if/as soon as pregnancy was suspected. Study enrollment began in August 2020 and will continue until the sample size has been met. As of September 1, 2021 ENAT has enrolled 2,148 women.

Inclusion criteria:

- ≤24 weeks gestation based on a clinical algorithm (LMP and/or symphysis fundal height) who have a viable pregnancy

Exclusion criteria:

https://mc.manuscriptcentral.com/bmjpo

Confidential: For Review Only

- Pregnant women presenting for first ANC >24 weeks
- Pregnant women who live >2 hours walking distance from ENAT health centers
- Pregnant women presenting at first ANC with fetus that is non-viable (without a heartbeat on enrollment ultrasound)

ENAT Study Interventions

Health systems strengthening of ANC services was performed in ENAT health centers prior to the study to benefit all women receiving care in these facilities. Health systems strengthening was conducted in partnership with Amhara Regional Health Bureau and local partners including Jhpiego. Staff were trained in ANC standards, guidelines, and measurements (blood pressure, gestational age, birth weight [33]). Facilities were stocked with basic equipment, medications, and diagnostic testing. Laboratory capacity was augmented, and all health centers were equipped with ultrasound machines.

A. Enhanced Nutrition Package (ENP) (Table 1)

Table 1: Enhanced Nutrition Package Components

ENP Content	Activity	Frequency
Nutritional education/counselling	Counselling about healthy eating, adequate pregnancy weight gain, increasing protein and energy in diet, importance of Iron/folate, iodized salt	Every ANC visit (monthly, potentially less frequently pending COVID)
Iron-Folic Acid	Strengthen counseling, supply and reinforcement of daily IFA (60 mg Fe/400 µg folic acid)	Provide 90-day supply per FMOH, adherence/counseling at every ANC visit
Iodized salt	For all pregnant women, provide up to 1kg at enrollment and follow up visit for household use during pregnancy with counselling	Provided adequately iodized salt at enrollment, and monthly follow up visits; adherence/counseling every ANC visit
Balanced Energy Protein supplement	For pregnant women with MUAC <23 cm, local Corn Soya Blend 200 gm daily supplement (784 kcal/day, 28 g protein)	Daily supplement, distributed at ANC visits (monthly, given COVID may provide longer term supply)

A.1 Nutritional Education/Counseling on Adequate Pregnancy Nutrition and Weight Gain:
Routine ANC nutrition counseling includes increasing intake and dietary diversity, however, can vary depending on patient load. For ENP centers, supplementary, locally contextualized, counseling was developed based on our formative work [32], and is delivered by midwives. Content includes BMI-based recommended weight gain, dietary diversity, and educational messages developed to address local cultural beliefs related to dietary intake during pregnancy

https://mc.manuscriptcentral.com/bmjpo
Confidential: For Review Only

as well as side effects of iron-folate. Nutrition education materials, including posters and videos, are shown in ENP health centers to promote women's behavior change and maximize their exposure to various but consistent nutrition messages.

A.2 Iron Folic Acid (IFA): The Ethiopian FMOH recommends 60 mg iron plus 400 µg folic acid supplements, orally once daily in pregnancy. In August-Sept 2019, coverage of IFA was 46.3% in the ENAT health centers, and our formative work indicated that barriers included local beliefs about "big babies" and side effects such as constipation. In the ENP health centers, additional counseling is conducted, utilizing video/media to address common cultural beliefs and side effects, and women are reminded at each ANC visit about IFA consumption, management of side effects and provided refills per FMOH guidelines.

A.3 Provision of Adequately Iodized Household Salt: In ENP centers, we provide a monthly household supply of high-quality adequately iodized salt at every ANC visit. The iodized salt (Waff Manufacturing, 30-40 ppm potassium iodate, 600 gm bottle) is packaged in airtight, resealable, polyethylene containers, to allow resealing after use and to reduce risk of evaporative losses at the household level. Quality control procedures are in place to ensure that iodization is in the proper range at production and maintained at distribution sites. Women are counselled that salt should be used to replace their household salt, on the approximate daily use (~10 gm (3 pinches)/day), the proper storage of salt (away from light, heat, humidity, re-capping container after use) and use of salt only after cooking/heating of food.

A.4 Fortified Balanced Energy Protein (BEP) Supplement for Malnourished Women (MUAC <23 cm): In the ENP health centers, women who have MUAC <23 cm at enrollment, or at any follow up ANC visit, are provided with a monthly supply of locally produced micronutrient fortified corn soya flour blend (Super Cereal, Faffa Food Share Company, Addis Ababa, Ethiopia) at every ANC visit until delivery. The daily corn soya blend (CSB) supplement (200gm) is provided in addition to normal meals and contains 28 g of protein and 784 kcal (Web Table 2). This protein composition falls within recommendations that a BEP supplement provide ~50% of the additional protein requirement in the 3rd trimester (range of 28-36g for malnourished populations) [34]. Micronutrient composition of the provided BEP is also shown in Web Table 2. The fortified BEP supplement meets the IOM recommended levels in pregnancy [35] for Vitamins A, D, E, B_2, B_3, B_6, B_{12}, C, calcium and phosphorus (see Web Table 2). 35 sachets (200 gm each) are distributed to women at enrollment and at follow up monthly ANC visits to

Confidential: For Review Only

allow for additional doses in case she is delayed in returning for ANC and/or for potential family sharing practices.

B. Enhanced Infection Management Package (EIMP) (Table 2)

Women randomised to the EIMP intervention, receive the following interventions at their first ANC visit >12 weeks.

B.1 Urinary Tract Infection/Asymptomatic Bacteriuria: A clean catch midstream urine specimen is collected using a vacutainer with boric acid preservative (Beckton Dickinson). Urine culture and antibiotic susceptibility testing are performed at Amhara Public Health Institute (APHI), the regional laboratory certified by the ENAO (Ethiopian National Accreditation Office) - ISO (International Organization for Standardization) 15189. Antibiotic susceptibility is determined including the Vitek method (bioMerieux, Marcy l'Etoile, France), or Kirby Bauer Disk Diffusion. Urinary tract infections (UTI) are classified in Web Table 3 and treated with an oral antibiotic based on antibiotic sensitivity patterns (Web Table 4). Antibiotics are provided to pregnant women at no cost and the first dose is directly observed. Women with severe illness or difficult to treat infections are referred to the obstetrics department at Feligot Hiwot hospital. Women provide a test of cure specimens at the following ANC visit.

Table 2. ENAT EIMP Components

Infection	ENAT Enhanced Infection Management Package (EIMP) Activity
Urinary Tract Infection/Asymptomatic Bacteriuria	*Screen:* Urine culture and antibiotic sensitivity.<br>*Treat:* Initially per clinical protocol, with targeted antibiotic treatment based on antibiotic resistance patterns.
Sexually Transmitted/ Reproductive Tract Infections	*Screen:*<br>1) **ALL pregnant women** for gonorrhea, chlamydia using accurate rapid diagnostic testing for chlamydia and gonorrhea<br>2) Pregnant women ***with symptoms*** are screened for trichomonas and bacterial vaginosis<br>*Treat:* All positive cases and partners (for gonorrhea, chlamydia, trichomonas)
Geo-helminths	Presumptive deworming with mebendazole 500 mg or albendazole 400 mg as per FMOH guideline; Stool screen and treatment for parasitic infections at least 4 weeks after deworming. If positive, treatment for intestinal parasites per FMOH guidelines.

Confidential: For Review Only

B.2 Sexually Transmitted/Reproductive Tract Infections: Women self-collect vaginal specimens that are tested for gonorrhea and chlamydia using the *Xpert® CT/NG assay* (*Cepheid*, Sunnyvale, CA), a modular cartridge-based platform for testing each specimen by nucleic acid amplification at APHI. Chlamydia is treated with Azithromycin 1 gram orally once and gonorrhea is treated with Ceftriaxone 250 mg IM once + Azithromycin 1gram orally once. Partner treatment is on a voluntary basis with a regimen as recommended by the Ethiopian STI management guidelines [36]. A test of cure is obtained at the next ANC visit.

For women who report symptoms of abnormal vaginal discharge, vulvar symptoms, or lower abdominal tenderness, additional vaginal swabs are collected for trichomonas and bacterial vaginosis by point of care diagnostics. Trichomonas is tested using the OSOM® trichomonas rapid test (Sekisui Diagnostics, Lexington, MA). Bacterial vaginosis is tested using Diagnosit® BVBLUE® test (Gryphus Diagnostics, Knoxville, TN)). Trichomonas is treated with Metronidazole 2 grams orally once, and partners are treated. Bacterial vaginosis is treated with metronidazole 500 mg twice daily for 7 days.

B.3. Parasitic Intestinal Infections: In settings of high geo-helminth burden, WHO recommends prophylactic deworming in 2nd and 3rd trimester of pregnancy [37]. At study initiation, mebendazole (500 mg) was provided twice in pregnancy consistent with WHO guidelines. Due to health provider concerns regarding medication package insert information contraindicating use in early pregnancy, in September 2020, the protocol was modified to a single presumptive deworming in the 3rd trimester. With the adoption of new Ethiopian FMOH ANC guidelines allowing earlier provision of anti-helminthics in pregnancy, in May 2021, the ENAT protocol was modified to provide presumptive deworming in the 2nd trimester followed by a stool screening and treatment at least 4 weeks later. In the first post-deworming visit, stool is screened for intestinal parasites in the health center lab using wet mount microscopy available at the health center. Women identified with parasitic infections are treated as per FMOH recommendations (Web Table 4).

Randomisation/Allocation

At the first level of randomisation, clusters (i.e., health centers) are randomised into one of two nutrition interventions: a) ENP or b) standard nutrition care. We performed a constrained randomisation to ensure balance across the two arms of the study for key

https://mc.manuscriptcentral.com/bmjpo
Confidential: For Review Only

indicators including: population size, pre-study ANC coverage rates, number of births, and travel time to the regional center of Bahir Dar. We: 1) set reasonable tolerance levels for the restriction variables, 2) created all possible random sequences, where each sequence allocated 6 health centers to the ENAT Nutrition Package and 6 health centers to routine care, 3) assessed each sequence as to whether or not it met these restriction criteria, and 4) choose randomly from the subset of all such allocation sequences that met the criteria. At the second level of randomisation, we randomised individual pregnant women presenting for ANC at each health center to receive a) *ENAT Enhanced Infection Management Package (EIMP)*, or b) standard infection care (Figure 2). Each health center received a pre-generated randomisation list of sequential individual assignments to EIMP or standard care, where assignments were equally allocated to each arm within randomly permuted blocks of size 4, 8, or 12. The randomisation lists were generated separately, by health center, using a script written by one of the authors (LCM) in R [38].

Outcome Measures

The **primary outcomes** are

- P1. Newborn weight measured within 72 hours of birth
- P2. Newborn length measured within 72 hours of birth

The **secondary outcomes** include:

- S1. Length of gestation, with gestational age determined by <24-week pregnancy ultrasonography
- S2. Proportion of pregnancies resulting in spontaneous preterm delivery
- S3. Proportion of newborns born of low birthweight (<2500 grams), as measured within 72 hours of life
- S4. Proportion of newborns born small-for-gestational age, as defined by the INTERGROWTH 21st neonatal birth weight standard.
- S5. Stillbirth rate
- S6. Newborn head circumference within 72 hours of birth
- S7. Infant Z-scores for weight-for-age, length-for-age, head circumference-for-age within 72 hours of birth
- S8. Maternal gestational weight gain
- S9. Maternal anemia (3rd trimester)

The definitions used for each outcome measure is shown in Table 3.

Confidential: For Review Only

Table 3: ENAT Study Outcomes

**Primary outcomes**	
**P1. Newborn weight <72 hours of birth**	Weight of the unclothed infant measured at <72 hours of life
**P2. Newborn length measured at birth**	Infant Length measured at <72 hours of life
**Secondary outcomes**	
**S1. Gestational age**	Gestational age determined by enrollment ultrasound, CRL used if <95 mm (Intergrowth 21st), then BPD/FL (WHO Kiserud) used if CRL>95 mm or missing
**S2a. Proportion of pregnancies resulting in preterm delivery**	Numerator: number of pregnancies resulting in spontaneous termination of pregnancy from 24 to <37 weeks (including preterm live birth or fetal loss (spontaneous pregnancy loss 24 to <37 weeks, not due to induced abortion)).<br>Denominator: All pregnancy outcomes >24 weeks
**S2b. Preterm birth**	Numerator: Live births <37 weeks of gestation<br>Denominator: Livebirths
**S3. Small for Gestational Age[a] (Intergrowth)**	<10% birthweight for GA by sex compared to Intergrowth reference[2]
**S4. Low birthweight rate**	Low birthweight is defined as birthweight (measured within the first 72 h of life) of <2500 g. We will also assess the outcome of birthweight <2000 g.
**S5. Stillbirth rate**	Numerator: Stillbirth (≥28 weeks gestation)- fetal death with no signs of life<br>*A preterm stillbirth is defined as an infant born without signs of life (no spontaneous crying, breathing, and/or movement) at 28 to <37 weeks gestation.*<br>*A term stillbirth is defined as an infant born without signs of life at ≥37 wks*<br><br>Denominator: All live births and stillbirths ≥28 weeks
**S6. Newborn head circumference**	Head circumference of the infant measured at <72 hours of age
**S7. Newborn weight, length, and head circumference for age Z-scores**	Infant weight, length, and head circumference for age z-scores measured at <72 hours of life, calculated using the Intergrowth reference for size at birth.
**S8. Rate of weight gain in pregnancy**	Maternal weight gain (kg) per week gestation in the 2nd and 3rd trimester
**S9. Maternal anemia**	Mean hemoglobin concentration in 3rd trimester<br>(Mission hemoglobinometer)

[a]Abbreviations: WHO, World Health Organization; UTI, urinary tract infection; ANC, antenatal care; SGA, small-for-gestational-age; GA, gestational age

Data Collection

Confidential: For Review Only

The timeline of individual participant study visits, measurements and data collection are shown in Table 4. All study visits are conducted at the health center, with the exception of the birth visit that may be conducted at home within 72 hours of delivery, for births occurring at home or outside of the study area. Adherence monitoring visits also occur at the home for those participants who do not return to the health center for follow up.

The core of the data collection system is the Survey Solutions platform (World Bank, v20.08, 2021). Study nurses enter data directly into electronic tablets with programmed validity checks during study visits. Paper forms are used if tablets are temporarily unavailable. The tablets are regularly synchronized to the server on the ACIPH campus. A web-based dashboard supports data collectors, supervisors, and investigators in real time management and monitoring of study activities.

Confidential: For Review Only

Table 4. Participant timeline schedule of enrolment, interventions, assessment, and visits

	STUDY PERIOD						
		Post-allocation					
	Allocation	Prenatal				Postnatal	
**TIMEPOINT**	**Enrolment <24 weeks**	***ANC1***	***ANC2***	***ANC3***	***etc.***	***Birth***	***1 month***
**ENROLMENT:**							
**Eligibility screen**	X						
**Informed consent**	X						
**Allocation**	X						
**INTERVENTIONS:**							
**ENP**	X	X	X	X	X		
**EIMP**	X	X	X	X	X		
**ENP + EIMP**	X	X	X	X	X		
**ASSESSMENTS:**							
MOTHERS							
US (fetal growth & GA determination)	X			X			
Basic medical & obstetric history	X						
Socioeconomic status	X						
Health care costs	X		X				
Food insecurity and Dietary Intake	X	X		X			
Maternal stress and depression		X		X			X
Maternal anthropometrics	X	X	X	X	etc.	X	X
Maternal morbidity	X	X	X	X	etc.	X	X
Labor and Delivery characteristics						X	
Assessment of home environment						X	
INFANTS							
Anthropometrics						X	X
Breast feeding practices						X	X
Morbidity and mortality						X	X

Confidential: For Review

Enrollment Visit

At the enrollment visit at the health center, data is collected on the participant's socio-economic status, basic medical and obstetric history, pregnancy history, maternal morbidity including COVID-19, food security, and dietary intake. A dietary quality questionnaire is administered, which has been used in the Ethiopian context [39].

A basic abdominal obstetric ultrasound is performed by a trained research nurse at the enrollment visit for pregnancy dating. An intensive ultrasonography training and standardization was performed by General Electric, Ethiopian Radiography Association and sonographers from Beth Israel Deaconess Hospital (Boston, MA). Sonographers measure crown-rump length, bi-parietal diameter (outer to inner), head circumference, femoral length, abdominal circumference in duplicate. Approximately 10% of images are externally reviewed (MS, BJW) for quality control.

Maternal and infant anthropometrics are measured by research staff (nurses, data collectors) at baseline and follow up visits (Table 3). Maternal weight is measured with a digital scale (ADE M317600, Germany; precision 100 gm) and height is measured using an adult stadiometer (Shorr Productions HeightLite). All measurements are performed twice, with a third measurement done if the difference is greater than the minimal acceptable difference defined by Intergrowth 21 [40]. Nurses and data collectors are trained and standardized in anthropometric measurements at the start of the study and every 6 months.

Follow up ANC Visits

During the follow up ANC visits at the health center, research staff interview women about their health status, morbidity, pregnancy history/complications, counselling/services received, maternal mental health screen, and dietary intake. Data is abstracted from routine ANC records, including blood pressure, lab testing results, and management. Maternal weight and mid-upper arm circumference are measured. In a subset of women, a semi-quantitative food frequency questionnaire for ~70 food items is administered at ANC visits. Hemoglobin is measured during enrollment in the 1st or 2nd and 3rd trimester ANC visits (Hemocue 301c). Venous hemoglobin is measured if blood is already being drawn for other purposes, and otherwise capillary hemoglobin is measured.

Confidential: For Review Only

Adherence to each nutritional supplement is assessed at every ANC visit. The participant is asked to bring the used IFA bottle, salt container, and BEP sachets back to the health center at each ANC visit. Pill or empty sachet count is done, and the salt container weighed. The participant is also asked to recall the number of sachets and/or pills that were taken in the last 7 days, and since the last visit. For mothers who do not attend scheduled ANC visits, a home visit is made by a data collector to assess adherence and conduct pill/sachet counts and remind the mother to return for ANC and study visits.

A repeat ultrasound is performed in the 3rd trimester to monitor fetal growth and assess the position of the baby. If the fetus is determined to be in non-vertex positioning at the 3rd trimester scan, the nurse recommends that the women deliver in the nearest hospital with Cesarean section capacity.

Birth Visit

Participants who deliver in health facilities are assessed by research staff based in health centers or hospitals as soon as possible after birth, but within 72 hours of life. Data is gathered from women and from chart review about the delivery and immediate postpartum period. Medical records are reviewed for intrapartum course (e.g., vital signs, duration of labor), delivery complications, and maternal/neonatal morbidity. For deliveries that occur at home, a home visit is made by research staff as soon as possible upon birth notification (within 72 hours). Maternal history is obtained per self-report regarding delivery history/complications, and postpartum maternal/neonatal morbidity.

Infant weight is measured using a high quality, precise digital infant scale (ADE M112600, Germany; precision 5 gm). Infant length is measured using a portable infantometer (Perspective Enterprises PE-RILB-LTWT, Michigan USA, precision 1 mm). Recumbent length is recorded to the last completed (not the nearest) mm. Head, chest, and mid upper arm circumferences (MUAC) is measured to the nearest millimeter (mm) using insertion tapes (Shorr productions, Maryland USA). Daily calibration checks are made before each use of infant weighing scales, and length boards to ensure accuracy of measurement [41,42].

Postnatal Visit

Confidential: For Review Only

Postnatal visits are made at 4-6 weeks for all participants to collect data on maternal and infant vital status, health, morbidity and anthropometrics. Visits are conducted primarily at the health center, and home visits are made for those who do not return for follow up. For infants who follow up at the health center for routine postnatal care or immunizations, study visits are additionally conducted at 3 and 6 months.

Cost Data

Data regarding the costs of delivery of the ENAT study interventions is collected in all study arms. Costs of interventions include three components: system-level costs, costs incurred by health workers for participating in the interventions, and costs incurred by individual patients and families. At the system level, costs are captured using the World Health Organization (WHO) framework [43] using modified survey tools based on our published survey instruments, cost estimation protocols, and procedures that have been validated and used in other LMICs (e.g., Rwanda, India) [44–46]; Costs incurred by health workers for receiving training include time or money spent for participating in training sessions. For all time spent, monetary value is assigned based on their average hourly wage. Costs incurred by patients include costs or time spent for received care or home visits. For visits in health facilities, we collect self-reported cost data from patients.

Biospecimens: In a subset of consenting women, additional biospecimens will be collected for future analysis. These specimens are shown in Web Table 5.

Statistical Analysis

Data collected in this study will enable us to conduct a comprehensive analysis of the impact of interventions randomized at the health center and individual level. The Detailed Statistical Analysis Plan (SAP) of the ENAT study is published separately at: https://addiscontinental.edu.et/. In brief, our statistical approach will have multiple steps. We will describe the health centers and pregnant women enrolled in the study and conduct a descriptive quantitative analysis of variables at multiple levels to assess the degree to which our randomization scheme resulted in similar sub-populations of pregnant women. We will assess the receipt of and adherence to interventions offered, conduct descriptive analyses of the primary and secondary outcomes, compare the outcomes between intervention groups, assess potential effect modifiers, and conduct pre-specified sub-group analysis.

Confidential: For Review Only

Sample Size

We have estimated the effect size detectable with 80% power under a cluster-randomised design, with 6 health centers per study arm. Fixing recruitment of pregnant women to 18 months, we estimated that within this time period the average health center in the proposed study site would enroll around 200 women into ANC at ≤24 weeks gestation, and would yield 112 live born infants weighed within 72 hours of life (assuming ~70% of enrolled pregnancies result in a live birth, and ~80% are followed up and weighed within 72 hours). Beyond the above determination of average cluster size, we have additionally made the following assumptions in order to estimate effect sizes detectable with 80% power: 1) mean birthweight and standard deviation as per prior studies in Gondor (mean birth weight of 2900 gm, SD 450gm) and 2) variation in distribution of weight between clusters as reflected through an coefficient of variation (k=0.01) [47]. In total this includes 2,400 pregnant mothers enrolled in 12 health centers, resulting in 1,440 live births with a birthweight within 72 hours. This sample size provides 80% power to detect a 66 gram difference in birthweight between the ENAT EIMP or routine care group in a marginal analysis (i.e. irrespective of whether mothers did or did not receive the ENP), and a 90 gm difference between ENP vs routine care (marginal analysis).

Length. With the assumptions of clusters and enrollment above, we assumed mean infant length of 49.5 cm (SD 2.4) (based on the Malawi LAIS study [48,49] and coefficient of variation k=0.008 (sector level variation in JiVitA study) [47]. For the EIMP vs. routine care comparison, we would have 80% power to detect a 3.0 mm difference in mean infant length. For the ENP vs. non-ENP comparison (marginal analysis) we would have 80% power to detect a 7.8 mm difference in infant length between the women receiving the package of enhanced nutrition-infection compared to standard nutrition care.

Study Monitoring

An external Study Monitoring Committee (SMC) is established to monitor the progress of the study, including enrollment, progress indicators and adverse events. The SMC includes an independent Ethiopian obstetrician (Dr. Delayehu Bekele) and an epidemiologist (Professor Simon Cousens). The committee met before study initiation, at mid-enrollment, and every 6 months to review study progress. Interim analysis will not be performed.

Confidential: For Review Only

ETHICS AND DISSEMINATION

Research Ethics approval: The ENAT protocol was approved at Addis Continental Institute of Public Health (001-A1-2019) and Mass General Brigham Institutional Review Board (2018P002479). The APHI granted local permission to conduct the study.

The study is registered at ISRCTN (ISRCTN15116516). Protocol modifications will be communicated to respective IRBs and ISRCTN.

Confidentiality

All the data collected for this study is kept strictly confidential at Addis Continental Institute of Public Health on a local encrypted server. Personal identifiers are not used in study-specific forms, aside from the identifier module. Paper copies of data forms for data entry and analysis are stored in a locked file when not in use. Access to data files containing personal identifying information is limited to the principal investigators and key staff.

Ancillary care

ENAT project helps cover ancillary care related to study participation that is not covered by the health system.

Dissemination plan

ENAT study findings will be disseminated to FMoH, APHI, ARHB, Zonal health departments, woreda health offices, community representatives, and to other relevant stakeholders. Involving relevant stakeholders in the dissemination process will help to enhance ownership of the research output and the ultimate integration of findings into programs. The dissemination will be in the form of presentations in workshops, conferences, and symposiums at local, regional, national, and international levels as appropriate. In addition, reports and peer-review journal publications will be produced.

DISCUSSION

Despite the high global burden of low birthweight, preterm birth and fetal growth restriction, few interventions have demonstrated efficacy or effectiveness in the prevention of these adverse birth outcomes. Novel, integrated approaches are needed to make more substantial, scalable and sustainable impacts in order to meet the WHO's Nutrition targets. A critical feature of the ENAT study is its health systems approach and involvement of key local stakeholders in the design of intervention packages and implementation. The packaging/bundling of interventions

Confidential: For Review Only

aims to maximize their potential impact, cost-effectiveness, learning, and future scalability. This study will provide important evidence on the role of strengthening ANC programs to improve maternal health and birth outcomes, and will help inform future ANC policies in Ethiopia. ENAT will contribute to the growing body of knowledge regarding the effectiveness of BEP supplementation during pregnancy. Secondly, despite the high burden of antenatal infections, there is limited high-quality evidence that interventions to treat prenatal infections improves birth outcomes. To our knowledge, there is even more limited data on the interactions of nutrition and infections in pregnancy and this study will provide novel new insight on the potential for synergistic benefits of an integrated approach. Finally, the study will generate implementation learning on how to optimize delivery of WHO guidelines in resource limited health systems.

Access to data

The final dataset will be available to the ENAT investigative team and collaborators. Anonymized study data may be made available upon request to the study PIs in accordance with Ethiopian and US regulatory guidelines.

Declaration of interests

Financial and competing interests of the PIs

ACL reports funding from the Bill & Melinda Gates Foundation, Eunice Kennedy Shriver National Institute of Child Health and Human Development and the World Health Organization. YB reports funding from FORMAS, USAID, National Institute of Environmental Health Sciences and from the Bill & Melinda Gates Foundation. BJW reports funding from National Institute of Environmental Health Sciences and from the Bill & Melinda Gates Foundation.

Funding Statement: This work was supported by the Bill & Melinda Gates Foundation grant number OPP1184363. The funder provided input on the study design, but played no role in the data collection, data analysis, interpretation of data, writing of the report or decision to submit the report for publication.

Acknowledgements

We would like to thank the ENAT partners for their support and contributions to the study, including the Amhara Regional Health Bureau, Amhara Public Health Institute, Ethiopian Federal Ministry of Health, Ethiopian Society of Obstetrics and Gynecology, General Electric, Ethiopian Society of Radiology, and Jhpiego. We acknowledge the Child Investment Fund who supported Jhpiego health systems strengthening efforts in study areas. We would also like to thank our Study Monitoring Committee, Dr. Delayehu Bekele and Professor Simon Cousens.

Author Contributions

Wrote manuscript: ACL, LCM, FW, ME, YB

Designed study/protocol: ACL, FW, LCM, EB, ME, GC, PC, SI, CL, RM, AWT, BJW, AQ, YB

Confidential: For Review Only

Designed study materials/study tools: ACL, FW, EB, YB, MD, ME, NF, IO, WK, TS, FS, FT, ST, KY, SI, YK, CL, RM, MS, FV, AWT, YB
Trained field data collectors: FW, EB, YB, MD, ME, WK, TS, FS, FT, ST, KY, AWT, ACL, YB
Designed Data management system: NF, FS, FT, FV, AW
Trained ultrasonography: MS, BW, KY, ST
Critically reviewed/revised manuscript: all authors
All authors have met ICJME criteria.

FIGURE CAPTIONS

Figure 1. **Conceptual diagram showing the pathways that link maternal undernutrition, maternal infection, and infant outcomes** (A, B, and C represent mechanisms, which are described in detail in the associated sections above)

Figure 2. ENAT Study Consort Diagram

Figure 3. ENAT Study Site Map, Amhara Region, Ethiopia

Confidential: For Review Only

REFERENCES

1 World Health Organization. Global nutrition targets 2025: low birth weight policy brief. Geneva, Switzerland: 2014.

2 Blencowe H, Krasevec J, de Onis M, *et al.* National, regional, and worldwide estimates of low birthweight in 2015, with trends from 2000: a systematic analysis. *Lancet Glob Health* 2019;**7**:e849–60. doi:10.1016/S2214-109X(18)30565-5

3 Katz J, Lee AC, Kozuki N, *et al.* Mortality risk in preterm and small-for-gestational-age infants in low-income and middle-income countries: a pooled country analysis. *Lancet* 2013;**382**:417–25. doi:10.1016/S0140-6736(13)60993-9

4 Christian P, Lee SE, Donahue Angel M, *et al.* Risk of childhood undernutrition related to small-for-gestational age and preterm birth in low- and middle-income countries. *Int J Epidemiol* 2013;**42**:1340–55. doi:10.1093/ije/dyt109

5 Blencowe H, Lee ACC, Cousens S, *et al.* Preterm birth-associated neurodevelopmental impairment estimates at regional and global levels for 2010. *Pediatr Res* 2013;**74 Suppl 1**:17–34. doi:10.1038/pr.2013.204

6 Murray E, Fernandes M, Fazel M, *et al.* Differential effect of intrauterine growth restriction on childhood neurodevelopment: a systematic review. *BJOG* 2015;**122**:1062–72. doi:10.1111/1471-0528.13435

7 Danaei G, Andrews KG, Sudfeld CR, *et al.* Risk factors for childhood stunting in 137 developing countries: A comparative risk assessment analysis at global, regional, and country levels. *PLoS Med* 2016;**13**:e1002164. doi:10.1371/journal.pmed.1002164

8 Han Z, Mulla S, Beyene J, *et al.* Maternal underweight and the risk of preterm birth and low birth weight: a systematic review and meta-analyses. *Int J Epidemiol* 2011;**40**:65–101. doi:10.1093/ije/dyq195

9 Kozuki N, Katz J, Lee ACC, *et al.* Short Maternal Stature Increases Risk of Small-for-Gestational-Age and Preterm Births in Low- and Middle-Income Countries: Individual Participant Data Meta-Analysis and Population Attributable Fraction. *J Nutr* 2015;**145**:2542–50. doi:10.3945/jn.115.216374

10 Peña-Rosas JP, De-Regil LM, Garcia-Casal MN, *et al.* Daily oral iron supplementation during pregnancy. *Cochrane Database Syst Rev* 2015;:CD004736. doi:10.1002/14651858.CD004736.pub5

Confidential: For Review Only

11 Keats EC, Neufeld LM, Garrett GS, *et al.* Improved micronutrient status and health outcomes in low- and middle-income countries following large-scale fortification: evidence from a systematic review and meta-analysis. *Am J Clin Nutr* 2019;**109**:1696–708. doi:10.1093/ajcn/nqz023

12 Imdad A, Bhutta ZA. Maternal nutrition and birth outcomes: effect of balanced protein-energy supplementation. *Paediatr Perinat Epidemiol* 2012;**26 Suppl 1**:178–90. doi:10.1111/j.1365-3016.2012.01308.x

13 Ota E, Hori H, Mori R, *et al.* Antenatal dietary education and supplementation to increase energy and protein intake. *Cochrane Database Syst Rev* 2015;:CD000032. doi:10.1002/14651858.CD000032.pub3

14 Buppasiri P, Lumbiganon P, Thinkhamrop J, *et al.* Calcium supplementation (other than for preventing or treating hypertension) for improving pregnancy and infant outcomes. *Cochrane Database Syst Rev* 2011;:CD007079. doi:10.1002/14651858.CD007079.pub2

15 Gilbert NM, O’Brien VP, Hultgren S, *et al.* Urinary tract infection as a preventable cause of pregnancy complications: opportunities, challenges, and a global call to action. *Glob Adv Health Med* 2013;**2**:59–69. doi:10.7453/gahmj.2013.061

16 Romero R, Oyarzun E, Mazor M, *et al.* Meta-analysis of the relationship between asymptomatic bacteriuria and preterm delivery/low birth weight. *Obstet Gynecol* 1989;**73**:576–82.

17 Honkpéhèdji YJ, Adegbite BR, Zinsou JF, *et al.* Association of low birth weight and polyparasitic infection during pregnancy in Lambaréné, Gabon. *Trop Med Int Health* 2021;**26**:973–81. doi:10.1111/tmi.13591

18 Aderoba AK, Iribhogbe OI, Olagbuji BN, *et al.* Prevalence of helminth infestation during pregnancy and its association with maternal anemia and low birth weight. *Int J Gynaecol Obstet* 2015;**129**:199–202. doi:10.1016/j.ijgo.2014.12.002

19 Abioye AI, McDonald EA, Park S, *et al.* Maternal, placental and cord blood cytokines and the risk of adverse birth outcomes among pregnant women infected with Schistosoma japonicum in the Philippines. *PLoS Negl Trop Dis* 2019;**13**:e0007371. doi:10.1371/journal.pntd.0007371

20 Goldenberg RL, Hauth JC, Andrews WW. Intrauterine infection and preterm delivery. *N Engl J Med* 2000;**342**:1500–7. doi:10.1056/NEJM200005183422007

21 Christensen DL, Kapur A, Bygbjerg IC. Physiological adaption to maternal malaria and other adverse exposure: low birth weight, functional capacity, and possible metabolic disease in adult life. *Int J Gynaecol Obstet* 2011;**115 Suppl 1**:S16-9. doi:10.1016/S0020-7292(11)60006-4

Confidential: For Review Only

22 Krawinkel MB. Interaction of nutrition and infections globally: an overview. *Ann Nutr Metab* 2012;**61 Suppl 1**:39–45. doi:10.1159/000345162

23 Vohr BR, Poggi Davis E, Wanke CA, *et al.* Neurodevelopment: The Impact of Nutrition and Inflammation During Preconception and Pregnancy in Low-Resource Settings. *Pediatrics* 2017;**139**:S38–49. doi:10.1542/peds.2016-2828F

24 Kim H, Hwang JY, Ha EH, *et al.* Association of maternal folate nutrition and serum C-reactive protein concentrations with gestational age at delivery. *Eur J Clin Nutr* 2011;**65**:350–6. doi:10.1038/ejcn.2010.267

25 Kutlesic V, Brewinski Isaacs M, Freund LS, *et al.* Executive Summary: Research Gaps at the Intersection of Pediatric Neurodevelopment, Nutrition, and Inflammation in Low-Resource Settings. *Pediatrics* 2017;**139**:S1–11. doi:10.1542/peds.2016-2828C

26 Collin SM, Baggaley RF, Pittrof R, *et al.* Could a simple antenatal package combining micronutritional supplementation with presumptive treatment of infection prevent maternal deaths in sub-Saharan Africa? *BMC Pregnancy Childbirth* 2007;**7**:6. doi:10.1186/1471-2393-7-6

27 World Health Organization (WHO). WHO recommendations on antental care for a positive pregnancy experience. 2016.

28 Central Statistical Agency CSAE, Icf. Ethiopia Demographic and Health Survey 2016. Addis Ababa, Ethiopia: : CSA and ICF 2017.

29 Ethiopia Mini Demographic and Health Survey 2019: Key indicators. Rockville, Maryland, USA: EPHI and ICF: : Ethiopian Public Health Institute (EPHI) [Ethiopia] and ICF 2019.

30 Shiferaw MB, Zegeye AM, Mengistu AD. Helminth infections and practice of prevention and control measures among pregnant women attending antenatal care at Anbesame health center, Northwest Ethiopia. *BMC Res Notes* 2017;**10**:274. doi:10.1186/s13104-017-2609-6

31 Belyhun Y, Medhin G, Amberbir A, *et al.* Prevalence and risk factors for soil-transmitted helminth infection in mothers and their infants in Butajira, Ethiopia: a population based study. *BMC Public Health* 2010;**10**:21. doi:10.1186/1471-2458-10-21

32 Workneh F, Eglovitch M, Shiferaw T, *et al.* Dietary practices among pregnant women in rural amhara, ethiopia. *Curr Dev Nutr* 2021;**5**:698–698. doi:10.1093/cdn/nzab045_080

Confidential: For Review Only

33 Baye E, Abate FW, Eglovitch M, *et al.* Effect of birthweight measurement quality improvement on low birthweight prevalence in rural Ethiopia. *Popul Health Metr* 2021;**19**:35. doi:10.1186/s12963-021-00265-0

34 Members Of An Expert Consultation On Nutritious Food Supplements For Pregnant And Lactating Women. Framework and specifications for the nutritional composition of a food supplement for pregnant and lactating women (PLW) in undernourished and low income settings. *Gates Open Res* Published Online First: 2019. doi:10.21955/gatesopenres.1116379.1

35 Institute of Medicine. Dietary Reference Intakes (DRIs): Recommended Dietary Allowances and Adequate Intakes. Washington, DC: The National Academies Press

36 Federal Democratic Republic of Ethiopia Ministry of Health. National Guidelines for the Management of Sexually Transmitted Infections Using Syndromic. Addis Ababa, Ethiopia: 2015.

37 World Health Organization. Soil-transmitted helminth infections. 2020.https://www.who.int/news-room/fact-sheets/detail/soil-transmitted-helminth-infections (accessed 8 Sep2021).

38 R Core Team. *R: A language and environment for statistical computing*. Vienna, Austria: R Foundation for Statistical Computing 2021.

39 Workicho A, Belachew T, Feyissa GT, *et al.* Household dietary diversity and Animal Source Food consumption in Ethiopia: evidence from the 2011 Welfare Monitoring Survey. *BMC Public Health* 2016;**16**:1192. doi:10.1186/s12889-016-3861-8

40 International Fetal and Newborn Growth Consortium. The International Fetal and Newborn Growth Standards for the 21st Century(INTERGROWTH-21st) Study Protocol. 2009.

41 de Onis M, Onyango AW, Borghi E, *et al.* Comparison of the World Health Organization (WHO) Child Growth Standards and the National Center for Health Statistics/WHO international growth reference: implications for child health programmes. *Public Health Nutr* 2006;**9**:942–7. doi:10.1017/phn20062005

42 Bloem M. The 2006 WHO child growth standards. *BMJ* 2007;**334**:705–6. doi:10.1136/bmj.39155.658843.BE

43 The world health report 2000 - Health systems: improving performance. Geneva, Switzerland: : World Health Organization 2000.

Confidential: For Review Only

44 Lu C, Tsai S, Ruhumuriza J, *et al.* Tracking rural health facility financial data in resource-limited settings: a case study from Rwanda. *PLoS Med* 2014;**11**:e1001763. doi:10.1371/journal.pmed.1001763

45 Tsai S, Umugiraneza G, Ruhumuriza J, *et al.* Training manual on tracking financial data in health facilities in Kayonza and Kirehe. Boston, MA: : Program in Global Health Economics and Social Change, Harvard Medical School 2013.

46 Lu C, Tsai S, Ruhumuriza J, *et al.* Obtaining financing data for local health facilities in two districts of Rwanda: General guidelines. Boston, MA: : Program in Global Helath Economics and Social Change, Harvard Medical School 2013.

47 West KP, Shamim AA, Mehra S, *et al.* Effect of maternal multiple micronutrient vs iron-folic acid supplementation on infant mortality and adverse birth outcomes in rural Bangladesh: the JiVitA-3 randomized trial. *JAMA* 2014;**312**:2649–58. doi:10.1001/jama.2014.16819

48 Luntamo M, Kulmala T, Mbewe B, *et al.* Effect of repeated treatment of pregnant women with sulfadoxine-pyrimethamine and azithromycin on preterm delivery in Malawi: a randomized controlled trial. *Am J Trop Med Hyg* 2010;**83**:1212–20. doi:10.4269/ajtmh.2010.10-0264

49 Luntamo M, Kulmala T, Cheung YB, *et al.* The effect of antenatal monthly sulphadoxine-pyrimethamine, alone or with azithromycin, on foetal and neonatal growth faltering in Malawi: a randomised controlled trial. *Trop Med Int Health* 2013;**18**:386–97. doi:10.1111/tmi.12074

Confidential: For Review Only
